# Discovery of the SARS-CoV‑2 Papain-Like Protease Inhibitor MR1–114: From Structure-Based Design to *In Vivo* Antiviral Efficacy

**DOI:** 10.1021/acs.jmedchem.5c03846

**Published:** 2026-03-26

**Authors:** Malla Reddy Gannarapu, Divakar Indukuri, Cameron Holberg, Kiira Ratia, Omar Lozano Ramos, Savio Cardoza, Ganga Reddy Velma, Soumya Reddy Musku, Zuohuang Qi, Steve Slilaty, Zuomei Li, Lijun Rong, Gregory R J Thatcher, Rui Xiong

**Affiliations:** 1 Department of Pharmacology & Toxicology, R. Ken Coit College of Pharmacy, 8041 University of Arizona, Tucson, Arizona 85721, United States; 2 Department of Chemistry & Biochemistry, Colleges of Science and Medicine, 8041 University of Arizona, Tucson, Arizona 85721, United States; 3 Research Resources Center, University of Illinois Chicago (UIC), Chicago, Illinois 60612, United States; 4 Sunshine Biopharma Inc., 333 Las Olas Way, CU4 Suite 433, Fort Lauderdale, Florida 33301, United States; 5 Department of Microbiology, College of Medicine, University of Illinois (UIC), Chicago, Illinois 60612, United States

## Abstract

The SARS-CoV-2 papain-like protease (PLpro) is a critical target for antiviral intervention. Here, we report the discovery of MR1–114 (compound **15**), a potent, noncovalent PLpro inhibitor. Guided by structure-based design, we incorporated a strategic alkyne linker hypothesized to access the cryptic Val70^Ub^ pocket, a modification that proved critical for driving cellular potency. Multiparameter optimization yielded MR1–114, which displays a desirable ADME profile characterized by high solubility, permeability, and hepatocyte stability, translating to high oral bioavailability in mice and rats. Notably, the compound exhibits preferential lung enrichment in mice and rats while maintaining broad-spectrum activity against variants, including Delta and Omicron BA.5 with nanomolar potency *in vitro*. In the K18-hACE2 mouse model, oral administration of MR1–114 matched the therapeutic efficacy of nirmatrelvir, significantly suppressing pulmonary viral replication and preventing disease-associated weight loss.

## Graphical abstract

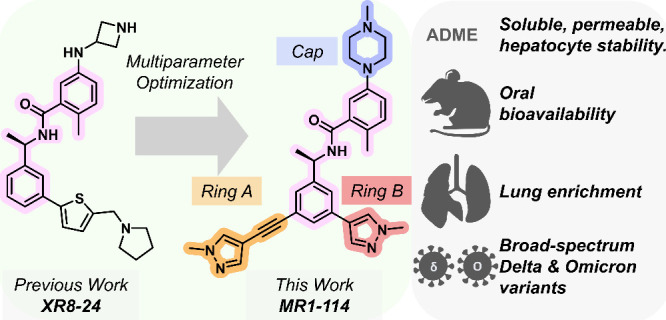

## Introduction

The COVID-19 pandemic, driven by the novel severe acute respiratory syndrome coronavirus 2 (SARS-CoV-2), has inflicted severe socioeconomic damage worldwide, resulting in more than 800 million infections and an estimated 7 million deaths.^1–4^ Unprecedented collective scientific efforts leading to the rapid development and deployment of therapeutic antibodies,^5^ vaccines,^6^ and antivirals,^7–9^ have greatly helped control infections. However, CDC wastewater surveillance data^10^ continues to show substantial levels of SARS-CoV-2 RNA, and, with a high mutation rate that yields nearly two genetic changes per month,^11^ SARS-CoV-2 and emerging variants of concern require ongoing basic and translational research.

To date, the U.S. Food and Drug Administration (FDA) has approved three small-molecule antiviral drugs for treatment of SARS-CoV-2. The first two, remdesivir (Veklury) and molnupiravir (Lagevrio), target the viral RNA-dependent RNA polymerase (RdRp), yet their clinical efficacy remains modest.^8,9^ The third drug, Paxlovid, has transformed acute care of anti-SARS-CoV-2 infection^12^ and appears to lower the risk of post-COVID-19 condition, often called long COVID.^13^ The World Health Organization strongly recommends Paxlovid for patients at moderate to high risk of hospitalization. This regimen combines nirmatrelvir, which inhibits the SARS-CoV-2 main protease (Mpro or 3CLpro), with ritonavir, an HIV protease inhibitor that blocks CYP3A4 metabolism to boost nirmatrelvir levels. Although Paxlovid has reduced mortality by around 61% in real-world hospital settings,^14^ not every patient responds fully, and some require alternative treatments. In addition, ritonavir’s inhibition of CYP3A4 creates unpredictable drug interactions, limiting Paxlovid’s use in pediatric populations and elderly patients with comorbidities.

Alongside 3CLpro and RdRp, the papain-like protease (PLpro, part of nsp3) represents a valuable target for SARS-CoV-2 antivirals.^15–17^ PLpro and Mpro both cleave the viral polyprotein, but whereas Mpro cuts at 11 sites, PLpro recognizes the LXGG motif at three sites to release nsp1, nsp2, and nsp3. PLpro spans residues 1602–1855 of the 1922-amino-acid nsp3 (approximately 215 kDa).^18,19^ The cysteine protease activities of both enzymes are essential for viral replication, making them attractive targets for drug development. These proteases also mutate far less frequently than the spike protein, which further underscores their suitability as targets against emerging variants. Beyond its role in polyprotein processing, PLpro supports viral replication by disrupting the host’s innate immune defenses: It functions as a deubiquitinase that removes ubiquitin and ubiquitin-like modifiers, most notably ISG15, from host proteins, thereby undermining a key antiviral response.^20–26^

Although PLpro has long been viewed as a promising antiviral target, discovering potent inhibitors with reliable *in vivo* efficacy remains challenging. Building on early work with naphthalene derivatives such as GRL0617,^27^ our group discovered that the highly dynamic BL2 loop can adopt a druggable groove. We confirmed this BL2 groove through our own cocrystal structures and used these insights to design XR8–23, a BL2-groove-binding inhibitor that concentrates in lung tissue by as much as 10-fold and shows antiviral efficacy in animal models (Figure 1).^28,29^ Another noncovalent inhibitor, PF-07957472, also targets the BL2 groove via a pyrazole moiety and demonstrates strong *in vivo* activity.^30^ By contrast, Jun12682 engages both the BL2 groove and an alternative Val70^Ub^ pocket contralateral to the BL2 groove. The Val70^Ub^ pocket mimics the interaction of ubiquitin’s hydrophobic residues Val70 and Leu71 with PLpro and delivers impressive biochemical, cellular, and *in vivo* potency.^31^ A recent high-throughput screen identified WEHI-P8, which locks the BL2 loop in a distinct conformation and exhibits notable *in vivo* efficacy.^32^ Additional inhibitors were reported and reviewed recently (Figure 1).^33–45^ In this study we present a novel series of noncovalent PLpro inhibitors building upon XR8–23 and XR8–24 that bind both the BL2 groove and the Val70^Ub^ pocket. These compounds exhibit strong potency, favorable ADME properties, and high oral bioavailability, and they provide robust efficacy in the K18-hACE2 mouse model of SARS-CoV-2 infection.

**Figure 1:**
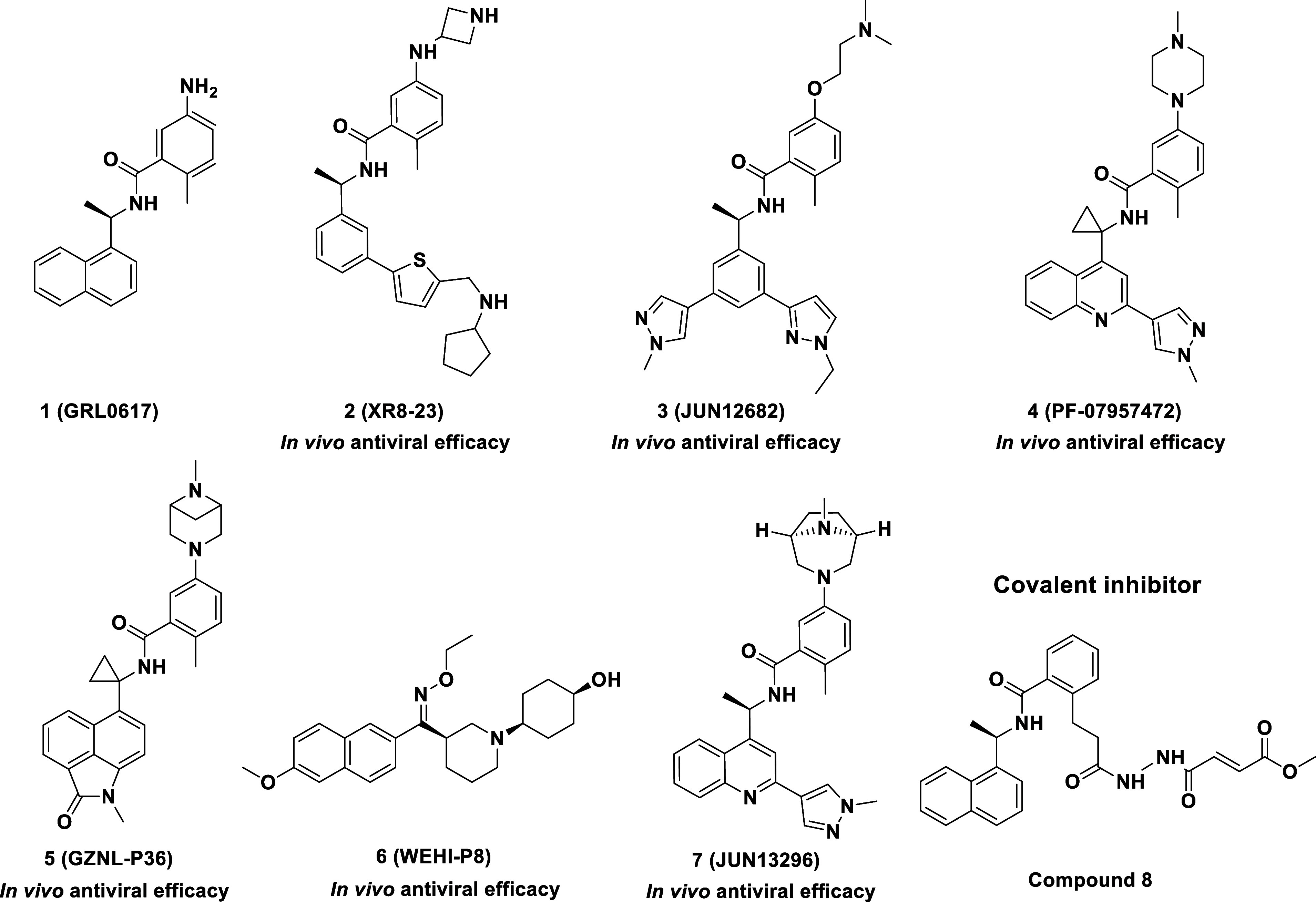
Structures of representative SARS-CoV-2 PLpro inhibitors, including key compounds with established *in vivo* efficacy.

## Results and Discussion

### Design Strategy

Building on our previously reported BL2-groove targeting 2-thiophenephenyl series (exemplified by compound XR8–24),^28,29^ we used our in-house cocrystal structures to refine the design strategy (PDB: 7LBS, Figure 2). Detailed inspection revealed a significant, unoccupied hydrophobic volume positioned contralateral to the primary BL2 groove interaction site, corresponding to the Val70^Ub^-binding pocket (Figure 2A). Notably, this pocket accommodates ubiquitin’s hydrophobic residues Val70 and Leu71 during PLpro recognition, suggesting an opportunity to mimic these contacts to enhance binding. To rationalize chemistry, we define the aromatic core directed into the Val70^Ub^ pocket as the Ring A, the BL2-groove engaging aromatic unit as the Ring B, and the solvent-exposed amine region as the Cap. We hypothesized that introducing an appropriately oriented linker to connect the central scaffold to an Ring A pharmacophore engaging the Val70^Ub^ pocket would enable a cooperative binding mode that significantly improves overall potency. This structure-based rationale converges with the recent findings of Tan et al.,^31^ in which the potent inhibitor Jun12682 exploits a similar pocket to boost potency. Guided by these structural insights (Figure 2B), we pursued a stepwise, multiparameter optimization of the XR8–24 scaffold, prioritizing the linker, Ring A, and Ring B, followed by cap modifications, B-ring reoptimization, and chiral side-chain fine-tuning.

**Figure 2:**
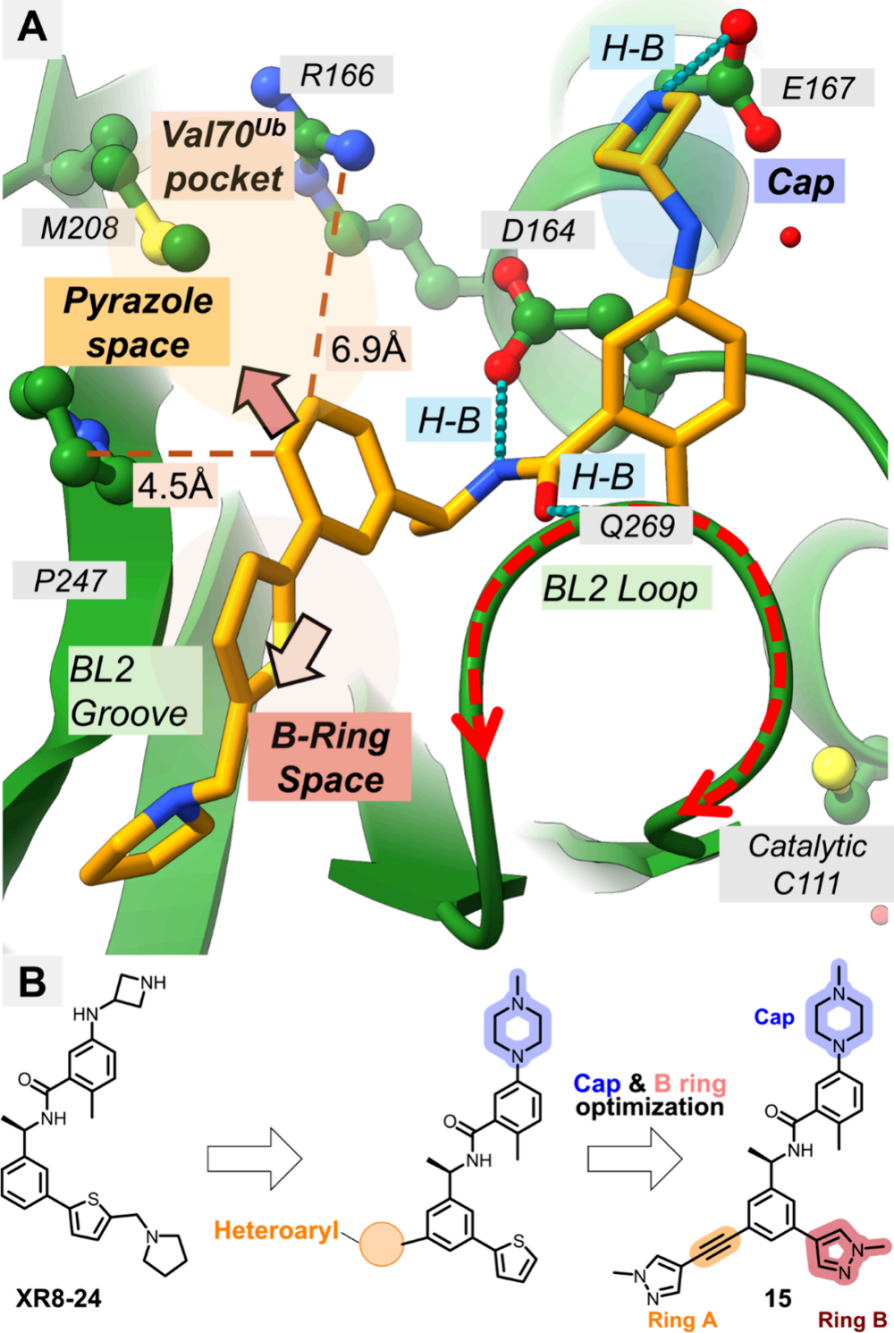
Structure-guided design of SARS-CoV-2 PLpro inhibitors. (A) Structural basis for optimization based on the PDB: 7LBS cocrystal structure with XR8–24. The structure highlights available space in the ″Cap″ region (blue) for engaging E167, the Val70^Ub^ pocket (Ring A, orange) accessible via the P247/R166 channel, and the Ring B space (scarlet). (B) Chemical evolution of XR8–24 to compound **15**. Optimization efforts focused on incorporating a piperazine moiety at the Cap (blue) to improve solvent interactions, extending a rigid alkyne-linker into the Val70Ub pocket (Ring A, orange) to capture hydrophobic interactions, and installing a pyrazole group (Ring B, scarlet) to fill the subpocket defined by the BL2 loop.

### Linker Optimization toward the Val70^Ub^ Pocket

Our initial objective was to identify a linker that would efficiently project a heteroaryl ring into the Val70^Ub^-binding pocket while maintaining productive interactions with the BL2 groove. To this end, we designed a focused series with Ring B and the Cap held constant and varied the linker geometry to incorporate methylene, amine, ether, and alkyne motifs, each terminating in a pyrazole A ring (Table 1). Relative to XR8–24, all linker variants produced a substantial enhancement in enzymatic potency, affording IC_50_ values in the 0.019–0.053 μM range, which corresponds to approximately an 11–22-fold improvement. These data indicate that each of the linkers is capable of positioning the pyrazole into the Val70^Ub^ pocket and strengthening overall PLpro engagement.

**Table 1:**
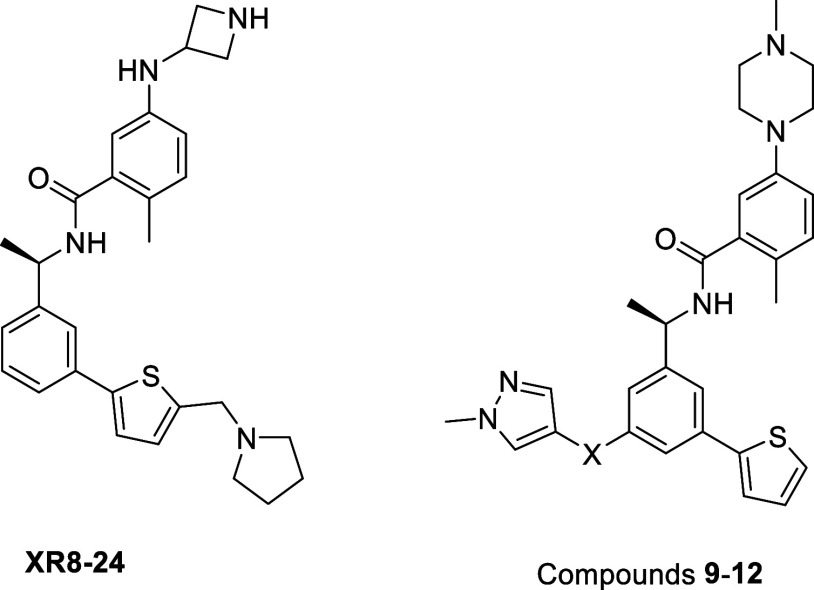

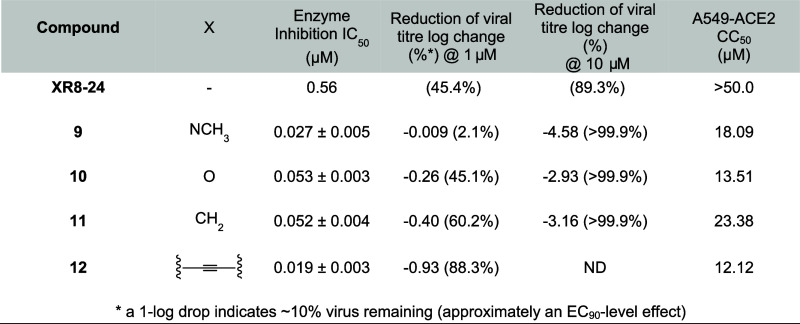
Biochemical Potency of Compounds **8**-**12**

In the cellular antiviral assay, an interesting divergence emerged. Among these analogs, the alkyne-linked compound **12** showed the most favorable profile, achieving a 0.93-log (88.3%) reduction in viral load at 1 μM. In contrast, the *N*-methyl linker, although the most potent in the biochemical assay, produced only marginal viral load reduction at 1 μM and required 10 μM to reach a 4.58-log decrease. These results highlight that relatively subtle changes in linker structure can maintain high enzymatic potency but have a strong influence on cellular antiviral activity.

### Ring A and Ring B Optimization

Compound **12**, the alkyne-linked pyrazole analog, combined strong enzymatic and cellular potency with engagement of the Val70^Ub^ pocket, but exhibited a relatively modest safety window, with a CC_50_ of 12.12 μM. To reduce the cytotoxicity while preserve potency, we next explored bioisosteric replacements of the thiophene B ring. Substitution of the thiophene with 1,3- or 1,4-pyrazole or with a dihydropyran B ring afforded a set of analogs that maintained low-nanomolar biochemical potency (IC_50_ < 0.04 μM) and showed encouraging improvement in CC_50_. For example, **13** and **15** displayed CC_50_ values of 36.35 and 34.54 μM, respectively, while retaining strong PLpro inhibition (Table 2). Notably, **14** and **15** also showed improved antiviral efficacy, producing 1.2- and 1.54-log reductions in viral load at 1 μM (93.7 and 97.1% reductions, respectively). Together, these data identify **15** as a well-balanced first-generation lead with robust enzymatic potency, strong cellular antiviral activity, and an improved cytotoxicity profile compared to **12**.

**Table 2:**
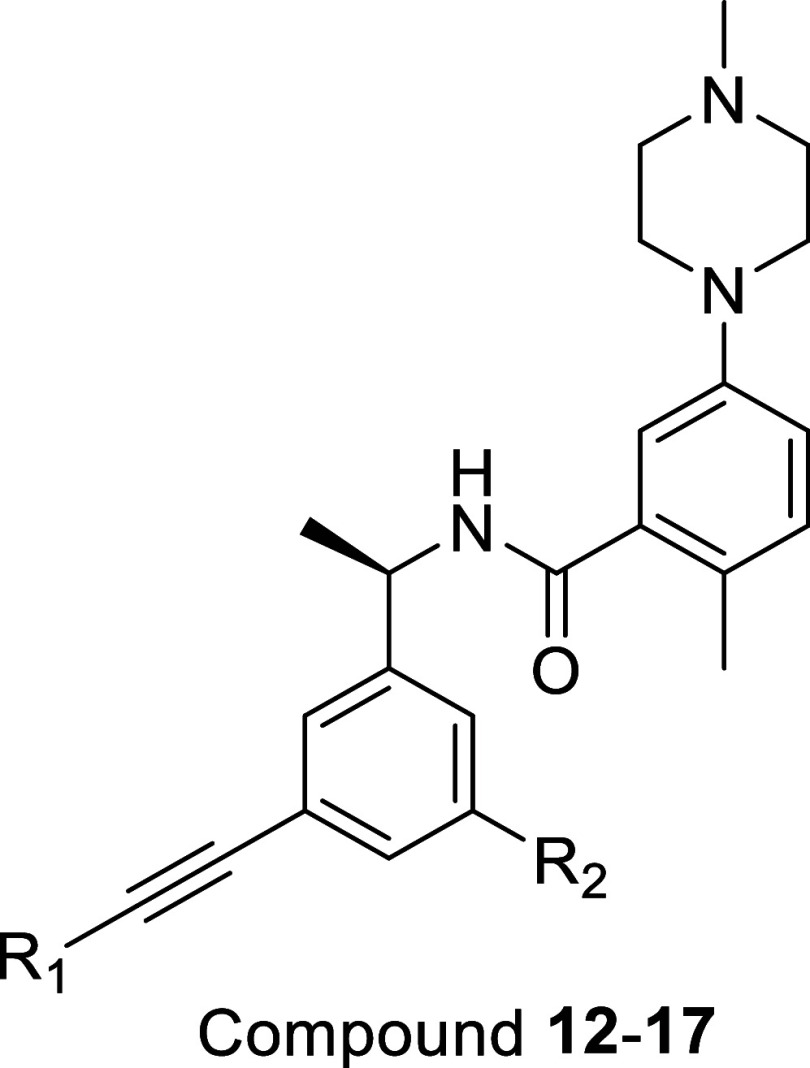

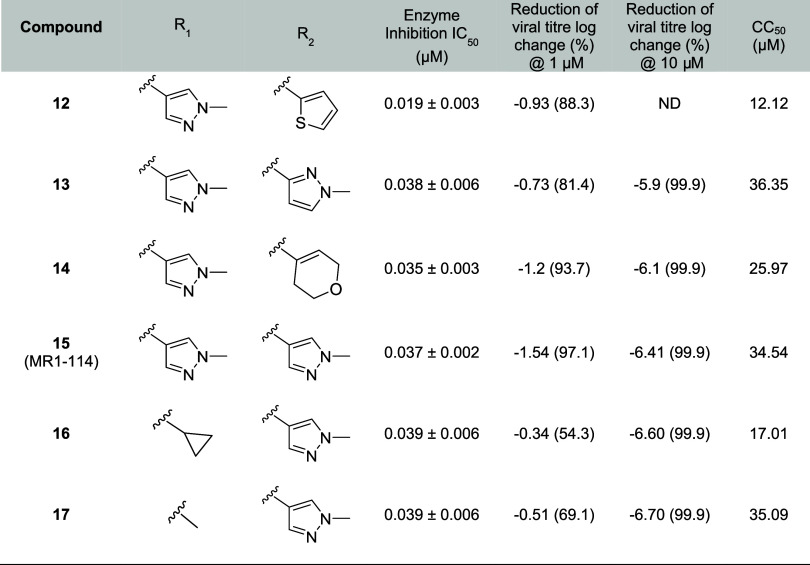
Biochemical Potency of Compounds **12**–**17**

With this more favorable baseline, we next examined modifications at Ring A, introducing either a methyl substituent (**17**) or a cyclopropyl (**16**) group. These analogs preserved potent enzymatic inhibition, with IC_50_ values around 0.039 μM, and achieved modest antiviral activity at 1 μM, with 0.51- and 0.34-log (69.1 and 54.3%) reductions in viral load (Table 2). At 10 μM, both A-ring variants produced more than a 6-log drop in viral load, comparable to **15**. Although these changes did not markedly surpass **15** in overall profile, they confirmed that the A-ring tolerates small hydrophobic substitutions without compromising the favorable potency and efficacy established in this series.

### Cap Optimization and Improvement of Human Liver Microsomal Stability

Given its attractive balance of enzymatic and antiviral activity and cytotoxicity, **15** was selected for further profiling in human liver microsomes. **15** exhibited a microsomal half-life of 90.9 min and an intrinsic clearance (CL_int_) of 15.5 μL per minute per mg protein (with NADPH and UDPGA, 1 μM substrate concentration) (Table 4). While this moderate clearance was acceptable, we aimed to further reduce CL_int_ below 10 μL per minute per mg protein to strengthen the overall pharmacokinetic profile. Internal metabolite identification studies on a related analog (data not shown) implicated the piperazine cap as a principal metabolic soft spot. We therefore explored bioisosteric replacements of the piperazine. Introduction of a (piperidin-4-yl)­oxy cap, as in **18**, resulted in weaker enzymatic and cellular potency and a narrower cytotoxicity window, and this analog was not pursued further (Table 3).

**Table 3:**
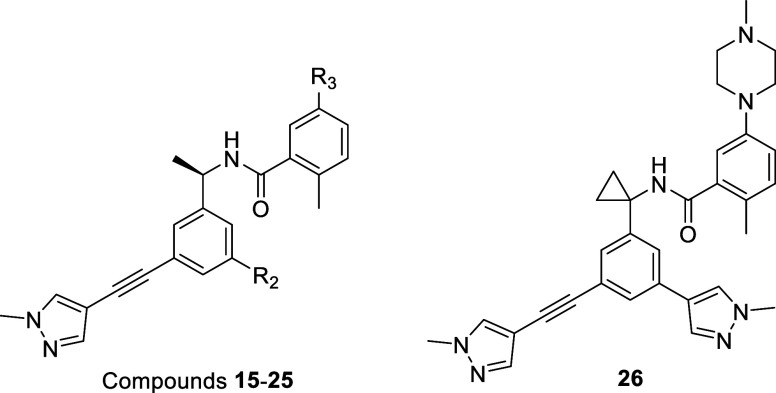

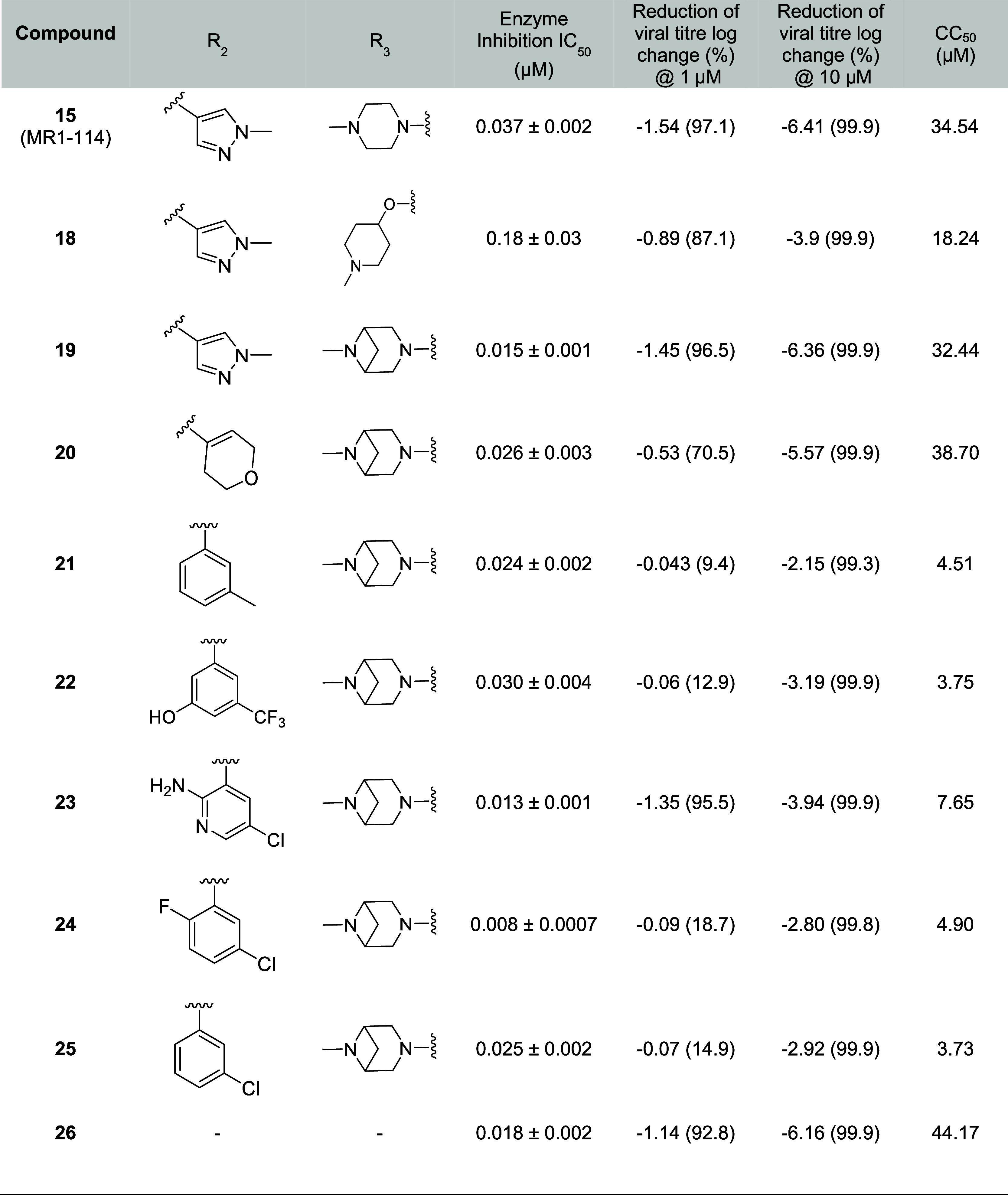
Biochemical Potency of Compounds **15** and **18**-**26**

In contrast, replacement of piperazine with a 3,6-diazabicyclo[3.1.1]­heptane cap afforded **19**, which showed a clear improvement across multiple activity parameters (Table 3). **19** demonstrated enhanced enzymatic potency with an IC_50_ of 0.015 μM and strong antiviral efficacy, achieving 1.45- and 6.36-log reductions in viral load at 1 and 10 μM, respectively. The cytotoxicity profile remained favorable, with a CC_50_ of 32.44 μM. Importantly, **19** exhibited markedly improved human liver microsomal stability, with a half-life greater than 184.8 min and a CL_int_ below 7.5 μL per minute per mg protein (Table 4). These data establish the 3,6-diazabicyclo[3.1.1]­heptane cap as a key optimization that improves both potency and metabolic stability while maintaining acceptable cytotoxicity. On this basis, **15** and **19** were both advanced to *in vivo* pharmacokinetic and efficacy studies to determine which profile would translate most favorably across species.

With the cap optimized, we revisited Ring B to determine whether further improvements in potency and antiviral activity could be achieved on the **19** scaffold. Keeping the 3,6-diazabicyclo[3.1.1]­heptane cap fixed, we introduced a panel of aryl and heteroaryl Ring B bioisosteres, including dihydropyran, substituted phenyl, and aminopyridine motifs (Table 3), guided by modeling (Figure 2A). Several analogs showed further gains in biochemical potency. For example, **24** displayed an approximately 4-fold improvement in PLpro inhibition relative to **15**, with an IC_50_ of 0.008 μM. However, in most cases these gains in enzymatic potency did not translate into superior antiviral activity, which may reflect cell-based determinants such as limited permeability/uptake, endosomal/lysosomal trapping, and reduced free compound levels due to serum or media binding.^46–48^ Many of the highly potent Ring B analogs exhibited reduced efficacy in the cellular viral load assay at both 1 and 10 μM. An exception was **20**, which incorporated a dihydropyran B ring and maintained strong antiviral efficacy while preserving a favorable overall profile. In contrast, **23**, bearing an aminopyridine B ring, combined potent PLpro inhibition (IC_50_ 0.013 μM) and good antiviral efficacy at 1 μM (1.35-log, 95.5% reduction) with a limited cytotoxicity window, as indicated by a CC_50_ of 7.65 μM. These results reinforce the importance of balancing enzymatic potency, cell-based antiviral activity, and cytotoxicity, and they highlight **20** as a valuable alternative B-ring design on the optimized cap scaffold.

In parallel with the cap and Ring B optimization, we also evaluated a chiral side-chain modification, replacing the R-methylamine substituent with a cyclopropyl group. This change afforded analog **26**, which exhibited approximately a 2-fold improvement in enzymatic potency relative to the parent methyl analog, along with slightly reduced viral load reduction at 1 μM and comparable antiviral activity at 10 μM. Encouragingly, **26** also displayed a modestly improved cytotoxicity profile, with a CC_50_ of 44.17 μM. These findings suggest that the cyclopropyl group is a useful variant at this position that can further fine-tune the potency and safety balance.

Integrating the biochemical inhibition data, cellular viral load reduction, cytotoxicity, and human liver microsomal stability, we initially nominated four compounds for advanced profiling: **15**, **19**, **20**, and **26**. **15** served as a well-balanced first-generation lead with strong enzymatic and cellular potency and a favorable safety window. **19** combined enhanced potency with significantly improved human liver microsomal stability and acceptable cytotoxicity. **20** provided an alternative Ring B design on the optimized cap scaffold that sustained robust antiviral efficacy, while **26** introduced a cyclopropyl side chain that further improved enzyme potency and cytotoxicity.

### Binding Model for MR1–114 (**15**)

To rationalize the molecular interactions of **15** with PLpro, we generated a docking pose using Schrödinger Glide and overlaid it with the binding pose of XR8–24 (Figure 3). As designed, the backbone amide-hydrogen bonds with D164 and Q269 are maintained, bending the BL2 loop. The pyrazole is oriented toward the BL2 groove, and the piperazine cap forms an electrostatic interaction with E167. Notably, the alkyne linker extends into the Val70^Ub^ pocket, mimicking ubiquitin’s hydrophobic residues Val70 and Leu71 during PLpro recognition, which likely contributes to the potency improvement of the alkyne-linked analogs.

**Figure 3:**
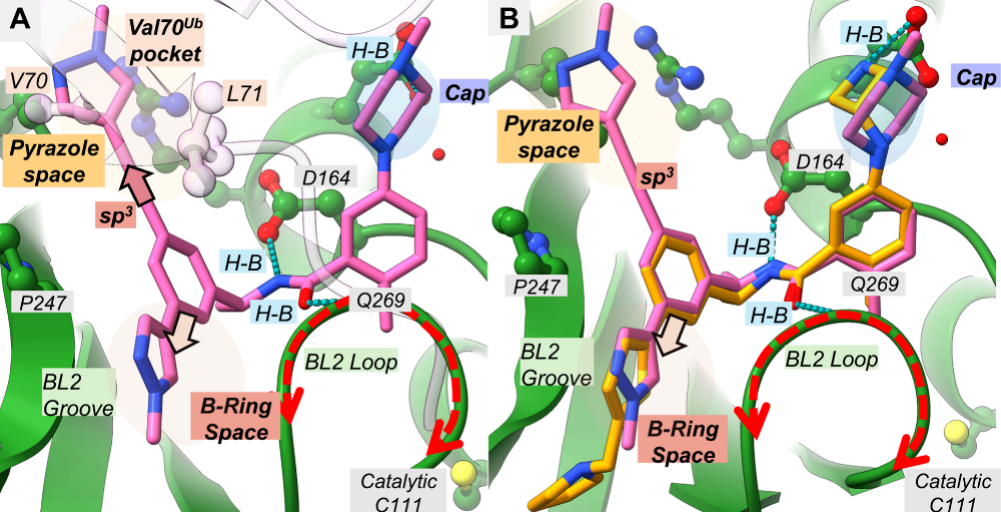
Overlay of **15** with cocrystal structure of SARS-CoV-2 PLpro with its Ubiquitin, and inhibitor XR8–24. (A) **15** (hot pink) docked structure overlaid with Ubiquitin-bound cocrystal structure of SARS-CoV-2 PLpro protease (light pink) (PDB ID: 6XAA). This aids to visualize where V70 is recognized by the cysteine protease. (B) Overlay structure of docked **15** (hot pink) with XR8–24 (orange) reveals occupation of pyrazole space. As aforementioned, the methylated pyrazole, near P247, points the methyl substitution down to the BL2 Groove. 7LBS (PDB), cocrystal structure, was used to perform docking and as a model of comparison with **15**.

### Physicochemical Properties

We profiled the physicochemical and ADME properties of the nominated compounds **15**, **19**, **20**, and **26**. The series demonstrated a favorable physicochemical baseline suitable for oral dosing. Kinetic solubility assays confirmed high aqueous solubility at physiological pH, with **15**, **20**, and **26** all soluble above 150 μM in PBS pH 7.4 (e.g., **15**: 152.6 μM; **20**: 234.8 μM, Table 4), indicating that dissolution is unlikely to limit absorption. Furthermore, all compounds exhibited excellent stability in human whole blood (*t*_1/2_ ≈ 400–500 min, Table S1), ruling out degradation in the systemic circulation as a differentiating factor.

**Table 4:** Physicochemical and ADME Properties of Selected Compounds^c^

compound	solubility (μM)	HLMS (CL_int_ (μL/min/mg protein)))	HLM *t*_1/2_ (min)	HS (CL_int_ (μL/min/10^6^ cells)	HS *t*_1/2_ (min)	% UB^a^	permeability (P_app_)	efflux ratio (ER)
**15**	152.6	15.5 ± 1.9	90.9 ± 11.2	7.97	174.45	0.9	1.2	19.1
**19**	NA	<7.50	>184.8	NA	NA	NA	NA	NA
**20**	234.8	9.1 ± 1.0	152.8 ± 16.9	2.36	600.51	3.6	0.1	158.0
**26**	185.8	19.6	70.6	5.3 ± 0.6	265.9 ± 29.3	1.2	0.5	51.8
diclofenac	NA	257.2 ± 1.4	5.4 ± 0.1	NA	NA	NA	NA	NA
verapamil	NA	270.5 ± 12.2	5.1 ± 0.2	58.7 ± 0.9	23.6 ± 0.4	NA	NA	NA
atenolol	NA	NA	NA	NA	NA	NA	0.6	1.5
digoxin	NA	NA	NA	NA	NA	NA	0.3	58.0
minoxidil	NA	NA	NA	NA	NA	NA	8.2	1.2
ketoconazole^b^	NA	NA	NA	NA	NA	0.7	NA	NA

a Protein binding in mouse plasma @ 5 μM Concentration. UB-Unbound.

b 1 μM Concentration.

c HLM—human liver microsomal. HLMS—human liver microsomal stability. HS—hepatocyte stability.

### Hepatocytes Stability and Permeability

A critical divergence emerged when comparing metabolic clearance with intestinal permeability. In human liver microsomes, **19** and **20** successfully met the design goal of attenuating oxidative metabolism, exhibiting intrinsic clearance values <10 μL/min/mg protein compared with the moderate stability of **15** (CL_int_ 15.5 μL/min/mg, Table 4). However, when evaluated in human hepatocytes, a more physiologically relevant system, these differences narrowed. **15** displayed a low intrinsic clearance of 7.97 μL/min/10^6^ cells, placing it in the same highly stable category as **26** and **20**. This confirms that the “moderate” microsomal clearance of **15** does not translate to rapid turnover in a whole-cell system.

Crucially, the structural modifications required to improve the metabolic stability of **19** and **20** negatively impacted membrane transport. In Caco-2 assays, **20** showed negligible absorptive permeability (P_app (A‑B)_ = 0.1 × 10^–6^ cm/s) and a high efflux ratio of 158.0. In contrast, **15** exhibited the most balanced profile, with an absorptive permeability of 1.2 × 10^–6^ cm/s, approximately 2-fold greater than the low-permeability control Atenolol. Despite being an efflux substrate (ER = 19.1), its intrinsic high permeability and good aqueous solubility are sufficient to support favorable oral absorption.

### *In Vivo* Pharmacokinetics, Tissue Distribution, and Safety Profile

To efficiently differentiate the metabolic stability and absorption potential of the series *in vivo*, we evaluated the candidates in a cassette pharmacokinetic study in C57BL/6J mice (10 mg/kg PO) (Table 5 and Figure 4A). Despite their improved *in vitro* microsomal stability, the bicyclic-amine analogs (**19** and **20**) exhibited limited systemic exposure (AUC 2251–4956 h·ng/mL), highlighting a key limitation of projecting *in vivo* PK from single *in vitro* assays. In this case, the lower exposure is more consistent with the *in vitro* permeability data, suggesting that absorption and/or other disposition processes (e.g., efflux, solubility/dissolution, plasma protein binding, or extra-hepatic clearance) can dominate overall exposure even when microsomal stability is favorable. **26** displayed high total exposure (AUC 8322 h·ng/mL) but suffered from extensive protein binding (98.8%, Table 4), reducing its effective free concentration. In this screening format, **15** emerged as the top candidate, displaying a robust profile with a superior balance of total exposure and unbound fraction (Table 4). On the basis of this head-to-head comparison, **15** was advanced for definitive single-agent profiling.

**Table 5:** PK Parameters of Selected Compounds^a^

	15	19	20	26
*T*_1/2_ (h)	NA	1.95 ± NA	2.33 ± NA	1.98 ± NA
*T*_max_ (h)	2.33 ± 1.53	3.33 ± 1.15	2.67 ± 1.15	2.67 ± 1.15
*C*_max_ (ng/mL)	1257 ± 155	464 ± 91	981 ± 191	1777 ± 492
AUC_last_ (h·ng/mL)	7487 ± 379	2251 ± 515	4956 ± 1315	8322 ± 2181

a Plasma pharmacokinetic profile in C57BL/6J mouse (10 mg/kg)—PO dose.

**Figure 4:**
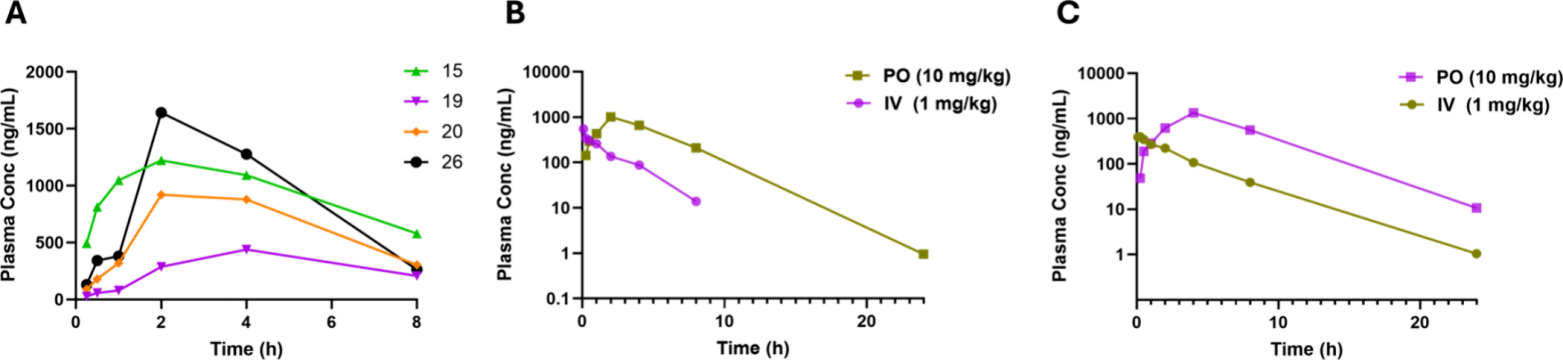
(A) Plasma concentration vs time profiles of compounds **15**, **19**, **20**, and **26** after 10 mg/kg, PO in C57BL6Mouse; (B) Plasma concentration vs time profile of compound **15** in C57BL6Mouse; (C) Plasma concentration vs time profile of compound **15** in SD rats.

To confirm the screening results and determine absolute oral bioavailability (F), **15** was evaluated in a discrete (single-compound) study in mice via both intravenous (1 mg/kg) and oral (10 mg/kg) administration in both mice and rats (Table 6 and Figure 4B,C). Following *IV* administration, **15** exhibited a moderate plasma clearance (CL_obs_ = 16.7 mL/min/kg) and a volume of distribution (V_ss_) of 2.33 L/kg, indicating extensive distribution into tissues. Upon oral dosing, the compound was rapidly absorbed (*T*_max_ ≈ 2.0 h) and achieved high systemic exposure (AUC_0‑infty_ = 6091 h·ng/mL), confirming the results from the cassette study. Importantly, comparison of the AUC values revealed a favorable oral bioavailability of 60.2%. This robust PK profile translated well to rats, where **15** showed lower plasma clearance (11.0 mL/min/kg) and even greater systemic exposure (AUC_0‑infty_ = 10954 h·ng/mL after oral dosing). Notably, **15** demonstrated excellent oral bioavailability in the rat (F = 71.2%), further supporting its potential for oral administration.

**Table 6:** PK Parameters for **15**

	p.o. mouse^a^	i.v. mouse^b^	p.o. rat^a^	i.v. rat^b^
*T*_1/2_(h)	2.11 ± 0.1	1.80 ± 0.3	2.78 ± 0.2	3.00 ± 0.2
plasma C_0_ or *C*_max_ (ng/mL)	1019 ± 182	704 ± 202	1339 ± 475	391 ± 47
plasma C_@_ 4h (ng/mL)	658 ± 86	88 ± 40	1339 ± 475	107 ± 17
plasma AUC_inf_ (h*ng/mL)	6091 ± 928	1012 ± 134	10954 ± 3075	1538 ± 218
bioavailability (F, %)	60.2 ± 9.2	NA	71.2 ± 20.0	NA
plasma Cl_obs_ (mL/min/kg)	NA	16.7 ± 2.2	NA	11.0 ± 1.4
V_ss__obs (L/kg)	NA	2.33 ± 0.31	NA	2.40 ± 0.13
lung/plasma conc ratio	6.03 ± 1.5^c^	NA	NA	NA
brain/plasma conc ratio	0.025 ± 0.005^c^	NA	NA	NA

a 10 mg/kg.

b 1 mg/kg.

c ratio @ 1h.

Given the respiratory pathology of SARS-CoV-2, we profiled the tissue distribution of **15** to assess its accumulation in the lung (10 mg/kg PO, 1 h postdose, Table 6 and Table S2). **15** exhibited preferential accumulation in the lung, reaching a mean concentration of 1587 ng/g. This corresponds to a lung-to-plasma ratio of 6.03, ensuring robust drug levels at the primary site of infection and consistent with our prior findings to XR8–23.^27^

Conversely, **15** displayed minimal penetration into the brain, with a mean concentration of only 6.63 ng/g and a brain-to-plasma ratio of 0.0252 (Table 6 and Table S2). This peripherally restricted profile is critical for mitigating safety risks, as our safety panel, which demonstrated a generally favorable profile, identified only a few CNS-related off-targets (>50% inhibition at 10 μM), most notably the 5-HT1A and Dopamine receptors (Table S3). The lack of CNS exposure effectively prevents the engagement of these receptors *in vivo.* Additional profiling showed moderate hERG inhibition (55.81% at 10 μM), which was consistent with a manageable off-target profile. Taken together, the high oral exposure, preferential lung targeting, and manageable safety profile position **15** as our primary lead.

### Antiviral Activity against SARS-CoV-2 Variants

Building on the favorable pharmacokinetic and safety profiles of our most advanced derivatives, we next sought to establish their broad-spectrum potential. To this end, the primary lead **15** and the cyclopropyl-amine analog **26** were selected for evaluation against a panel of major SARS-CoV-2 Variants of Concern (VOCs). Testing was conducted in A549-hACE2 cells to minimize efflux liabilities often observed in standard Vero lines. We first examined activity against the highly pathogenic Delta variant. Both compounds maintained robust submicromolar potency, with **15** displaying an EC_50_ of 0.18 μM and **26** showing an EC_50_ of 0.39 μM (Figure 5A,D). This confirms that the structural modifications in the lead series do not compromise activity against pre-Omicron strains. Given the dominance of Omicron sublineages in postpandemic circulation, we expanded the profiling to include Omicron B.1.1.529 and the immune-evasive Omicron BA.5 subvariant. Against Omicron B.1.1.529, **15** retained strong potency with an EC_50_ of 0.39 μM, while **26** showed slightly reduced activity (EC_50_ = 0.82 μM) (Figure 5B,D). Importantly, activity was fully preserved against the Omicron BA.5 variant, where **15** and **26** achieved EC_50_ values of 0.20 μM and 0.40 μM, respectively (Figure 5C,D). Collectively, these data demonstrate that **15** functions as a broad-spectrum inhibitor, maintaining varying degrees of submicromolar potency across diverse SARS-CoV-2 lineages without significant loss of activity due to viral evolution.

**Figure 5:**
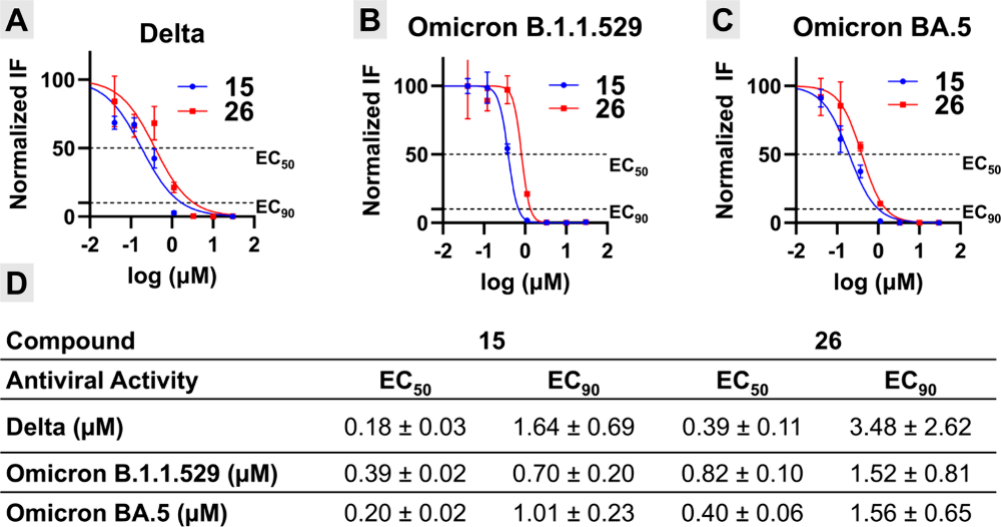
Broad-spectrum antiviral activity of **15**, and **26**, against SARS-CoV-2 variants of concern. Concentration–response curves were determined in A549-hACE2 cells infected with (A) Delta, (B) Omicron B.1.1.529, or (C) Omicron BA.5 variants. Cells were treated with increasing concentrations of **15** (blue circles) or **26** (red squares). Immunofluorescence data is plotted as normalized FI (Fluorescence Intensity) antispike protein normalized with DAPI. Data points represent the mean ± SE normalized to vehicle controls. (D) The calculated EC_50_ (μM) and EC_90_ (μM) are provided in the tables corresponding to each variant.

### Preclinical Efficacy in Mouse Models

Guided by its favorable lung-to-plasma ratio (∼6-fold) and overall strong cellular, ADME, and PK properties, we evaluated the therapeutic efficacy of **15** in a murine model of SARS-CoV-2 infection. K18-ACE2 mice were anesthetized via isoflurane inhalation and challenged intranasally with 20,000 PFU of the SARS-CoV-2 WA1 isolate (n = 8–10 per group). To mimic a therapeutic intervention, oral treatment started 1 h postinfection (Day 1) and continued twice daily (BID) until Day 5. The study compared **15** (100 or 150 mg/kg) against a vehicle control and the clinically approved Mpro inhibitor nirmatrelvir (1000 mg/kg, BID) as a positive benchmark. All treatments were formulated as oral suspensions containing 0.5% methylcellulose and 2% Tween-80. Efficacy was first assessed by monitoring body weight changes daily as a proxy for disease severity and general tolerability. Vehicle-treated animals developed measurable signs of disease, with progressive body-weight loss beginning on Day 2 (Figure 6A). In contrast, **15** treatment attenuated disease-associated weight loss: Animals in both the 100 mg/kg and 150 mg/kg cohorts maintained body-weight percentages comparable to the mock-infected and nirmatrelvir-treated groups. The absence of weight loss in the high-dose group also suggests that **15** is well tolerated at efficacious doses.

**Figure 6:**
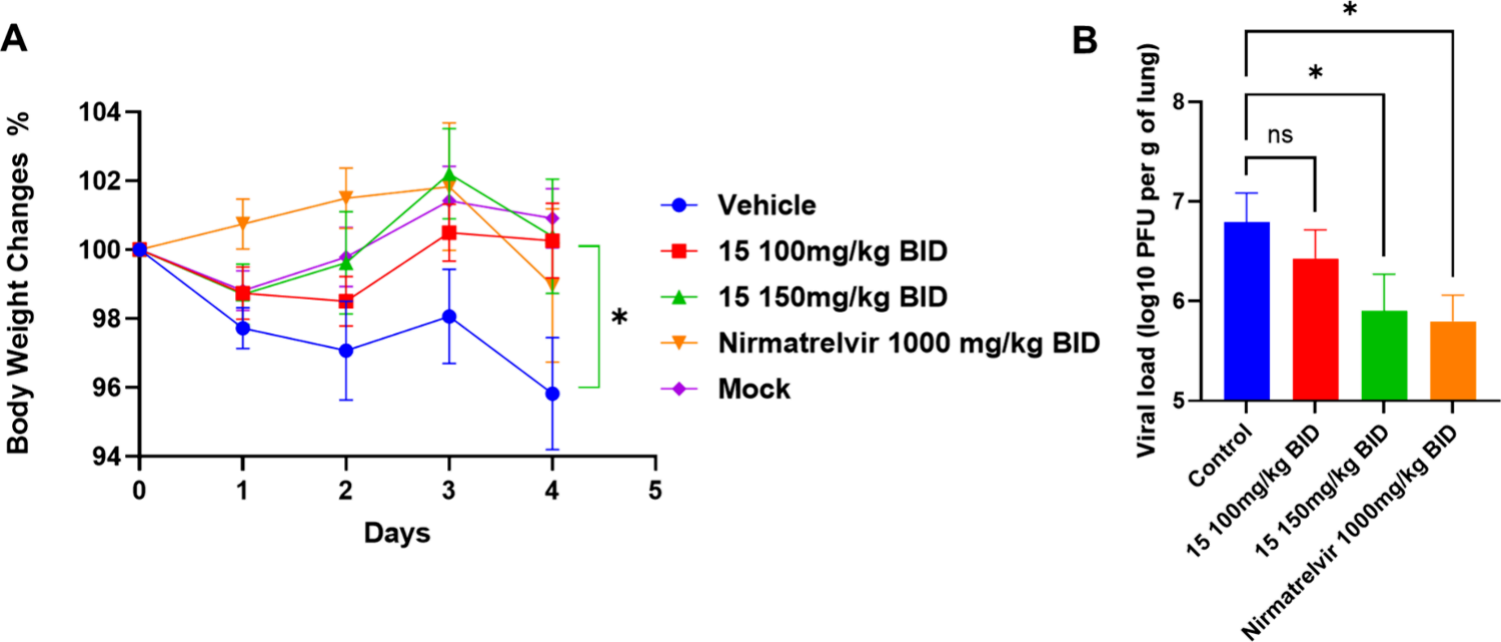
*In vivo* antiviral efficacy of **15** in a murine model of SARS-CoV-2 infection. (A) Percent body weight change over the 5-day treatment period. K18-hACE2 mice (*n* = 8–10) were infected intranasally with SARS-CoV-2 (WA1) and treated orally twice daily (BID) starting 1 h postinfection. Data are presented as mean ± SEM. Statistical significance for body weight loss was determined using the Mann–Whitney test comparing the vehicle control (0.5% methylcellulose, 2% Tween-80) to treatment groups (*P < 0.05). (B) Lung viral titers (PFU per gram of tissue) measured on Day 5. Infectious viral loads were quantified by plaque assay. Data represent mean ± SEM. Statistical significance was determined using a two-sided Kruskal–Wallis test with Dunn’s multiple comparison posthoc analysis (*P < 0.05; ns = not significant). **15** (150 mg/kg) significantly reduced lung viral titers comparable to the positive control nirmatrelvir.

To quantify the antiviral effect, infectious viral loads in the lungs were measured by plaque assay on Day 5 (1 h following the final dose). Treatment with **15** resulted in a dose-dependent suppression of viral replication (Figure 6B). While the 100 mg/kg dose showed a trend toward reduction, the 150 mg/kg regimen yielded a statistically significant decrease in lung viral titers compared to the vehicle control. Importantly, the magnitude of viral load reduction (∼1 log) observed with 150 mg/kg of **15** was comparable to that achieved by nirmatrelvir (1000 mg/kg, BID). Collectively, these data provide *in vivo* proof-of-concept for the selective PLpro inhibitor **15**, confirming its ability to prevent disease progression and significantly suppress pulmonary viral replication *via* oral administration.

### Chemistry

The synthesis of the **PLpro** inhibitors described herein generally followed the routes previously developed and optimized.^28,30,49^ The convergent synthesis of PLpro primarily utilized Ellman amine synthesis, reductive amination, amine coupling, and Suzuki–Miyaura cross-coupling and Sonogashira coupling reactions. For example, the synthesis of pyrazole-linked-derivatives **9–11** (Scheme 1) commenced with the preparation of the key synthon **S3**, which was obtained by Suzuki–Miyaura cross-coupling of dibromo ketone then Ellman amine synthesis. The protected amine **S3** then underwent respective cross-coupling reactions followed by deprotection in DCM, and a final amide coupling to yield the desired compounds **9**-**11.**

**Scheme 1:**
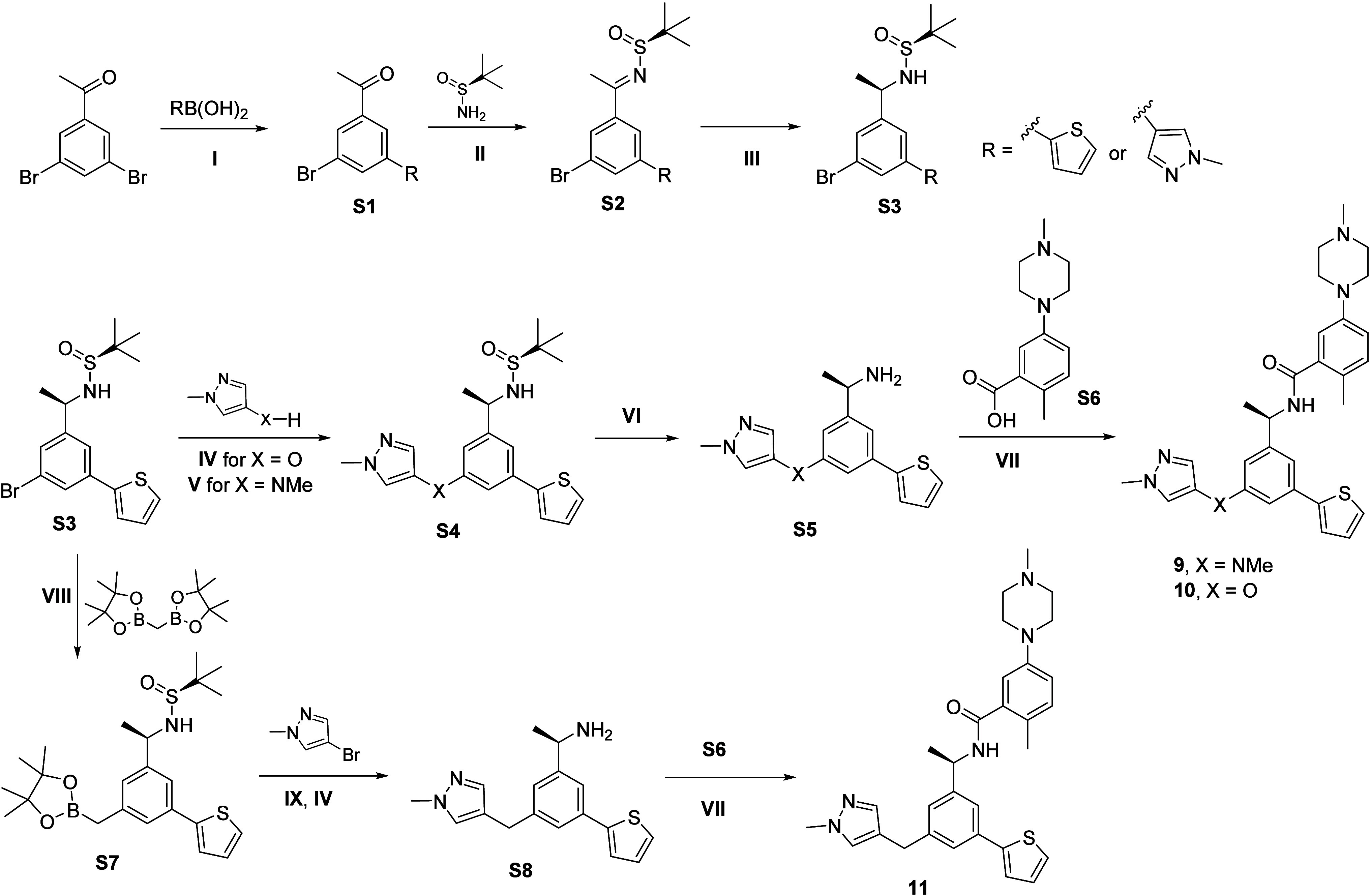
Synthesis of **9**–**11** ^a^

a Reagents and conditions: (I) Pd­(PPh_3_)_4_, Na_2_CO_3_, toluene/EtOH/H_2_O, 90 °C, 12 h; (II) Ti­(OEt)_4_, THF, 25-70 °C, 14 h; (III) l-Selectride, THF, −70 °C, 30 min; (IV) *N*,*N*-dimethylglycine hydrochloride, CuI, Cs_2_CO_3_, 120 °C, 12 h; (V) RuPhos Pd G4, Cs_2_CO_3_, dioxane, 100 °C, 16 h; (VI) HCl/dioxane, dioxane, 25 °C, 2 h; (VII) HATU, DIPEA, DMF, 25 °C, 1 h; (VIII) cataCXium A Pd G3, Cs_2_CO_3_, H_2_O/dioxane, 80 °C, 1 h; (IX) Pd­(OAc)_2_, K_3_PO_4_, SPhos, dioxane/H_2_O, 60 °C, 3 h.

Compounds **12**–**15** were synthesized from **S18** by Suzuki–Miyaura coupling with the corresponding pyrazole, thiophene, and pyran boronic esters using XPhos Pd G2 as the catalyst. The synthon **S18** was obtained from chiral amine intermediate **S11**, which was coupled with acid **S6** followed by a Sonogashira coupling reaction with pyrazole-alkyne. The chiral amine intermediate **S11** was synthesized via an Ellman asymmetric amine synthesis starting from iodo-bromo-acetophenone (Scheme 2).

**Scheme 2:**
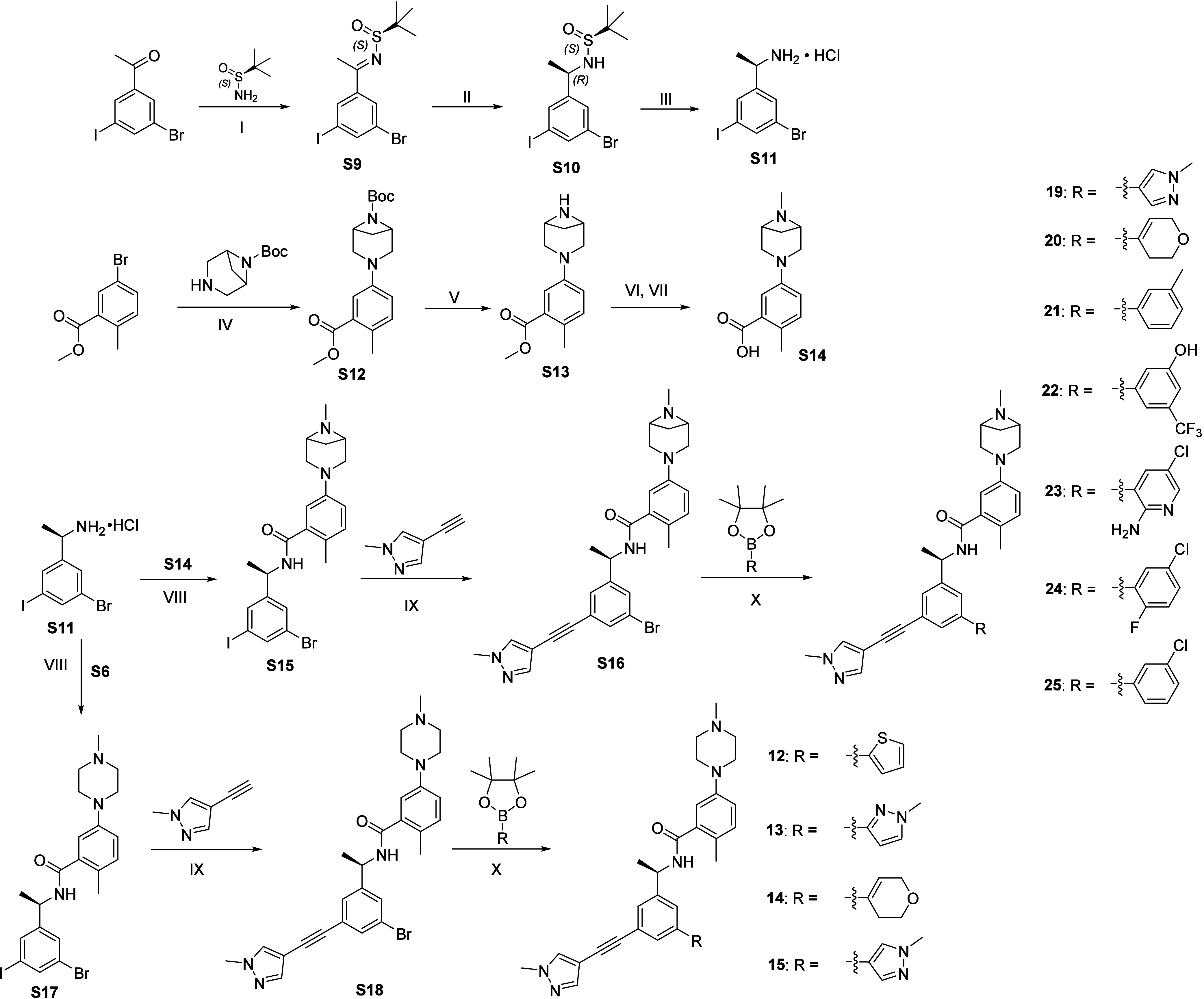
Synthesis of **12**–**15** and **19**–**25** ^a^

a Reagents and conditions: (I) Ti­(OEt)_4_, THF, 25-70 °C, 14 h; (II) l-Selectride, THF, −70-25 °C, 14 h; (III) HCl/dioxane, dioxane, 25 °C, 4 h; (IV) RuPhos Pd G3, Cs_2_CO_3_, dioxane, 100 °C, 6 h; (V) TFA, DCM, r.t., 1-2 h; (VI) formaldehyde, formic acid, NaBH­(OAc)_3_, MeOH, r.t., 1 h; (VII) LiOH.H_2_O, THF, MeOH, Water, r.t., 12 h; (VIII) HATU, TEA, DMAP, DMF, r.t., 1 h; (IX) Pd­(PPh_3_)_2_Cl_2_, CuI, TEA, THF, rt, 30 min; (X) XPhos Pd G2, Cs_2_CO_3_, dioxane/water (3:1), Ar atm., 90 °C, 1 h.

Compounds **16** and **17** were synthesized from **S20**, which was obtained by deprotection of synthon **S3** followed by amide coupling reaction with **S6** (Scheme 3). The synthon **S20** was then subjected to Sonogashira coupling reaction^50^ with respective alkynes to afford the desired final compounds **16** and **17**. Similarly, compound **18** was synthesized from Sonogashira coupling reaction of **S24** with pyrazole-alkyne. Intermediate **S24** was previously obtained from the coupling of amine **S19** and acid **S23**. The acid synthon **S23** was synthesized from methyl 5-hydroxy-2-methylbenzoate via consecutive Mitsunobu reaction, deprotection and reductive amination reactions (Scheme 3).

**Scheme 3:**
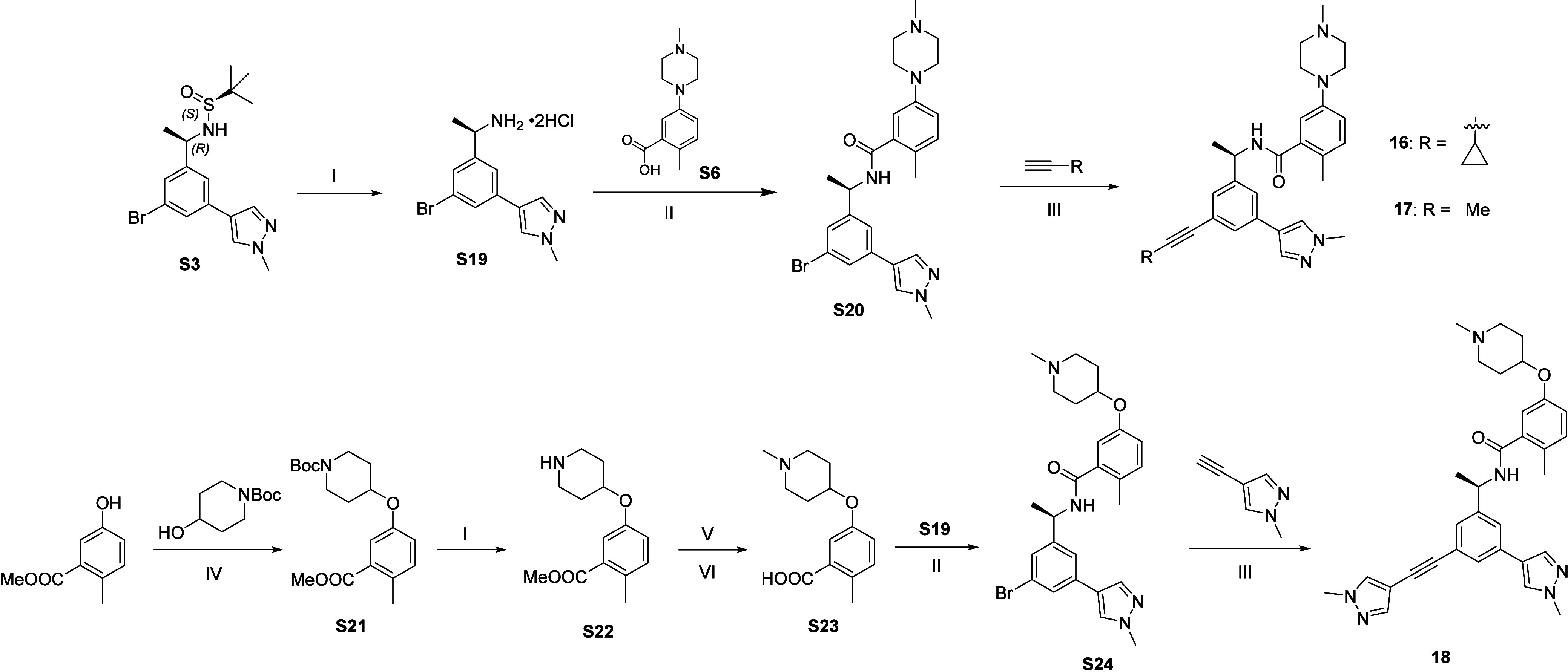
Synthesis of **16**–**18**

a Reagents and conditions: (I) HCl/dioxane, dioxane, 25 °C, 2 h; (II) HATU, TEA, DMAP, DMF, r.t., 1 h; (III) [Pd­(CH_3_CN)_2_]­Cl_2_, Xphos, Cs_2_CO_3_, ACN, 85°C, 4 h; (IV) triphenylphosphine, DIAD, THF, r.t., 12 h; (V) formaldehyde, formic acid, NaBH­(OAc)_3_, MeOH, r.t., 1 h; (VI) LiOH·H_2_O, THF, MeOH, Water, r.t., 12 h.

Compounds **19**–**25** were synthesized from intermediate **S11** following a route analogous to that used for compounds **12**-**15**. Specifically, the chiral amine intermediate **S11** was coupled with acid synthon **S14** followed by Sonogashira coupling reaction with pyrazole-alkyne. Final Suzuki–Miyaura coupling with substituted phenyl or substituted heteroaromatic boronic esters/acids, using XPhos Pd G2 as the catalyst, afforded compounds **19**-**25** (Scheme 2). The acid synthon **S14** was obtained from bromo-ester via a sequence of Buchwald coupling, deprotection, and reductive-amination.

The synthetic route to prepare compound **26** was illustrated in Scheme 4. 3-bromo-5-iodobenzonitrile was subjected to a Sonogashira coupling reaction with pyrazole-alkyne, followed by Kulinkovich-Szymoniak reaction to obtain the cyclopropylamine **S26**.^49^ This intermediate was then coupled with acid **S6**, and a final Suzuki–Miyaura coupling with a pyrazole-boronic ester provided the desired compound **26.**

**Scheme 4:**
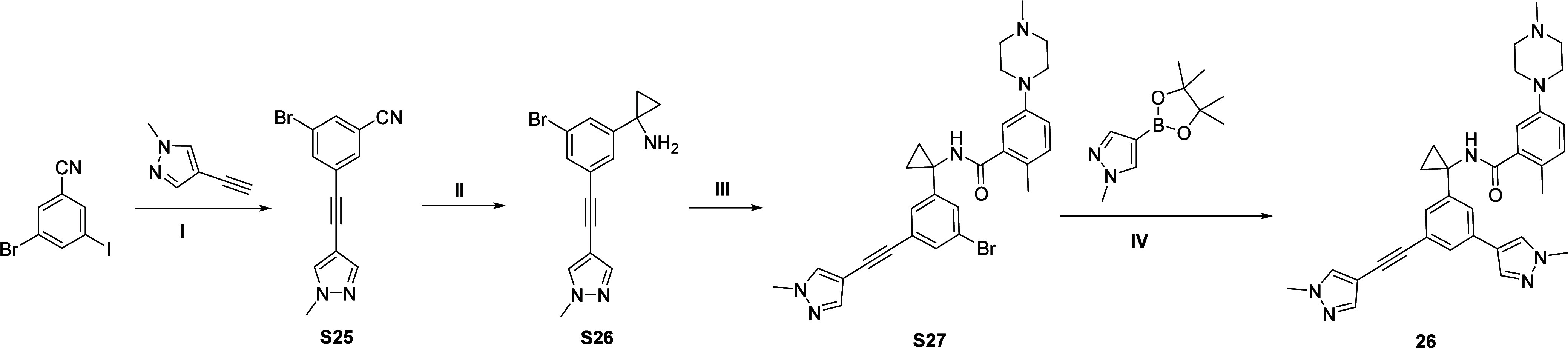
Synthesis of **26** ^a^

a Reagents and conditions: (I) Pd­(PPh_3_)_2_Cl_2_, CuI, TEA, THF, r.t., 30 min; (II) EtMgBr, Ti­(OEt)_4_, BF_3_.OEt_2_, −78 °C to r.t.; (III) **S6**, HATU, DMAP, TEA, DMF, r.t.; (IV) XPhos Pd G2, Cs_2_CO_3_, dioxane/water (3:1), Ar atm., 90 °C, 1 h.

## Conclusions

In summary, we have described the structure-based discovery of **15**, a potent, noncovalent inhibitor of the SARS-CoV-2 papain-like protease (PLpro). Rational design focused on dual engagement of the BL2 groove and an adjacent cryptic hydrophobic Val70Ub pocket proved critical for maximizing cellular potency while maintaining high biochemical affinity, with optimization of a rigid alkyne linker providing the optimal vector for this interaction. Multiparameter optimization culminated in **15**, which balances human hepatocyte metabolic stability with absorptive permeability to deliver robust oral bioavailability in mice (F ∼ 60%). Notably, **15** exhibits a favorable tissue-distribution profile for a respiratory therapeutic, characterized by pronounced lung enrichment (lung/plasma ratio ∼ 6 at 1 h) and minimal central nervous system penetration, thereby limiting potential off-target risks. This pharmacokinetic profile translates into therapeutic benefit: in the K18-hACE2 mouse model, oral administration of **15** produced antiviral efficacy comparable to the clinical standard nirmatrelvir, significantly suppressing pulmonary viral replication and preventing disease-associated morbidity. Collectively, these attributes establish **15** as a valuable *in vivo* lead and a promising starting point for the development of next-generation PLpro-targeted antivirals.

## Experimental Section

### Chemical Synthesis

Detailed methods are provided in the Supporting Information, including characterization and purity. Unless otherwise specified, reactions were performed under an inert atmosphere of argon and monitored by thin-layer chromatography (TLC) and/or LCMS. All reagents and solvents were purchased from commercial suppliers (Sigma-Aldrich, Fisher Scientific, Ambeed, Combi-Blocks, Enamine) and used as provided. Synthetic intermediates were purified using a CombiFlash chromatography system on 230–400 mesh silica gel or a Shimadzu prep-HPLC system. ^1^H and ^13^C NMR spectra were obtained using Bruker DPX-400 or AVANCE-400 spectrometer at 400 and 100 MHz, respectively. NMR chemical shifts were described in δ (ppm) using residual solvent peaks as standard. High-resolution mass spectral data were measured in-house using a Shimadzu IT-TOF LC/MS for all final compounds. Optical rotations were measured with a PerkinElmer 241 polarimeter operating on the mercury lamp line (546 nm), using a 100 mm path length cell. All compounds submitted for biochemical and biological testing were confirmed to be ≥ 95% pure by analytical HPLC.

### General Procedure A (Acid-Amine Coupling)

To a solution of acid (example: acid compound **S6** in Scheme 1, 1 equiv) and amine (example: amine compound **S5** or **S8** in Scheme 1, 1 equiv) in anhydrous DMF was added DIPEA (4 equiv), and the solution was cooled to 0 °C. HATU (1.2 equiv) was added to the solution under 0 °C and then stirred at rt for 1–4 h, progress of the reaction monitored by LCMS. The crude LCMS showed the starting material was consumed, and desired mass ion was observed. The mixture was diluted with methanol and purified by preparative HPLC (column: C18 50 × 30 mm; mobile phase: [Water (Formic acid)-Acetonitrile]; flow rate = 18 mL/min) afforded the desired amide compound.

### General Procedure B (Suzuki Coupling)

To a solution of halo-substituted intermediate (example: **S16** or **S18** in Scheme 2, 1 equiv) in 1,4-Dioxane/water (3/1) was added boronic acid or boronic ester (1.5 equiv) and cesium carbonate (2 equiv) at room temperature. The reaction mixture was purged with Ar for 15 min followed by addition of XPhos-Pd-G2 (0.2 equiv). The reaction mixture was stirred at 90 °C for 1 h. The reaction progress was monitored by LCMS. On completion the reaction mixture was quenched with water (5 mL) followed by extraction with DCM (3 × 5 mL). Combined organic phases were dried over Na_2_SO_4_, filtered and concentrated. Crude product was purified by preparative HPLC (column: C18 50 × 30 mm; mobile phase: [Water (Formic acid)-Acetonitrile]; flow rate = 18 mL/min) afforded the desired compound.

### General Procedure C (Sonogashira Coupling)

Following a known procedure with modifications,^50^ a flame-dried vial equipped with a magnetic stir bar was charged with bromo-intermediate (example: **S20** or **S24** in Scheme 3, 1 equiv), alkyne (2.5 equiv), PdCl_2_.(CH_3_CN)_2_ (5 mol %), XPhos (10 mol %), Cs_2_CO_3_ (2.5 equiv), and CH_3_CN (1 mL). The vial was heated at 85 °C for 4 h. Reaction mixture was quenched with water (5 mL) followed by extraction with DCM (3 × 5 mL). Combined organic phases were dried over Na_2_SO_4_, filtered and concentrated. Crude product was purified by preparative HPLC (column: C18 50 × 30 mm; mobile phase: [Water (Formic acid)-Acetonitrile]; flow rate = 18 mL/min) afforded desired alkyne compound.

#### (*R*)-2-Methyl-*N*-(1-(3-(methyl­(1-methyl-1*H*-pyrazol-4-yl)­amino)-5-(thiophen-2-yl)­phenyl) ethyl)-5-(4-methylpiperazin-1-yl)­benzamide (**9**)

Compound **9** was prepared as a white solid following general procedure **A** (41.2 mg, yield 78%). ^1^H NMR (400 MHz, DMSO-*D*6) δ 8.67 (d, *J* = 8.1 Hz, 1H), 7.71 (s, 1H), 7.54–7.48 (m, 1H), 7.42–7.35 (m, 2H), 7.15–7.06 (m, 2H), 7.06–7.01 (m, 1H), 6.99–6.79 (m, 4H), 5.08–4.96 (m, 1H), 3.81 (s, 3H), 3.33 (s, 3H), 3.21 (s, 3H), 3.11–2.79 (m, 4H), 2.59–2.55 (m, 4H), 2.18 (s, 3H), 1.40 (d, *J* = 7.0 Hz, 3H). ^13^C NMR (101 MHz, DMSO-*D*_6_) δ 168.44, 149.64, 146.81, 144.27, 137.80, 134.29, 134.10, 131.04, 130.71, 128.33, 125.35, 125.26, 123.44, 116.83, 114.51, 113.10, 111.14, 109.20, 48.49, 40.88, 39.00, 22.80, 18.40. LCMS: [M + H]^+^ 529.28.

#### (*R*)-2-Methyl-*N*-(1-(3-((1-methyl-1*H*-pyrazol-4-yl)­oxy)-5-(thiophen-2-yl)­phenyl)­ethyl)-5-(4-methylpiperazin-1-yl)­benzamide (**10**)

Compound **10** was prepared as a white solid following general procedure **A** (42.8 mg, yield 83%). ^1^H NMR (400 MHz, CHLOROFORM-*D*) δ 7.31 (s, 1H), 7.30–7.23 (m, 4H), 7.18–7.11 (m, 1H), 7.10–7.01 (m, 2H), 6.98–6.90 (m, 1H), 6.90–6.78 (m, 2H), 6.21 (d, *J* = 8.0 Hz, 1H), 5.29–5.17 (m, 1H), 3.86 (s, 3H), 3.37–3.30 (m, 4H), 3.01–2.94 (m, 4H), 2.62 (s, 3H), 2.29 (s, 3H), 1.56 (d, *J* = 7.0 Hz, 3H). ^13^C NMR (101 MHz, CHLOROFORM-*D*) δ 169.47, 159.89, 148.20, 146.03, 143.61, 140.18, 137.26, 136.42, 132.04, 131.10, 128.21, 127.92, 125.46, 123.93, 121.08, 118.52, 118.17, 115.41, 112.94, 112.59, 54.17, 49.31, 47.91, 44.55, 39.90, 22.18, 18.94.

#### (*R*)-2-Methyl-*N*-(1-(3-((1-methyl-1*H*-pyrazol-4-yl)­methyl)-5-(thiophen-2-yl)­phenyl) ethyl)-5-(4-methylpiperazin-1-yl)­benzamide (**11**)

Compound **11** was prepared as a white solid following general procedure **A** (38.0 mg, yield 74%). ^1^H NMR (400 MHz, CHLOROFORM-*D*) δ 8.21 (s, 1H), 7.44–7.40 (m, 1H), 7.33–7.29 (m, 1H), 7.25–7.21 (m, 2H), 7.12–7.07 (m, 2H), 7.06–6.96 (m, 2H), 6.84–6.79 (m, 1H), 6.79–6.72 (m, 1H), 6.47 (d, *J* = 7.9 Hz, 1H), 5.24–5.16 (m, 1H), 3.79–3.74 (m, 5H), 3.31–3.22 (m, 4H), 3.13–3.07 (m, 4H), 2.66 (s, 3H), 2.24 (s, 3H), 1.55 (d, *J* = 6.9 Hz, 3H). ^13^C NMR (101 MHz, CHLOROFORM-*D*) δ 165.90, 147.72, 144.42, 144.15, 142.50, 138.87, 137.30, 135.07, 132.05, 129.24, 128.39, 128.21, 125.74, 125.28, 125.12, 123.53, 121.77, 120.49, 118.78, 115.58, 53.45, 49.42, 47.19, 43.53, 38.87, 30.64, 22.07, 18.90. LCMS: [M + H]^+^ 514.71.

#### (*R*)-2-Methyl-*N*-(1-(3-((1-methyl-1*H*-pyrazol-4-yl)­ethynyl)-5-(thiophen-2-yl)­phenyl)­ethyl)-5-(4-methylpiperazin-1-yl)­benzamide (**12**)

Compound **12** was prepared as a white solid following general procedure **B** (30.5 mg, 86%). ^1^H NMR (400 MHz, METHANOL-*D*_4_) δ 7.86 (s, 1H), 7.69–7.60 (m, 3H), 7.49–7.39 (m, 3H), 7.16–7.08 (m, 2H), 7.00–6.92 (m, 2H), 5.21 (q, *J* = 7.0 Hz, 1H), 3.91 (s, 3H), 3.25–3.18 (m, 4H), 2.73–2.66 (m, 4H), 2.41 (s, 3H), 2.27 (s, 3H), 1.56 (d, *J* = 7.1 Hz, 3H). ^13^C NMR (101 MHz, METHANOL-*D*_4_) δ 172.68, 150.25, 146.97, 144.40, 142.70, 138.47, 136.38, 134.85, 132.47, 129.31, 128.99, 127.91, 127.85, 126.46, 125.83, 124.93, 124.26, 119.07, 116.03, 104.40, 90.30, 81.89, 55.77, 50.27, 49.83, 45.74, 39.10, 22.25, 18.68.

#### (*R*)-2-Methyl-*N*-(1-(3-(1-methyl-1*H*-pyrazol-3-yl)-5-((1-methyl-1*H*-pyrazol-4-yl)­ethynyl) phenyl)­ethyl)-5-(4-methylpiperazin-1-yl)­benzamide (**13**)

Compound **13** was prepared as a white solid following general procedure **B** (29 mg, yield 83%). ^1^H NMR (400 MHz, METHANOL-*D*_4_) δ 7.78–7.73 (m, 3H), 7.63–7.54 (m, 2H), 7.44 (d, *J* = 1.8 Hz, 1H), 7.16–7.10 (m, 1H), 6.98–6.90 (m, 2H), 6.64–6.59 (m, 1H), 5.23 (q, *J* = 6.8 Hz, 1H), 3.94 (s, 3H), 3.91 (s, 3H), 3.38–3.33 (m, 4H), 3.22–3.18 (m, 4H), 2.78 (s, 3H), 2.27 (s, 3H), 1.58 (d, *J* = 7.1 Hz, 3H). ^13^C NMR (101 MHz, METHANOL-*D*4) δ 171.85, 151.76, 148.77, 145.72, 142.44, 138.31, 134.85, 134.38, 133.31, 132.41, 129.17, 128.80, 127.93, 125.11, 123.99, 119.21, 116.37, 103.99, 90.45, 81.26, 54.59, 50.09, 48.36, 43.98, 39.00, 22.07, 18.78.

#### (*R*)-*N*-(1-(3-(3,6-Dihydro-2*H*-pyran-4-yl)-5-((1-methyl-1*H*-pyrazol-4-yl)­ethynyl)­phenyl)­ethyl)-2-methyl-5-(4-methylpiperazin-1-yl)­benzamide (**14**)

Compound **14** was prepared as a white solid following general procedure **B** (28 mg, yield 78%). ^1^H NMR (400 MHz, METHANOL-*D*_4_) δ 7.84 (s, 1H), 7.63 (s, 1H), 7.49–7.44 (m, 1H), 7.45–7.35 (m, 2H), 7.15–7.07 (m, 1H), 7.00–6.90 (m, 2H), 6.28–6.19 (m, 1H), 5.18 (q, *J* = 7.1 Hz, 1H), 4.30 (q, *J* = 2.8 Hz, 2H), 3.97–3.87 (m, 5H), 3.20 (t, *J* = 5.0 Hz, 4H), 2.66 (t, *J* = 5.0 Hz, 4H), 2.59–2.45 (m, 2H), 2.38 (s, 3H), 2.25 (s, 3H), 1.53 (d, *J* = 7.0 Hz, 3H). ^13^C NMR (101 MHz, METHANOL-*D*_4_) δ 172.60, 150.33, 146.18, 142.63, 142.20, 138.49, 134.82, 134.74, 132.45, 128.83, 127.80, 127.25, 125.21, 124.42, 123.61, 119.03, 115.97, 104.53, 90.70, 81.32, 66.79, 65.48, 55.85, 50.40, 49.93, 45.89, 39.08, 28.20, 22.31, 18.65.

#### (*R*)-2-Methyl-*N*-(1-(3-(1-methyl-1*H*-pyrazol-4-yl)-5-((1-methyl-1*H*-pyrazol-4-yl)­ethynyl) phenyl)­ethyl)-5-(4-methylpiperazin-1-yl)­benzamide (**15**)

Compound **15** was prepared as a white solid following general procedure **B** (30 mg, yield 83%). ^1^H NMR (400 MHz, METHANOL-*D*_4_) δ 7.97 (s, 1H), 7.82 (s, 2H), 7.60 (s, 1H), 7.58–7.50 (m, 2H), 7.36–7.31 (m, 1H), 7.17–7.10 (m, 1H), 7.01–6.91 (m, 2H), 5.18 (q, *J* = 7.0 Hz, 1H), 3.91 (s, 3H), 3.88 (s, 3H), 3.37–3.32 (m, 4H), 3.26–3.19 (m, 4H), 2.80 (s, 3H), 2.24 (s, 3H), 1.54 (d, *J* = 7.0 Hz, 3H). ^13^C NMR (101 MHz, METHANOL-*D*4) δ 170.98, 147.96, 145.20, 141.30, 137.41, 136.14, 133.41, 133.16, 131.31, 128.14, 127.50, 126.57, 126.39, 124.33, 122.84, 122.32, 118.10, 115.18, 103.17, 89.31, 80.17, 53.46, 49.11, 47.17, 42.58, 37.72, 20.92, 17.37. HRMS (ESI) calcd for C_31_H_36_N_7_O [M + H]+, 522.29759; found, 522.29746.

#### (*R*)-*N*-(1-(3-(Cyclopropylethynyl)-5-(1-methyl-1*H*-pyrazol-4-yl)­phenyl)­ethyl)-2-methyl-5-(4-methylpiperazin-1-yl)­benzamide (**16**)

Compound **16** was prepared as an off-white solid following general procedure **C** (16.8 mg, yield 35%). ^1^H NMR (400 MHz, CHLOROFORM-*D*) δ 7.73 (s, 1H), 7.61 (s, 1H), 7.42–7.36 (m, 2H), 7.25–7.23 (m, 1H), 7.14–7.04 (m, 1H), 6.92–6.80 (m, 2H), 6.13 (d, *J* = 8.2 Hz, 1H), 5.33–5.21 (m, 1H), 3.92 (s, 3H), 3.50–3.32 (m, 4H), 3.17–2.99 (m, 4H), 2.68 (s, 3H), 2.32 (s, 3H), 1.61–1.57 (m, 3H), 1.51–1.39 (m, 1H), 0.91–0.85 (m, 2H), 0.84–0.78 (m, 2H). ^13^C NMR (101 MHz, CHLOROFORM-*D*) δ 169.23, 148.03, 143.88, 137.40, 136.85, 133.28, 132.09, 128.37, 127.84, 127.34, 127.22, 125.00, 123.12, 122.47, 118.79, 115.85, 94.10, 75.62, 53.66, 49.18, 47.67, 44.01, 39.27, 21.85, 18.98, 8.81.

#### (*R*)-2-Methyl-*N*-(1-(3-(1-methyl-1*H*-pyrazol-4-yl)-5-(prop-1-yn-1-yl)­phenyl)­ethyl)-5-(4-methylpiperazin-1-yl)­benzamide (**17**)

Compound **17** was prepared as an off-white solid following general procedure **C** (17.3 mg, yield 38%). ^1^H NMR (400 MHz, CHLOROFORM-*D*) δ 7.72 (s, 1H), 7.60 (s, 1H), 7.43–7.37 (m, 2H), 7.28–7.23 (m, 1H), 7.12–7.03 (m, 1H), 6.93–6.88 (m, 1H), 6.88–6.82 (m, 1H), 6.10 (d, *J* = 8.1 Hz, 1H), 5.34–5.22 (m, 1H), 3.92 (s, 3H), 3.41–3.26 (m, 4H), 2.91–2.83 (m, 4H), 2.55 (s, 3H), 2.31 (s, 3H), 2.05 (s, 3H), 1.59 (d, *J* = 6.9 Hz, 3H). ^13^C NMR (101 MHz, CHLOROFORM-*D*) δ 169.35, 162.66, 148.53, 143.97, 137.32, 136.87, 133.32, 131.98, 127.70, 127.30, 127.06, 125.08, 123.20, 122.46, 118.39, 115.46, 86.45, 79.60, 54.31, 49.11, 48.34, 44.94, 39.28, 36.61, 21.89, 18.95.

#### (*R*)-2-Methyl-*N*-(1-(3-(1-methyl-1*H*-pyrazol-4-yl)-5-((1-methyl-1*H*-pyrazol-4-yl)­ethynyl) phenyl)­ethyl)-5-((1-methylpiperidin-4-yl)­oxy)­benzamide (**18**)

Compound **18** was prepared as an off-white solid following general procedure **C** (15 mg, yield 28%). ^1^H NMR (400 MHz, METHANOL-*D*_4_) δ 8.03–7.98 (m, 1H), 7.87–7.82 (m, 2H), 7.63 (s, 1H), 7.60–7.54 (m, 2H), 7.41–7.33 (m, 1H), 7.21–7.15 (m, 1H), 7.02–6.93 (m, 2H), 5.20 (q, *J* = 7.1 Hz, 1H), 4.66–4.62 (m, 1H), 3.94 (s, 3H), 3.91 (s, 3H), 3.28–3.05 (m, 4H), 2.80 (s, 3H), 2.28 (s, 3H), 2.21–1.93 (m, 4H), 1.56 (d, *J* = 7.1 Hz, 3H). ^13^C NMR (101 MHz, CHLOROFORM-*D*) δ 168.72, 154.48, 143.99, 142.16, 137.78, 136.90, 133.53, 132.82, 132.51, 128.82, 127.53, 127.36, 126.70, 124.64, 123.56, 122.35, 117.12, 115.16, 103.14, 89.76, 81.34, 49.14, 44.29, 39.34, 29.83, 27.84, 21.82, 19.03.

#### 2-Methyl-*N*-((*R*)-1-(3-(1-methyl-1*H*-pyrazol-4-yl)-5-((1-methyl-1*H*-pyrazol-4-yl)­ethynyl) phenyl)­ethyl)-5-(6-methyl-3,6-diazabicyclo­[3.1.1]­heptan-3-yl)­benzamide (**19**)

Compound **19** was prepared as a white solid following general procedure **B** (30 mg, yield 83%). ^1^H NMR (400 MHz, METHANOL-*D*_4_) δ 8.00 (s, 1H), 7.86–7.81 (m, 2H), 7.64–7.57 (m, 2H), 7.57–7.53 (m, 1H), 7.40–7.35 (m, 1H), 7.16 (d, *J* = 8.4 Hz, 1H), 6.85–6.79 (m, 1H), 6.79–6.74 (m, 1H), 5.22 (q, *J* = 7.0 Hz, 1H), 4.40–4.18 (m, 2H), 3.96–3.89 (m, 6H), 3.88–3.79 (m, 1H), 3.71 (s, 3H), 3.14–3.09 (m, 1H), 2.93–2.78 (m, 1H), 2.53–2.36 (m, 2H), 2.27 (s, 3H), 2.15–1.85 (m, 1H), 1.57 (d, *J* = 7.1 Hz, 3H). ^13^C NMR (101 MHz, METHANOL-*D*_4_) δ 146.58, 142.62, 137.47, 134.72, 134.49, 132.70, 129.47, 127.95, 127.69, 125.66, 124.18, 123.67, 110.08, 104.51, 90.66, 81.47, 66.55, 63.42, 50.43, 44.69, 39.10, 39.04, 29.81, 22.25, 18.51. HRMS (ESI) calcd for C_32_H_36_N_7_O [M + H]+, 534.29759; found, 534.29764.

#### *N*-((*R*)-1-(3-(3,6-Dihydro-2*H*-pyran-4-yl)-5-((1-methyl-1*H*-pyrazol-4-yl)­ethynyl)­phenyl)­ethyl)-2-methyl-5-(6-methyl-3,6-diazabicyclo­[3.1.1]­heptan-3-yl)­benzamide (**20**)

Compound **20** was prepared as a white solid following general procedure **B** (28 mg, yield 78%). ^1^H NMR (400 MHz, METHANOL-*D*_4_) δ 7.83 (s, 1H), 7.62 (s, 1H), 7.50–7.43 (m, 1H), 7.44–7.38 (m, 2H), 7.18–7.11 (m, 1H), 6.60–6.53 (m, 1H), 6.52–6.47 (m, 1H), 6.27–6.20 (m, 1H), 5.18 (q, *J* = 7.1 Hz, 1H), 4.36–4.27 (m, 4H), 3.97–3.86 (m, 5H), 3.43–3.35 (m, 2H), 3.30–3.21 (m, 2H), 2.77–2.67 (m, 1H), 2.57–2.45 (m, 2H), 2.42 (s, 3H), 2.24 (s, 3H), 2.08–2.01 (m, 1H), 1.54 (d, *J* = 7.0 Hz, 3H). ^13^C NMR (101 MHz, METHANOL-*D*_4_) δ 172.48, 146.07, 144.34, 142.61, 142.23, 139.16, 134.81, 134.72, 132.85, 128.83, 127.29, 126.12, 125.22, 124.46, 123.69, 117.54, 114.48, 104.48, 90.68, 81.36, 66.79, 65.48, 60.06, 50.93, 50.47, 43.79, 39.09, 28.19, 27.32, 22.25, 18.65. HRMS (ESI) calcd for C_33_H_38_N_5_O_2_ [M + H]+, 536.30200; found, 536.30171.

#### 2-Methyl-*N*-((R)-1-(3′-methyl-5-((1-methyl-1*H*-pyrazol-4-yl)­ethynyl)-[1,1′-biphenyl]-3-yl)­ethyl)-5-(6-methyl-3,6-diazabicyclo­[3.1.1]­heptan-3-yl)­benzamide (**21**)

Compound **21** was prepared as a white solid following general procedure **B** (31 mg, yield 84%). ^1^H NMR (400 MHz, METHANOL-*D*_4_) δ 7.85 (s, 1H), 7.68–7.61 (m, 2H), 7.61–7.56 (m, 1H), 7.52–7.39 (m, 3H), 7.37–7.29 (m, 1H), 7.23–7.12 (m, 2H), 6.83–6.73 (m, 2H), 5.27 (q, *J* = 7.1 Hz, 1H), 4.29–4.24 (m, 1H), 4.07–4.03 (m, 1H), 3.91 (s, 3H), 3.82–3.77 (m, 1H), 3.73–3.50 (m, 3H), 3.13–3.08 (m, 1H), 2.77–2.73 (m, 1H), 2.41 (s, 3H), 2.37–2.20 (m, 5H), 1.87–1.83 (m, 1H), 1.59 (d, *J* = 7.1 Hz, 3H). ^13^C NMR (101 MHz, CHLOROFORM-*D*) δ 166.52, 144.55, 143.88, 142.19, 142.10, 140.18, 138.68, 137.66, 132.80, 132.27, 129.28, 128.92, 128.72, 128.01, 127.75, 125.23, 124.59, 124.46, 124.32, 112.05, 109.18, 103.17, 89.89, 81.22, 61.37, 49.21, 44.16, 39.29, 29.08, 28.50, 21.91, 21.64, 18.77.

#### *N*-((*R*)-1-(3′-Hydroxy-5-((1-methyl-1*H*-pyrazol-4-yl)­ethynyl)-5′-(trifluoromethyl)-[1,1′-biphenyl]-3-yl)­ethyl)-2-methyl-5-(6-methyl-3,6-diazabicyclo­[3.1.1]­heptan-3-yl)­benzamide (**22**)

Compound **22** was prepared as a white solid following general procedure **B** (31 mg, yield 74%). ^1^H NMR (400 MHz, METHANOL-*D*_4_) δ 7.86 (s, 1H), 7.68–7.62 (m, 2H), 7.61–7.52 (m, 2H), 7.37–7.32 (m, 1H), 7.32–7.25 (m, 1H), 7.18–7.12 (m, 1H), 7.07–7.00 (m, 1H), 6.83–6.73 (m, 2H), 5.31–5.20 (m, 1H), 4.28–4.23 (m, 1H), 4.14–3.74 (m, 5H), 3.73–3.45 (m, 3H), 3.17–3.04 (m, 1H), 2.80–2.60 (m, 1H), 2.44–2.13 (m, 5H), 1.83–1.79 (m, 1H), 1.59 (d, *J* = 7.1 Hz, 3H). ^19^F NMR (376 MHz, METHANOL-*D*_4_) δ −64.03. ^13^C NMR (101 MHz, METHANOL-*D*_4_) δ 159.89, 146.99, 146.82, 144.22, 142.70, 141.73, 134.85, 133.54, 133.23, 132.63, 129.77, 129.31, 126.89, 125.88, 125.66, 124.19, 118.55, 115.54, 115.50, 112.80, 112.24, 109.80, 104.38, 90.38, 82.00, 61.98, 50.38, 44.71, 39.10, 30.26, 22.33, 18.50.

#### *N*-((*R*)-1-(3-(2-Amino-5-chloropyridin-3-yl)-5-((1-methyl-1*H*-pyrazol-4-yl)­ethynyl)­phenyl) ethyl)-2-methyl-5-(6-methyl-3,6-diazabicyclo[3.1.1]­heptan-3-yl)­benzamide (**23**)

Compound **23** was prepared as a white solid following general procedure **B** (25.5 mg, yield 65%). ^1^H NMR (400 MHz, METHANOL-*D*_4_) δ 7.98–7.91 (m, 1H), 7.85 (s, 1H), 7.63 (s, 1H), 7.59–7.54 (m, 1H), 7.52–7.46 (m, 1H), 7.46–7.40 (m, 2H), 7.17–7.10 (m, 1H), 6.82–6.74 (m, 2H), 5.22 (q, *J* = 7.0 Hz, 1H), 4.01–3.71 (m, 5H), 3.70–3.36 (m, 4H), 2.69–2.61 (m, 1H), 2.36–2.08 (m, 6H), 1.76–1.72 (m, 1H), 1.57 (d, *J* = 7.1 Hz, 3H). ^13^C NMR (101 MHz, METHANOL-*D*_4_) δ 156.67, 147.17, 147.10, 146.08, 142.69, 140.01, 138.73, 138.43, 134.85, 132.56, 130.85, 129.90, 127.20, 126.19, 123.76, 121.28, 112.70, 109.79, 104.30, 90.20, 82.33, 61.17, 50.41, 44.74, 39.10, 30.55, 22.22, 18.49.

#### *N*-((*R*)-1-(5′-Chloro-2′-fluoro-5-((1-methyl-1*H*-pyrazol-4-yl)­ethynyl)-[1,1′-biphenyl]-3-yl)­ethyl) −2-methyl-5-(6-methyl-3,6-diazabicyclo­[3.1.1]­heptan-3-yl)­benzamide (**24**)

Compound **24** was prepared as a white solid following general procedure **B** (31 mg, yield 78%). ^1^H NMR (400 MHz, METHANOL-*D*_4_) δ 7.85 (s, 1H), 7.64 (s, 1H), 7.59–7.50 (m, 4H), 7.44–7.36 (m, 1H), 7.28–7.19 (m, 1H), 7.17–7.11 (m, 1H), 6.82–6.73 (m, 2H), 5.26 (q, *J* = 7.1 Hz, 1H), 4.40–4.07 (m, 1H), 4.03–3.75 (m, 5H), 3.74–3.37 (m, 3H), 3.18–2.95 (m, 1H), 2.82–2.54 (m, 1H), 2.41–2.07 (m, 5H), 1.79–1.74 (m, 1H), 1.58 (d, *J* = 7.1 Hz, 3H). ^13^C NMR (101 MHz, METHANOL-*D*_4_) δ 159.66 (d, *J* = 247.3 Hz), 146.94, 146.70, 142.69, 136.36, 134.86, 132.59, 131.41, 131.37, 131.24, 131.09, 130.79, 130.76, 130.58, 130.50, 130.00, 127.54, 125.54, 118.97, 118.72, 112.73, 109.80, 104.33, 90.24, 82.08, 61.56, 50.29, 44.76, 39.10, 30.43, 22.23, 18.50.

#### *N*-((R)-1-(3′-Chloro-5-((1-methyl-1*H*-pyrazol-4-yl)­ethynyl)-[1,1′-biphenyl]-3-yl)­ethyl)-2-methyl-5-(6-methyl-3,6-diazabicyclo­[3.1.1]­heptan-3-yl)­benzamide (**25**)

Compound **25** was prepared as a white solid following general procedure **B** (32 mg, yield 83%). ^1^H NMR (400 MHz, METHANOL-*D*_4_) δ 7.85 (s, 1H), 7.68–7.62 (m, 3H), 7.62–7.56 (m, 2H), 7.56–7.52 (m, 1H), 7.49–7.41 (m, 1H), 7.41–7.35 (m, 1H), 7.17–7.10 (m, 1H), 6.82–6.72 (m, 2H), 5.26 (q, *J* = 7.0 Hz, 1H), 4.08 (s, 1H), 4.03–3.72 (m, 5H), 3.69–3.38 (m, 4H), 2.82–2.51 (m, 1H), 2.27 (s, 5H), 1.77–1.73 (m, 1H), 1.59 (d, *J* = 7.1 Hz, 3H). ^13^C NMR (101 MHz, METHANOL-*D*_4_) δ 146.97, 143.65, 142.68, 141.57, 138.51, 135.87, 134.82, 132.59, 131.58, 129.61, 129.34, 128.78, 128.03, 126.54, 125.85, 125.75, 112.71, 109.74, 104.39, 90.45, 81.94, 61.42, 50.38, 44.75, 39.10, 30.46, 22.33, 18.52.

#### 2-Methyl-*N*-(1-(3-(1-methyl-1*H*-pyrazol-4-yl)-5-((1-methyl-1*H*-pyrazol-4-yl)­ethynyl)­phenyl) cyclopropyl)-5-(4-methylpiperazin-1-yl)­benzamide (**26**)

Compound **26** was prepared as a white solid following general procedure **B** (30 mg, yield 83%). ^1^H NMR (400 MHz, DMSO-*D*_6_) δ 8.93 (s, 1H), 8.19 (s, 1H), 8.06 (s, 1H), 7.87 (s, 1H), 7.67 (s, 1H), 7.54–7.46 (m, 1H), 7.35–7.29 (m, 1H), 7.23–7.18 (m, 1H), 7.12–7.05 (m, 1H), 6.96–6.89 (m, 2H), 3.86 (s, 6H), 3.17–3.10 (m, 4H), 2.33–2.17 (m, 7H), 1.39–1.21 (m, 4H). ^13^C NMR (101 MHz, DMSO-*D*_6_) δ 170.01, 148.68, 144.83, 141.18, 137.27, 136.12, 133.64, 132.98, 131.14, 128.21, 125.41, 125.32, 124.55, 123.03, 121.25, 120.98, 116.66, 114.17, 101.57, 89.72, 81.19, 54.37, 48.12, 38.77, 38.72, 34.12, 18.35, 18.07. HRMS (ESI) calcd for C_32_H_36_N_7_O [M + H]+, 534.29759; found, 534.29764.

### Pharmacokinetic and ADME

Profiling of the tested compounds were conducted by Pharmaron Co., Ltd. according to their standard operating procedures (SOPs). Safety profile data of compound **15** was conducted by WuXi Biology according to their standard operating procedures (SOPs).

### SARS-CoV-2 PLpro Expression and Purification

The experimental procedures were conducted as previously described in our earlier work.^28,29^ Briefly, pET11a vector containing SARS-CoV-2 PLpro protein (pp1ab aa 1564–1878) with N-terminal, TEV-cleavable His-tag was transformed into BL21­(DE3) cells and maintained in media containing 100 ug/mL carbenicillin. Protein expression was induced using an autoinduction protocol modified from Studier et al.^51^ Briefly, 1 mL day cultures were used to inoculate a 2 L flask of 500 mL of Super LB containing 100 ug/mL carbenicillin. Cells were grown for 24 h at 25 °C and then harvested by centrifugation. All steps of SARS-CoV2 PLpro purification were performed at 4 °C. Protein yield at each step was monitored by Bradford assay using BSA as a standard. Frozen cells pellets were lysed by sonication in Buffer A (50 mM HEPES, pH 8, 0.5 M NaCl) containing 10 ug/mL lysozyme. The lysate was clarified by centrifugation and loaded onto a 2 mL HiTrap Talon crude column equilibrated with Buffer A. Bound His6-PLpro was eluted with a linear gradient of 0–150 mM imidazole in Buffer A, and fractions containing His6-PLpro were pooled and exchanged into cleavage buffer (20 mM Tris-HCl pH 8.5, 5 mM DTT, 0.5 mM EDTA, 5% glycerol). A 1:100 molar ratio of TEV protease to PLpro was incubated at 4 °C overnight to cleave the His6-tag. To remove the tag and TEV protease, the reaction was loaded onto a UNO-Q column equilibrated with 20 mM Tris HCl, pH 8.5, 3 mM DTT. Cleaved PLpro eluted first in a gradient from 0 to 150 mM NaCl over 20 column volumes. Fractions containing cleaved PLpro were pooled and concentrated to 12 mg/mL, frozen in liquid nitrogen, and stored at −80 °C.

### PLpro Primary Assay

The experimental procedures were conducted as previously described in our earlier work.^28,29^ Briefly, The PLpro primary assay, which measures protease activity with the short peptide substrate Z-RLRGG-AMC (Bachem), was performed in black, flat-bottom 384-well plates containing a final reaction volume of 50 μL. The assays were assembled at room temperature as follows: 40 μL of 50 nM PLpro in Buffer B (50 mM HEPES, pH 7.5, 0.1 mg/mL BSA, 0.01% Triton-X 100, and 5 mM DTT) was dispensed into wells containing 0.1–1 μL of inhibitor in DMSO or appropriate controls. The enzyme was incubated with inhibitor for 10 min prior to substrate addition. Reactions were initiated with 10 μL of 62.5 μM RLRGG-AMC in Buffer B. Plates were shaken vigorously for 30 s, and fluorescence from the release of AMC from peptide was monitored continuously for 15 min on a Tecan Infinite M200 Pro plate reader (λ_excitation_ = 360 nm; λ_emission_ = 460 nm). Slopes from the linear portions of each progress curve were recorded and normalized to plate-based controls. Positive control wells, representing 100% inhibition, included 10 μM GRL0617; negative control wells, representing 0% inhibition, included vehicle alone.

### Cell Culture and Cytotoxicity

The experimental procedures were conducted as previously described in our earlier work.^28,29^ Briefly, The human alveolar epithelial cell line (A549) that stably expresses hACE2 receptor was obtained from BEI Resources (NR-53821). The cells were grown in DMEM supplemented with 10% fetal bovine serum (Gibco), 100 units of penicillin, 100 μg/mL streptomycin (Invitrogen), and 1% nonessential amino acids (NEAA), with 100 μg/mL Blasticidin S. HCl for selection. All cells were grown at 37 °C and 5% CO_2_. Low passage A549 cells (5000 cells/well) were seeded in 96-well plates and incubated at 37 °C and 5% CO_2_ for 24 h prior to a 48 h treatment. All compounds were dissolved in DMSO and final DMSO concentrations never exceeded 1%. The cytotoxicity of compounds (100 μM to 1 μM, 3-fold dilution) was examined using the CellTiter-Glo Luminescent Cell Viability Assay (Promega). Cell cytotoxicity data was normalized to DMSO control as 100% cell viability.

### Virus Production

The experimental procedures were conducted as previously described in our earlier work.^29^ Briefly, SARS-CoV2 strain WA1 (isolate USA-WA1/2020) and strain B.1.617–2 (isolate USA/PHC658/2021) were obtained from BEI Resources and propagated in Vero E6 cells. For the production of viral stocks, cells were infected at an MOI of 0.005 and cultured for 48 h. The cells were harvested with a cell scraper and together with the culture medium spun at 3000 rpm for 10 min. Supernatants were set aside, while the resuspended cell pallets were treated with a Dounce homogenizer and subjected to two freeze–thaw cycles. The homogenates were then recombined with the original supernatants. Following an additional centrifugation step, the supernatants were aliquoted, frozen, and subsequently tittered in serial dilutions by viral plaque assay. All work with SARS-CoV2 was performed under BSL3 conditions in a facility with negative pressure and PPE that included Tyvek suits and N95 masks for respiratory protection.

### Antiviral Activity Assay

The experimental procedures were conducted as previously described in our earlier work.^28,29^ Briefly, A549-hACE2 cells were seeded at 1.5 × 10^5^ cells/well in DMEM complete into 24-well plates (0.5 mL/well) then incubated for 16 h at 37 °C and 5% CO_2_. Cells were pretreated with compound for 1 h prior to infection performed using a clinical isolate of SARS-CoV-2. Test and control compounds were added to the same volume of SARS-CoV-2 (WA1 final MOI = 0.01; BA.1 final MOI = 0.03) and the mixture was added to the monolayer cells and incubated for 1 h at 37 °C and 5% CO_2_. The mixture was removed and replaced with 0.5 mL of infection media and incubated at 37 °C in 5% CO_2_. After 48 h, supernatants and/or lysates were harvested and processed for RT- qPCR.

### RNA Extraction and RT-qPCR

The experimental procedures were conducted as previously described in our earlier work.^28,29^ Briefly, 250 μL of culture fluids were mixed with 750 μL of TRIzol LS Reagent (Thermo Fisher Scientific). RNA was purified following phase separation by chloroform as recommended by the manufacturer. RNA in the aqueous phase was collected and further purified using PureLink RNA Mini Kits (Invitrogen) according to manufacturer’s protocol. Viral RNA was quantified by reverse-transcription quantitative PCR (RT-qPCR) using a 7500 Real-Time PCR System (Applied Biosystems) using TaqMan Fast Virus 1-Step Master Mix chemistry (Applied Bio- systems). SARS-CoV-2 N1 gene RNA was amplified using forward (5′-GACCCCAAAATCAGCGAAAT) and reverse (5′- TCTGGTTACTGCCAGTTGAATCTG) primers and probe (5′- FAM-ACCCCGCATTACGTTTGGTGGACC-BHQ1) designed by the United States Centers for Disease Control and Prevention (oligonucleotides produced by IDT, cat# 10006713). RNA copy numbers were determined from a standard curve produced with serial 10-fold dilutions of RNA standard material of the amplicon region from BEI Resources (NR-52358). All data was normalized to virus alone. All error bars represent SD from three replicates.

### Viral Plaque Assay and Other Virus Quantification

The experimental procedures were conducted as previously described in our earlier work.^29^ Briefly, Infectious virions were quantified by viral plaque assay. To this end, cells were incubated with SARS-CoV2 for 2 h and subsequently overlaid with 1% methylcellulose in culture medium. After 3–4 days, the cells were fixed in 10% formalin for 30 min, washed under tap water, and stained with crystal violet. The number of plaques was counted on a light table.

### Immunofluorescence (IF) Assay for VOC

#### Drug Preparation and Serial Dilution

Test compounds were dissolved in DMSO and sterilized via a 0.22 mL syringe filter. Stock solutions were serially diluted in DMEM (Gibco, #11995065) supplemented with 2% FBS, 1% nonessential amino acids (Gibco, #11140050), and 1% penicillin/streptomycin (Gibco, #15140122). A seven-point dilution series was prepared using a 1:3 fold-reduction, ranging from a maximum concentration of 30 mM to 0.04 mM.

#### Cell Culture and Viral Infection (VOC Delta and Omicron)

hACE2-A549 cells (kindly provided by B. R. TenOever, Icahn School of Medicine at Mount Sinai to Vlad Nicolaescu, Department of Microbiology-Howard Taylor Ricketts Laboratory (HTRL) at University of Chicago) were seeded in 96-well plates. The cells were pretreated with the drug dilutions for 2 h prior to viral challenge. Subsequently, cells were infected with SARS-CoV-2 (Delta or Omicron variants) at a multiplicity of infection (MOI) of 0.5. Plates were incubated at 37 °C for 48 h and then fixed using a 10% formalin solution.

#### Immunofluorescence Staining and Imaging

Fixed cells were processed for immunofluorescence (IF) staining. Primary staining was performed using an anti-SARS-CoV-2 Spike Protein [1A9] antibody (GeneTex, #GTX632604) at a 1:3000 dilution, followed by an Alexa Fluor 488-conjugated secondary antibody (Invitrogen, #A-11001). Nuclei were counterstained with DAPI to facilitate total cell quantification. Images were acquired using an Olympus CKX53 microscope.

#### Data Analysis

Infection efficiency was quantified using ImageJ by calculating the ratio of Spike protein-positive cells to the total number of DAPI-stained nuclei. Results were normalized against untreated control wells to determine relative viral inhibition.

### Animal Studies

The experimental procedures were conducted as previously described in our earlier work.^28,29^ Briefly, All mouse studies with infectious virus were carried out in the Howard T. Ricketts Laboratory (HTRL) RG3 research core at the University of Chicago in strict accordance with the recommendations in the Guide for the Care and Use of Laboratory Animals of the National Institutes of Health. The University of Chicago is an AAALAC international accredited animal care organization, and all experiments were approved by the Institutional Animal Care and Use Committee (IUACUC) of the University of Chicago under protocol 14–521. Pharmaron Inc. is accredited with AAALAC. All the procedures related to animal handling, care, and treatment in this study were performed according to guidelines and animal use protocols (ON-CELL-XEN-06012023 and PK-M-07182022) approved by the IACUC of Pharmaron.

### PK and Tolerability Studies in Mice

The experimental procedures were conducted as previously described in our earlier work.^29^ Briefly, C57*B*/6 mice (6–8 weeks) were provided by Vital River Corp. (China) and were used for PK or tolerability studies, which were carried out by Pharmaron Inc., Beijing, China. All animals were group-housed at 3/cage. Access to food and water was provided ad libitum. Animals were monitored for body weight daily, pain or distress or other vital signs throughout the study. All blood collection was done using series bleeding via dorsal metatarsal veins using an EDTA-K2 tube at predetermined time points and stored on ice until centrifugation to obtain plasma, which was stored frozen at −20 °C or lower. At the completion of the study, animals were euthanized by overdose of inhaled anesthesia followed by exsanguination. In PK studies, male mice and SD rats were used. Compounds **15**, **19**, **20** and **26** were used for snapshot or full PK studies by PO (10 mg/kg) and IV (1 mg/kg) injection for **15**. Vehicle for PO was 0.5% methylcellulos plus 2% Tween-80. In the IV studies, the vehicle was 10% DMSO, 40% PEG400, 50% water. In the tolerability study, **15** was used. Female mice were dosed by PO of compound **15** (100 or 150 mg/kg, BID) or nirmatrelvir (1000 mg/kg, BID) for 5 days. Plasma and lungs were collected at 1 h postdosing (100 or 150 mg/kg PO) lungs were immediately frozen in liquid nitrogen. Plasma and lung homogenates were extracted with acetonitrile, analyzed, and quantified by LC-MS/MS (see supplement section). A noncompartment model was used to obtain PK parameters in WinNonlin 8.3.

### Mice, Drug Treatment, and SARS-CoV-2 Infection *In Vivo*

Female K18-ACE2 mice (strain B6.Cg-Tg­(K18-ACE2)­2Prlmn/J), 6–8 weeks of age, were housed in the Howard Taylor Ricketts Laboratory (HTRL) RG3 Research Core at the University of Chicago under IACUC protocol 72642. Animals were anesthetized using vaporized isoflurane and challenged intranasally with 20,000 PFU of SARS-CoV-2 (strain WA1). Drug treatment was initiated with 1 h postinfection. Compounds were formulated in a vehicle containing 0.5% methylcellulose and 2% Tween-80 and administered orally (p.o.) twice daily until day 5. Body weight and health status were monitored daily. On day 5, 1 h following the final compound administration, animals were euthanized. Lungs were harvested, with one lobe used to enumerate viral PFU via plaque assay and the second fixed in formalin for histological validation.

### Statistical Analysis

The experimental procedures were conducted as previously described in our earlier work.^28,29^ Briefly, GraphPad Prism 8 software package (GraphPad Software, USA) was used to perform statistical analysis. All data were presented as the mean ± SD unless otherwise noted. One-way analysis of variance (ANOVA) with appropriate posthoc tests (3+ groups) and Student’s *t* test (2 groups) were used to calculate statistical significance: *P < 0.05, **P < 0.01, ***P < 0.001.

## Supplementary Material





## Notes

Further information on synthesis and compound characterization, stability, pharmacokinetic and safety profiles, and HPLC of representative compounds (PDF)
Molecular formula strings (CSV)

## Abbreviations

3CLpro: 3C-like protease
ACE2: angiotensin-converting enzyme 2
ADME: absorption, distribution, metabolism and excretion
AMC: 7-amino-4-methylcoumarin
ANOVA: one-way analysis of variance
BID: bis in die (twice daily)
BSA: bovine serum albumin
BSL3: biosafety level 3
CDC: Centers for Disease Control and Prevention
COVID-19: coronavirus disease 2019
CYP: cytochrome P450
DAPI: 4′,6-diamidino-2-phenylindole
DIAD: diisopropyl azodicarboxylate
DMAP: 4-dimethylaminopyridine
DMEM: Dulbecco’s modified Eagle’s medium
DMSO: dimethyl sulfoxide
DTT: dithiothreitol
EDTA: ethylenediaminetetraacetic acid
FBS: fetal bovine serum
FDA: Food and Drug Administration
hACE2: human angiotensin-converting enzyme 2
HATU: hexafluorophosphate azabenzotriazole tetramethyl uronium
HEPES: 4-(2-hydroxyethyl)-1-piperazineethanesulfonic acid
HIV: human immunodeficiency virus
IACUC: Institutional Animal Care and Use Committee
IF: immunofluorescence
i.v.: intravenous
LCMS: liquid chromatography–mass spectrometry
LC-MS/MS: liquid chromatography-tandem mass spectrometry
MOI: multiplicity of infection
Mpro: SARS coronavirus main proteinase
NEAA: nonessential amino acids
Nsp: nonstructural proteins
PCC: post-COVID-19 condition
PDB: protein data bank
PEG400: polyethylene glycol 400
PFU: plaque-forming unit
PK: pharmacokinetic
PLpro: papain-like protease
p.o.: oral
RdRp: RNA-dependent RNA polymerase
RT-qPCR: reverse transcription quantitative polymerase chain reaction
SARS-CoV-2: severe acute respiratory syndrome coronavirus 2
SD: standard deviation
SOP: standard operating procedure
TEA: triethyl amine
TEV: tobacco etch virus
TLC: thin-layer chromatography
Ub: ubiquitin
VOC: variants of concern
XPhos: 2-dicyclohexylphosphino-2′,4′,6′-triisopropylbiphenyl
