# Supplementary material for: Discovery of the SARS-CoV‑2 Papain-Like Protease Inhibitor MR1–114: From Structure-Based Design to In Vivo Antiviral Efficacy
Source: J Med Chem. 2026 Mar 26;69(7):8433–50. doi: 10.1021/acs.jmedchem.5c03846 (PMC13071871; doi:10.1021/acs.jmedchem.5c03846)
Supplement: Supplementary file 1 [file jm5c03846_si_001.pdf]

Supporting Information

Discovery of the SARS-CoV-2 PLpro Inhibitor MR1-114: From Structure-Based Design to *in Vivo* Antiviral Efficacy

Malla Reddy Gannarapu[1≠], Divakar Indukuri[1≠], Cameron Holberg[1], Kiira Ratia[3], Omar Lozano Ramos[1], Savio Cardoza[1], Ganga Reddy Velma[1], Soumya Reddy Musku[1], Steve Slilaty[4], Zuomei Li[4], Lijun Rong[5], Gregory R J Thatcher[1,2]* Rui Xiong[1]*

[1]Department of Pharmacology & Toxicology, R. Ken Coit College of Pharmacy, University of Arizona, Tucson 85721, AZ, USA.

[2]Department of Chemistry & Biochemistry, Colleges of Science and Medicine, University of Arizona, Tucson 85721, AZ, USA.

[3]Research Resources Center, University of Illinois Chicago (UIC), Chicago 60612, IL, USA.

[4]Sunshine Biopharma Inc., 333 Las Olas Way, CU4 Suite 433, Fort Lauderdale, FL33301, USA.

[5]Department of Microbiology, College of Medicine, University of Illinois Chicago 60612 (UIC), IL, USA.

*Corresponding authors:

Rui Xiong, xiongr@arizona.edu

Gregory Thatcher, grjthatcher@arizona.edu

Contents of SI

Tables (Stability, Pharmacokinetic, and Safety Profiles)	S2-S4
Supplementary synthesis and characterization of compounds	S5-S25
NMR Spectra	S26-S44
HPLC Traces	S45-S50

**Table S1.** The stability results of test compounds and control compound propantheline in human whole blood							
Compound ID	Species	$T_{1/2}$ (min)	Remaining Percentage (%)				
			0 min	15 min	30 min	60 min	120 min
**Propantheline**	Human	33.40	100.00	88.75	59.40	30.35	6.66
**15**	Human	495.96	100.00	92.82	94.87	87.95	83.33
**20**	Human	410.83	100.00	101.61	103.63	91.94	84.19
**26**	Human	> 511.69	100.00	97.14	95.36	91.43	86.43
Note: when the remaining percentage of the last time point is > 85%, $T_{1/2}$ (min) is reported as > 511.69 min. Test Concentration (5 μM)							

**Table S2**. POB Pharmacokinetic Profile of **15** in C57BL6 Mouse (10 mg/kg)						
Brain concentration-time data						
Time	Concentration (ng/g)			Mean	SD	CV
(h)	Mouse 7	Mouse 8	Mouse 9	(ng/g)	(ng/g)	(%)
1	6.50	6.20	7.20	6.63	0.51	7.74
Plasma concentration-time data						
Time	Concentration (ng/mL)			Mean	SD	CV
(h)	Mouse 7	Mouse 8	Mouse 9	(ng/mL)	(ng/mL)	(%)
1	212	270	328	270	58	21.5
POB Brain/Plasma Ratio Profile						
Time	Brain/Plasma Ratio			Mean	SD	CV
(h)	Mouse 7	Mouse 8	Mouse 9	(ng/g)	(ng/g)	(%)
1	0.0307	0.0230	0.0220	0.0252	0.0048	18.9
Lung concentration-time data						
Time	Concentration (ng/g)			Mean	SD	CV
(h)	Mouse 7	Mouse 8	Mouse 9	(ng/g)	(ng/g)	(%)
1	1630	1315	1815	1587	253	15.9
POB Lung /Plasma Ratio Profile						
Time	Lung/Plasma Ratio			Mean	SD	CV
(h)	Mouse 7	Mouse 8	Mouse 9	(ng/g)	(ng/g)	(%)

1	7.69	4.87	5.53	6.03	1.47	24.4

**Table S3**. Safety profile data of **15**					
Target Name	Reference Data				**15**
	Reference ID	IC_{50} (nM)	MaxDose (nM)	%Inh@MaxDose	Ave%Inh@10.0 μM
Alpha2A	yohimbine	4.943	1000	96.95	46.38
NET	protriptyline	7.969	1000	101.41	1.41
V1A	[d(CH2)51,Tyr(Me)2]-AVP	1.777	100	100.24	1.21
CCKa	CCK-8s	1.234	100	100	-14.67
NMDA	MK-801	16.29	1000	100.26	-10.6
Ca2+-L	nitrendipine	1.229	100	90.11	22.44
5HT2B	(±)DOI	60.08	10000	98.19	20.31
5-HTT	imipramine	11.375	1000	103.19	1.32
DAT	BTCP	16.285	1000	109.86	14.26
hERG	Dofetilide	4.818	1000	104.76	55.81
5-HT2A	Ketanserin	1.857	100	102.27	66.81
H1	Pyrilamine	8.889	1000	98.9	12.62
M1	Pirenzepine	28.936	1000	100.19	14.51
M2	Methoctramine	3.426	1000	82.42	57.64
M3	4-DAMP	1.563	250	98.32	10.64
AR	progesterone	32.015	1000	99.82	14.31
GR	dexamethasone	3.009	100	98.14	-5.44
ADORA2A	CGS 15943	8.875	1000	96.58	4.74
Alpha1A	WB 4101	0.77	100	100.85	46.49
GABAA	flumazenil	3.728	1000	101.4	-23.24
D1	SCH 23390	1.31	100	99.28	26.45
D2L	7-OH-DPAT	7.769	1000	103.56	66.30
op-delta	naltrindole	0.457	100	99.13	4.74
op-kappa	U-50488	3.052	1000	99.81	16.96
op-mu	DAMGO	0.512	100	96.01	26.88
5-HT3	MDL 72222	26.09	2500	99.76	4.77
5-HT1B	serotonin	8.838	2500	100.91	61.89
H2	cimetidine	6499	50000	104.54	5.26
Beta1	atenolol	901.97	100000	96.42	0.60
Beta2	ICI 118551	6.449	1000	101.85	-0.93
nACHR-Alpha7	MLA	8.872	1000	100.6	6.81
5-HT1A	8-OH-DPAT	1.176	100	101.25	95.42

CB1	CP 55940	1.37	500	72.19	19.15
CB2	WIN 55212-2	3.301	500	95.36	40.05
Na+	Tetrodotoxin	10.525	1000	98.68	26.36
Kv	Charybdotoxin	8.208	1000	100.29	-19.82
ACHE	Neostigmine bromide	63.79	10000	105.97	7.96
COX1	Diclofenac	93.12	5000	97.14	13.4
COX2	NS-398	529.05	150000	98.6	20.00
LCK	stausporine	5.048	500	107.09	4.22
MAO-A	Toloxatone	1281	100000	99.14	-15.36
PDE3A	Trequinsin	0.084	20	100.04	14.89
PDE4D2	Roflumilast	0.387	20	100.01	21.69
Eta Antagonist	BQ-123	3.543	75	100.31	9.86
	Reference Data				**15**
	Reference ID	EC_{50} (nM)	MaxDose (nM)	Act%@MaxDose	Ave%Act@12uM
Eta Agonist	Endothelin 1	2.37	300	109.4	0.39

Supplementary synthesis and characterization of compounds

General Procedure D (ketone to sulfinamide)[1]

To a solution of ketone (example **S1**, 1.0 equiv.), (*S*)-2-methylpropane-2-sulfinamide (3.0 equiv.) in THF (40 mL) was added $Ti(OEt)_4$ (3.0 equiv.). The mixture was degassed and purged with N_2 for 3 times, and then the mixture was stirred at 70 °C for 16 h under N_2 atmosphere. LC-MS showed 48% of desired MS was detected. The mixture was cooled down to 25 °C and then EtOAc (100 mL) was added to the mixture. The mixture was cooled down to 0 °C and water (100 mL) was added. A lot of white solids appeared. The solid was removed by filtration. The organic phase was separated. The aqueous phase was further extracted with EtOAc (100 mL × 2). The combined organic layers were washed by brine and dried over sodium sulfate. Then the mixture was concentrated under reduced pressure to give a residue. The residue was purified by flash silica gel chromatography (ISCO®; 40.0 g SepaFlash® Silica Flash Column, Eluent of 0~20% EtOAc /Petroleum ether gradient @ 60 mL/min) to give the imine intermediate. In continuation, to a solution of imine intermediate (example **S2**, 1.0 equiv.) in THF was added L-selectride (1 M in THF, 1.5 equiv.) at -70 °C, then the yellow solution was stirred at -70 °C for 2 h and 25 °C for 12 h under N_2. TLC (PE: EtOAc = 0: 1, UV) showed the starting material was consumed and one new spot with larger polarity was detected. The mixture was quenched slowly with sat. aq. NH_4Cl (50 mL) at -10 °C-0 °C and stirred at 0 °C for 15 min under N_2. The mixture was diluted with water (60 mL) and extracted with EtOAc (80 mL x 2). The combined organic phase was washed with 1% NaClO (50 mL x 2), dried over anhydrous Na_2SO_4, filtered and the filtrate was concentrated under reduced pressure to give a crude. The residue was purified by flash silica gel chromatography

(ISCO®; 20 g SepaFlash® Silica Flash Column, Eluent of 100% Ethyl acetate/Petroleum ether to 10% Methanol/Ethyl acetate gradient@ 50 mL/min) to give (*S*)-sulfinamide (example **S3**). [Note: usage of chlorinated solvents resulted mixture of protected and de-protected products]

General Procedure E (reductive amination)

To a solution of secondary amine (1.0 equiv.) in MeOH (1.6 mL) was added formaldehyde (6 equiv., 37% Wt) and Formic acid (1 drop). The reaction mixture was stirred at 25 °C for 0.5 h. To the reaction mixture was added sodium triacetoxyborohydride (6 equiv.) portion-wise. The reaction mixture was stirred for 0.5 h at 25 °C. On completion of reaction, solvent was evaporated, followed by addition of satd. $NaHCO_3$ (6 mL). The organic components were extracted with EtOAc (3 x 10 mL). The combined organic layer was dried and evaporated to give a crude mixture. Purification by preparative HPLC (column: C18 50×30 mm; mobile phase: [Water (Formic acid)-Acetonitrile]; flow rate = 15 ml/min gradient: 20%-45% B over 10 min) afforded desired methyl-substituted product.

General Procedure F (acid-amine coupling)

Amine compound, acid compound, HATU or EDC, TEA (or DIPEA) and DMAP were dissolved in dry DMF or DCM and stirred at room temperature overnight. The mixture was diluted with ethyl acetate and was then washed with saturated aq. $NaHCO_3$, water, and brine, respectively. The organic layer was dried over Na_2SO_4, filtered, and concentrated. The residue was purified by silica gel column chromatography or Prep-HPLC to provide the desired amide/ester.

General Procedure G (*N*-Boc or *N*-sulfinyl deprotection)

To a solution of Boc-protected or *N*-sulfinyl compound in DCM was added HCl (4M in dioxane) at 0 °C and then warmed up to room temperature. After stirring for another 2 h, the reaction was dried under vacuum. Prep-HPLC was used to purify the residue, yielding the deprotected compound.

General Procedure H (ester hydrolysis)

To a solution of methyl-ester (1.0 equiv.) in THF (2 mL), MeOH (1 mL) and water (0.5 mL) was added LiOH.H_2O (3 equiv.). The reaction was stirred at room temperature for 12 h. On completion of reaction as checked by TLC (MeOH/DCM=1:9, UV), the reaction mixture was evaporated and diluted with water (5 mL). 1 N HCl was added to the reaction mixture followed by addition of EtOAc (5 mL). The organic layer was separated, dried using sodium sulfate and evaporated to give crude product. Used for next reaction without further purifications.

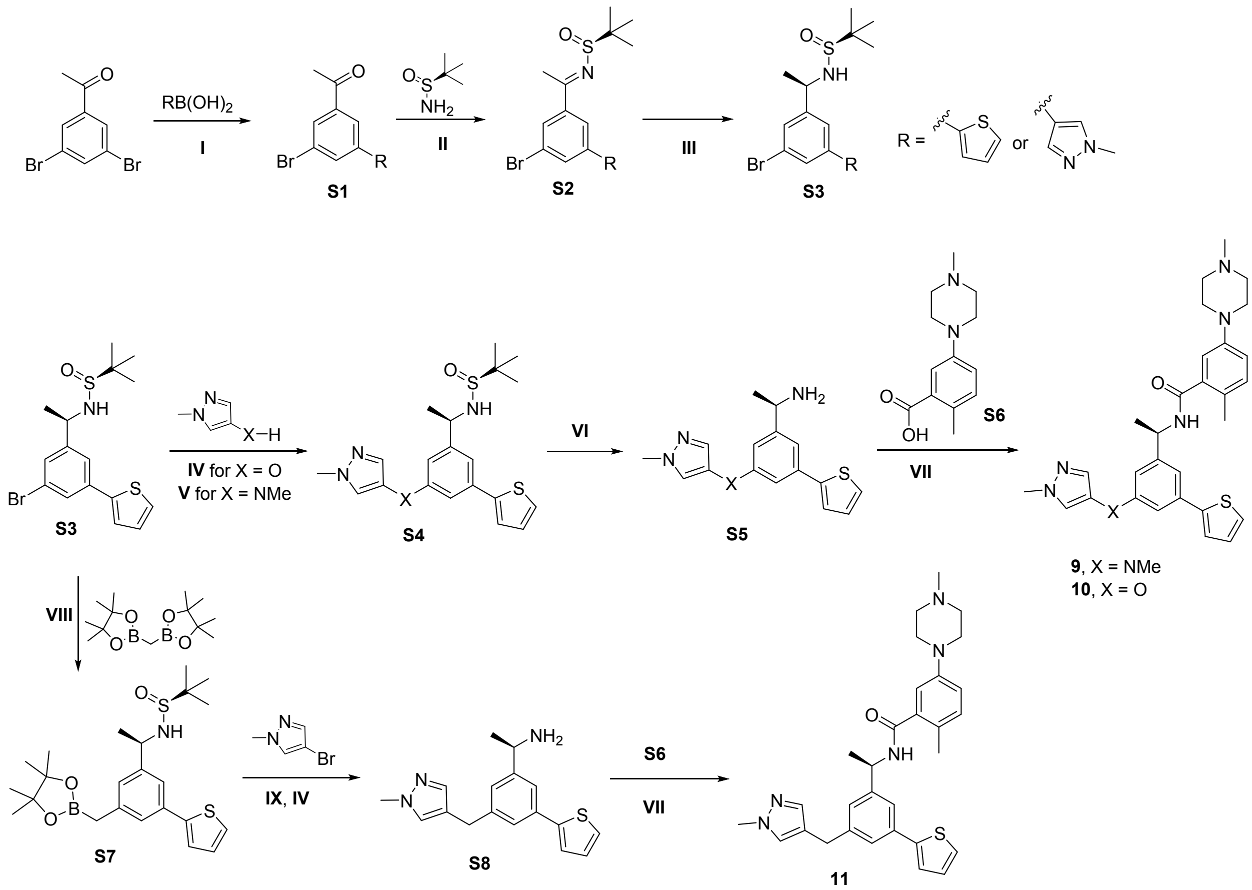

Scheme 1. Synthesis of **9-11**. Reagents and conditions: (I) $Pd(PPh_3)_4$, Na_2CO_3, Toluene/EtOH/H_2O, 90 °C, 12 h; (II) $Ti(OEt)_4$, THF, 25-70 °C, 14 h; (III) L-Selectride, THF, -70 °C, 30 min; (IV) *N*,*N*-dimethylglycine hydrochloride, CuI, Cs_2CO_3, 120 °C, 12 h; (V) RuPhos Pd G4, Cs_2CO_3, dioxane, 100 °C, 16 h; (VI) HCl/dioxane, Dioxane, 25 °C, 2 h; (VII) HATU, DIPEA, DMF, 25 °C, 1 h; (VIII) CATACXIUM(R) A PD G3, Cs_2CO_3, H_2O/dioxane, 80 °C, 1 h; (IX) $Pd(AcO)_2$, K_3PO_4, SPhos, dioxane/ H_2O, 60 °C, 3 h.

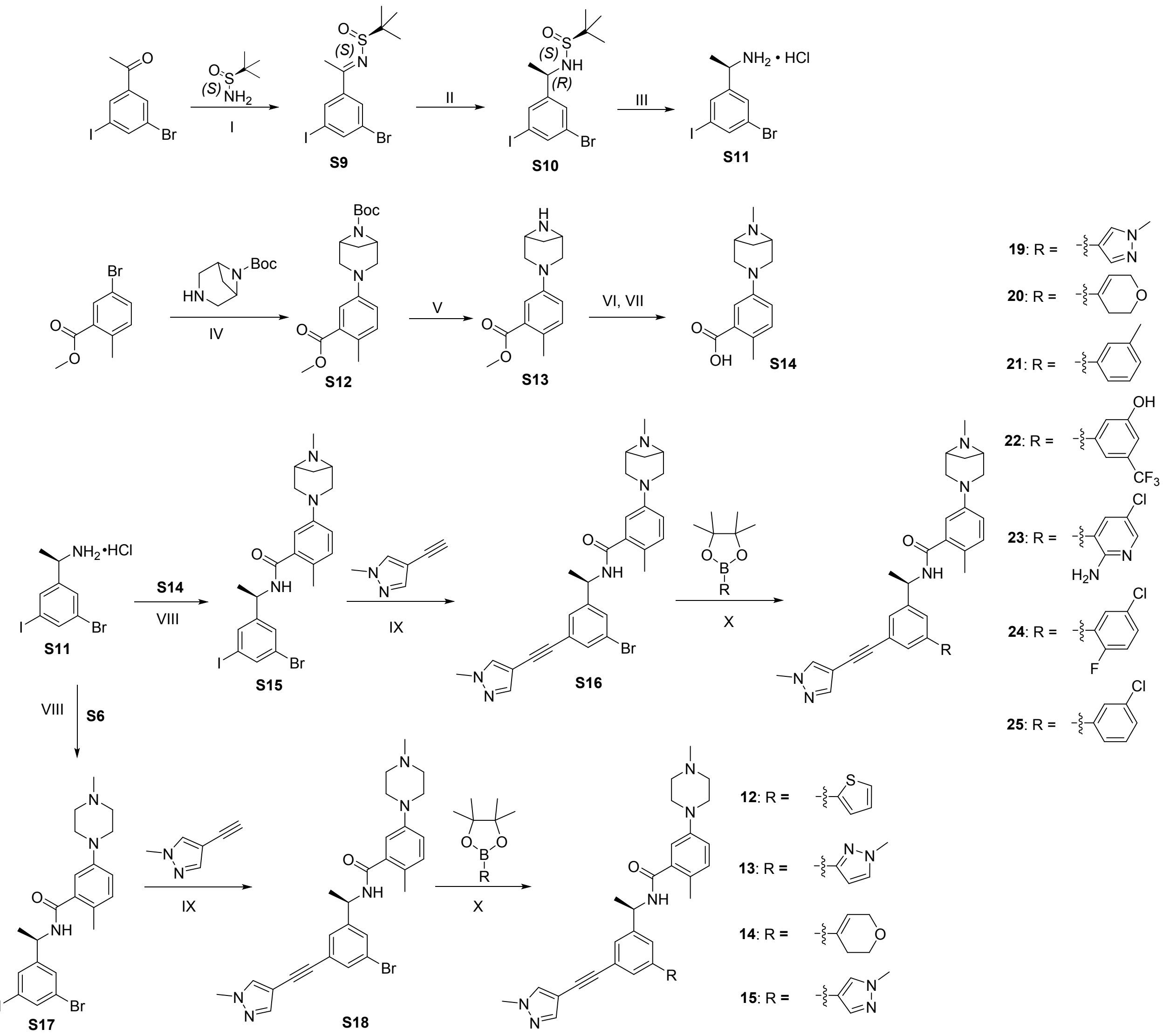

Scheme 2. Synthesis of **12-15** and **19-25.** Reagents and conditions: (I) $Ti(OEt)_4$, THF, 25-70 °C, 14 h; (II) L-Selectride, THF, -70-25 °C, 14 h; (III) HCl/dioxane, Dioxane, 25 °C, 4 h; (IV) RuPhos Pd G3, Cs_2CO_3, Dioxane, 100 °C, 6 h; (V) TFA, DCM, r.t., 1-2 h; (VI) Formaldehyde, Formic acid, $NaBH(OAc)_3$, MeOH, r.t., 1 h; (VII) $LiOH.H_2O$, THF, MeOH, Water, r.t., 12 h; (VIII) HATU, TEA, DMAP, DMF, r.t., 1 h; (IX) $Pd(PPh_3)_2Cl_2$, CuI, TEA, THF, rt, 30 min; (X) XPhos Pd G2, Cs_2CO_3, Dioxane/Water (3:1), Ar atm., 90 °C, 1 h.

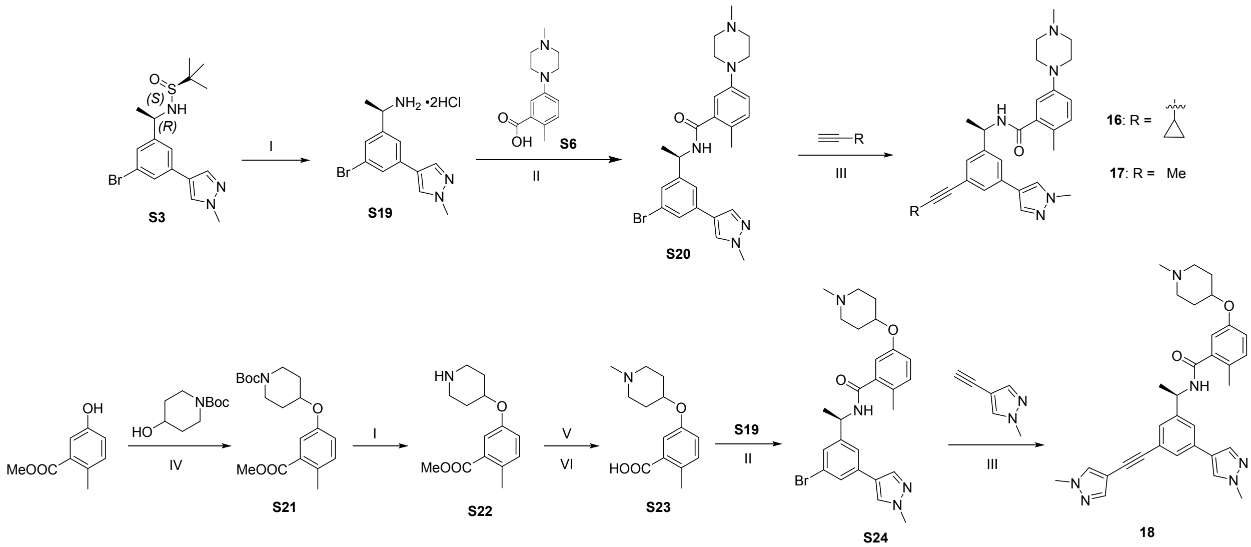

Scheme 3. Synthesis of **16**-**18**. Reagents and conditions: (I) HCl/dioxane, Dioxane, 25 °C, 2 h; (II) HATU, TEA, DMAP, DMF, r.t., 1 h; (III) [Pd(CH_3CN)$_2$]Cl_2, Xphos, Cs_2CO_3, ACN, 85°C, 4 h; (IV) Triphenylphosphine, DIAD, THF, r.t., 12 h; (V) Formaldehyde, Formic acid, NaBH(OAc)$_3$, MeOH, r.t., 1 h; (VI) LiOH.H_2O, THF, MeOH, Water, r.t., 12 h.

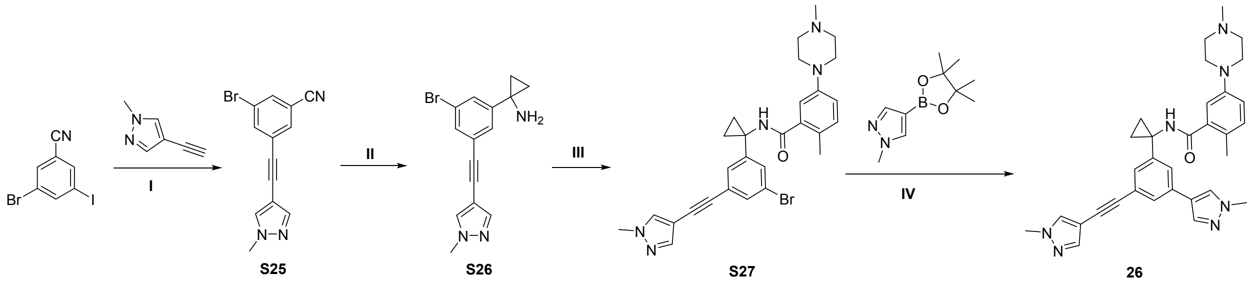

Scheme 4. Synthesis of **26.** Reagents and conditions: (I) Pd(PPh_3)$_2Cl_2$, CuI, TEA, THF, r.t., 30min; (II) EtMgBr, Ti(OEt)$_4$, BF_3.OEt_2, -78 °C to r.t.; (III) **S6**, HATU, DMAP, TEA, DMF, r.t.; (IV) XPhos Pd G2, Cs_2CO_3, Dioxane/Water (3:1), Ar atm., 90 °C, 1 h.

Synthesis of 1-(3-bromo-5-(thiophen-2-yl)phenyl)ethan-1-one (S1a)

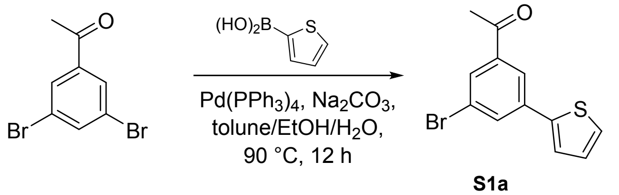

S1a

To a solution of 1-(3,5-dibromophenyl)ethanone (8.00 g, 28.8 mmol) and 2-thienylboronic acid (3.31 g, 25.9 mmol) in EtOH (70 mL)/water (35 mL)/toluene (175 mL) were added Na_2CO_3 (6.10 g, 57.6 mmol) and $Pd(PPh_3)_4$ (1.66 g, 1.44 mmol). The mixture was degassed and purged with N_2 for 3 times, and then the mixture was stirred at 90 °C for 12 h under N_2 atmosphere. TLC (PE/EtOAc = 10/1, R_f = 0.64) showed the 2-thienylboronic acid was consumed. The reaction mixture was concentrated under reduced pressure to give residue. The residue was purified by flash silica gel chromatography (ISCO®; 40.0 g SepaFlash® Silica Flash Column, Eluent of 0~3% EtOAc/Petroleum ether gradient @ 100 mL/min) to give 1-[3-bromo-5-(2-thienyl)phenyl]ethanone **S1a** (4.49 g, 16.0 mmol, 55.5% yield) as a yellow oil. ^{1}H NMR (400 MHz, DMSO-d_6) δ = 8.12-8.04 (m, 2H), 7.97 (s, 1H), 7.72 (d, J = 3.2 Hz, 1H), 7.66 (d, J = 5.2 Hz, 1H), 7.19-7.17 (m, 1H), 2.63 (s, 3H).

Synthesis of (1*R*)-1-[3-(1-methylpyrazol-4-yl)oxy-5-(2-thienyl)phenyl]ethanamine (S5a):

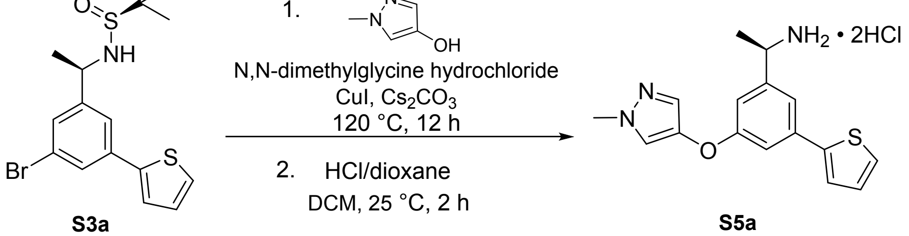

S3a **S5a**

Intermediate **S3a** was prepared as a yellow oil following general procedure D. To a solution of (*S*)-*N*-[(1*R*)-1-[3-bromo-5-(2-thienyl)phenyl]ethyl]-2-methyl-propane-2-sulfinamide **S3a** (600 mg, 1.55 mmol, 1.0 equiv.), 1-methylpyrazol-4-ol (229 mg, 2.33 mmol) in dioxane (30 mL) were added 2-(dimethylamino)acetic acid; hydrochloride (219 mg, 1.57 mmol), Cs_2CO_3 (1.26 g, 3.88 mmol) and CuI (148 mg, 776 μmol). The mixture was degassed and purged with N_2 (3 times), and then the mixture was stirred at 120 °C for 12 h under N_2 atmosphere. LCMS showed 34% of desired MS was detected. The reaction mixture was diluted with water (20 mL) and was extracted with EtOAc (20 mL × 3). The combined organic layers were washed with brine and dried over sodium sulfate. Then the mixture was concentrated under reduced pressure to give a residue. The residue was purified by flash silica gel chromatography (ISCO®; 20.0 g SepaFlash® Silica Flash Column, Eluent of 0~60% EtOAc/Petroleum ethergradient @ 60 mL/min) to give (*S*)-2-methyl-*N*-[(1*R*)-1-[3-(1-methylpyrazol-4-yl)oxy-5-(2-thienyl)phenyl]ethyl]propane-2-sulfinamide, which was de-protected by following general procedure G to give (1*R*)-1-[3-(1-methylpyrazol-4-yl)oxy-5-(2-thienyl)phenyl] ethanamine **S5a** (113 mg, 329 μmol, 69.9% yield, 97.8% purity, HCl) was obtained as a brown gum. Retention time = 2.657 min. ^{1}H NMR (400 MHz, DMSO-d_6) δ = 8.61 (s, 3H), 7.82 (s, 1H), 7.61-7.56 (m, 2H), 7.54 (d, *J* = 2.8 Hz, 1H), 7.41 (s, 1H), 7.22 (s, 1H), 7.17-7.12 (m, 2H), 4.47-4.36 (m, 1H), 3.83 (s, 3H), 1.51 (d, *J* = 6.8 Hz, 3H). LC-MS: 300.1 $[M+H]^+$.

Synthesis of *N*-[3-[(1*R*)-1-aminoethyl]-5-(2-thienyl)phenyl]-*N*,1-dimethyl-pyrazol-4-amine (S5b)

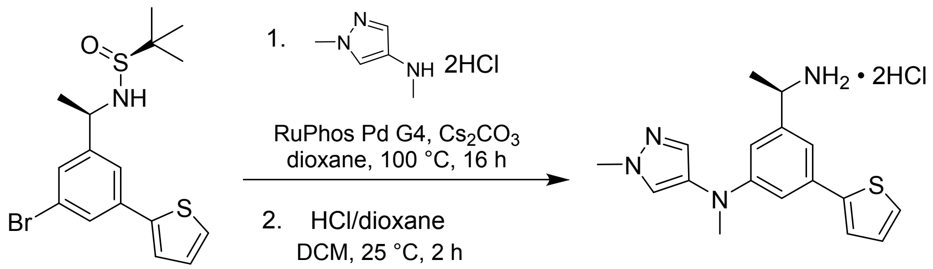

S3a **S5b**

To a solution of (*S*)-*N*-[(1*R*)-1-[3-bromo-5-(2-thienyl)phenyl]ethyl]-2-methyl-propane-2-sulfinamide **S3a** (700 mg, 1.81 mmol) and *N*,1-dimethylpyrazol-4-amine (267 mg, 1.81 mmol, HCl) in dioxane (7 mL) was added Cs_2CO_3 (2.36 g, 7.25 mmol), followed by addition of RuPhos Pd G4 (154 mg, 181 μmol) to the mixture. The mixture was degassed and purged with N_2 for 3 times. The mixture was stirred at 100 °C for 16 h under N_2 atmosphere. LCMS showed 61% of desired MS was detected. The reaction mixture was diluted with water (20 mL) and was extracted with EtOAc (20 mL × 3). The combined organic layers were washed by brine and died over sodium sulfate. Then the mixture was concentrated under reduced pressure to give a residue. The residue was purified by flash silica gel chromatography (ISCO®; 20.0 g SepaFlash® Silica Flash Column, Eluent of 0~70% EtOAc/Petroleum ether gradient @ 60 mL/min) to give (*S*)-2-methyl-*N*-[(1*R*)-1-[3-[methyl-(1-methylpyrazol-4-yl)amino]-5-(2-thienyl)phenyl]ethyl]propane-2-sulfinamide (510 mg, 1.19 mmol, 65.5% yield, 97.0% purity) as a yellow gum, which was de-protected by following general procedure G to give *N*-[3-[(1*R*)-1-aminoethyl]-5-(2-thienyl)phenyl]-*N*,1-dimethyl-pyrazol-4-amine **S5b** (392 mg, 1.10 mmol, 99.8% yield, 98.0% purity, HCl) as a light-yellow solid. Rt = 2.751 min; LCMS: 313.1 $[M+H]^+$. ^1H NMR (400 MHz, DMSO-d_6) δ = 8.49 (s, 3H), 7.76 (s, 1H), 7.55-7.50 (m, 1H), 7.45-7.40 (m, 2H), 7.18 (s, 1H), 7.12 (dd, *J* = 3.6, 5.2 Hz, 1H), 6.96 (d, *J* = 8.8 Hz, 2H), 4.30-4.27 (m, 1H), 3.84 (s, 3H), 3.23 (s, 3H), 1.50 (d, *J* = 6.8 Hz, 3H).

Synthesis of (1*R*)-1-[3-[(1-methylpyrazol-4-yl)methyl]-5-(2-thienyl)phenyl] ethanamine (S8)

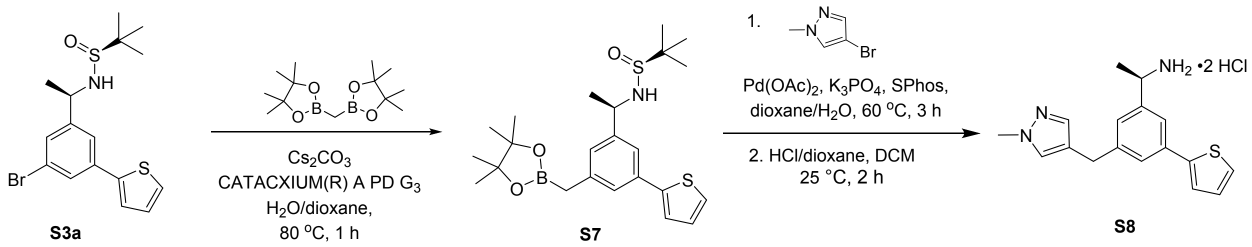

To a solution of 4,4,5,5-tetramethyl-2-[(4,4,5,5-tetramethyl-1,3,2-dioxaborolan-2-yl)methyl]-1,3,2-dioxaborolane (1.39 g, 5.18 mmol), Cs_2CO_3 (2.53 g, 7.76 mmol) and (*S*)-*N*-[(1*R*)-1-[3-bromo-5-(2-thienyl)phenyl]ethyl]-2-methyl-propane-2-sulfinamide **S3a** (1.00 g, 2.59 mmol) in dioxane (10 mL) and water (1 mL) was added CATACXIUM(R) A PD G3 (188 mg, 259 μmol) under N_2, the mixture was stirred at 80 °C for 1 h under N_2.[2] LCMS showed that 49% of desired MS was detected. The reaction mixture was diluted with water (20 mL) and was extracted with EtOAc (20 mL × 3). The combined organic layers were washed by brine and died over sodium sulfate. Then the mixture was concentrated under reduced pressure to give a residue. The residue was purified by flash silica gel chromatography (ISCO®; 12.0 g SepaFlash® Silica Flash Column, Eluent of 0~50% EtOAc/Petroleum ethergradient @ 60 mL/min) to give (*S*)-2-methyl-*N*-[(1*R*)-1-[3-[(4,4,5,5-tetramethyl-1,3,2-dioxaborolan-2-yl)methyl]-5-(2-thienyl)phenyl]ethyl]propane-2-sulfinamide **S7** (580 mg, 1.30 mmol, 50.1% yield) as a yellow oil. t_R = 0.615 min, LCMS: 447.7 $[M+H]^+$.

To a solution of 4-bromo-1-methyl-pyrazole (477 mg, 2.96 mmol) and (*S*)-2-methyl-*N*-[(1*R*)-1-[3-[(4,4,5,5-tetramethyl-1,3,2-dioxaborolan-2-yl)methyl]-5-(2-thienyl)phenyl]ethyl] propane-2-sulfinamide **S7** (530 mg, 1.18 mmol) in dioxane (5 mL) and H_2O (0.5 mL) were added K_3PO_4 (754 mg, 3.55 mmol), SPhos (97.3 mg, 237 μmol) and $Pd(OAc)_2$ (26.6 mg,

118 μmol). The mixture was degassed and purged with N_2 3 times and then stirred at 60 °C for 6 h under N_2 atmosphere. LCMS showed 23% of desired MS was detected. The reaction mixture was diluted with water (20 mL) and was extracted with EtOAc (20 mL × 3). The combined organic layers were washed by brine and dried over sodium sulfate. Then the mixture was concentrated under reduced pressure to give a residue. The residue was purified by flash silica gel chromatography (ISCO®; 12.0 g SepaFlash® Silica Flash Column, Eluent of 0~100% EtOAc/Petroleum ether gradient @ 60 mL/min). The residue was purified by prep-HPLC (column : CD01-Phenomenex luna C18 150*25mm* 10μM; mobile phase: [H_2O (0.225% FA)-ACN]; gradient: 38%-68% B over 11.0 min) to give (*S*)-2-methyl-*N*-[(1*R*)-1-[3-[(1-methylpyrazol-4-yl)methyl]-5-(2-thienyl)phenyl]ethyl] propane-2-sulfinamide (100 mg, 249 μmol, 21.0% yield) as a yellow oil, which was de-protected by following general procedure G to give (1*R*)-1-[3-[(1-methylpyrazol-4-yl)methyl]-5-(2-thienyl)phenyl]ethanamine **S8** (82.0 mg, 234 μmol, 93.8% yield, 95.1% purity, HCl) as a light green solid. t_R = 2.573 min, ^{1}H NMR (400 MHz, DMSO-d_6) δ = 8.44 (s, 3H), 7.67 (s, 1H), 7.57 (d, *J* = 5.2 Hz, 1H), 7.55-7.49 (m, 3H), 7.30 (s, 2H), 7.19-7.13 (m, 1H), 4.46-4.33 (m, 1H), 3.80 (s, 2H), 3.77 (s, 3H), 1.52 (d, *J* = 6.8 Hz, 3H). LCMS: 298.0 $[M+H]^+$.

Synthesis of (*R*)-1-(3-bromo-5-iodophenyl)ethan-1-amine hydrochloride (S11)

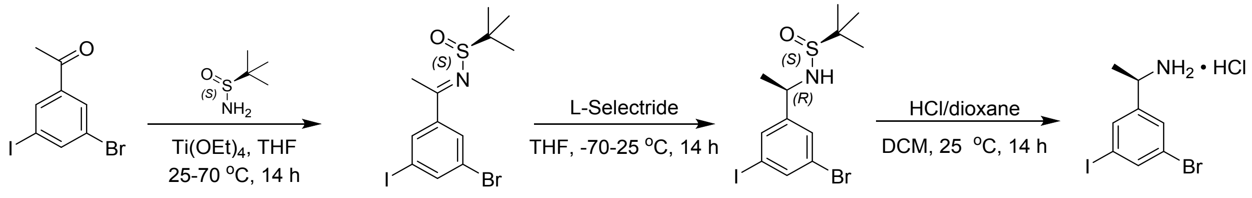

S9 **S10** **S11**

Key intermediate (*R*)-1-(3-bromo-5-iodophenyl)ethan-1-amine hydrogen chloride **S11** (2.40 g, 82% yield, 98% purity, HCl) was prepared as a white solid, following general

procedure D and G. ^{1}H NMR (400 MHz, METHANOL-D_4) δ 7.99 (d, J = 1.6 Hz, 1H), 7.85 (t, J = 1.4 Hz, 1H), 7.69 (t, J = 1.6 Hz, 1H), 4.44 (q, J = 6.9 Hz, 1H), 1.60 (d, J = 6.9 Hz, 3H). LCMS: $[M+H]^+$ 325.9

Synthesis of 2-methyl-5-(6-methyl-3,6-diazabicyclo[3.1.1]heptan-3-yl)benzoic acid (S14):

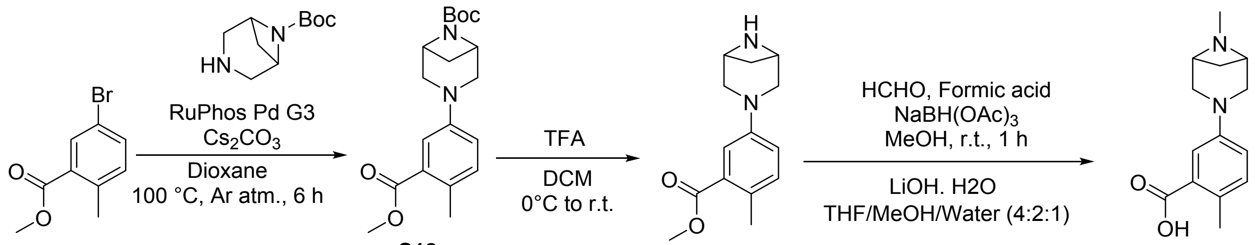

To a solution of methyl 5-bromo-2-methylbenzoate (0.231 g, 1.01 mmol, 1 equiv.) in 1,4-Dioxane (5 mL) was added tert-butyl 3,6-diazabicyclo[3.1.1]heptane-6-carboxylate (0.240 g, 1.21 mmol, 1.2 equiv.) and Cs_2CO_3 (0.98 g, 3.02 mmol, 3 equiv.) at room temperature. The reaction mixture was purged with Ar gas for 15 min followed by addition of RuPhos Pd G3 (84.1 mg, 101 µmol, 0.1 equiv.). The reaction mixture was stirred for 6 h at 100 °C. On completion the reaction mixture was diluted with water (5 mL) and extracted with EtOAc (3 x 5 mL). The organic layer was combined, dried over sodium sulfate and evaporated to give crude mixture. Purification by silica gel chromatography (ISCO®; 12 g SepaFlash® Silica Flash Column, Eluent of 5 - 100% EtOAc in Hexane gradient @ 25 mL/min) afforded *tert*-butyl 3-(3-(methoxycarbonyl)-4-methylphenyl)-3,6-diazabicyclo[3.1.1]heptane-6-carboxylate **S12** (0.287 g, 82 %) as pale-yellow oil.

S12 de-protected by following general procedure G to give methyl 5-(3,6-diazabicyclo[3.1.1]heptan-3-yl)-2-methylbenzoate **S13** (177 mg, 90 %). **S13** was involved in reductive amination (as mentioned in general procedure E) and followed by ester

hydrolysis (as mentioned in general procedure H) resulted 2-methyl-5-(6-methyl-3,6-diazabicyclo[3.1.1]heptan-3-yl)benzoic acid **S14** (0.15 g, 89%). LCMS: $[M+H]^+$ 247.2.

Synthesis of *N*-((*R*)-1-(3-bromo-5-((1-methyl-1*H*-pyrazol-4-yl)ethynyl)phenyl)ethyl)-2-methyl-5-(6-methyl-3,6-diazabicyclo[3.1.1]heptan-3-yl)benzamide (S16):

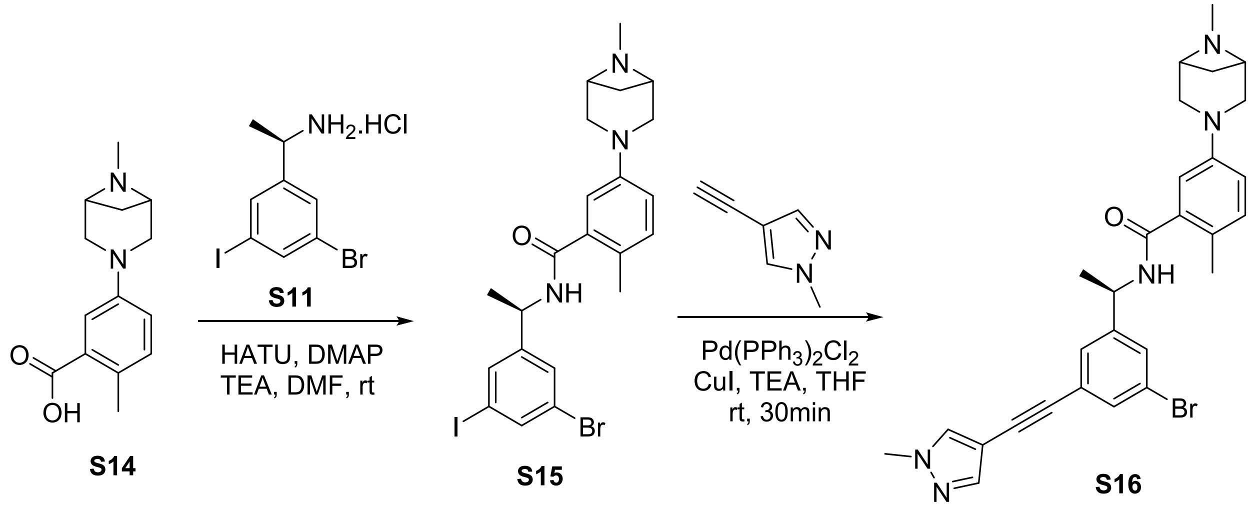

2-methyl-5-(6-methyl-3,6-diazabicyclo[3.1.1]heptan-3-yl)benzoic acid **S14** (869.5 mg, 3.53 mmol, 1.0 equiv.) was coupled with (*R*)-1-(3-bromo-5-iodophenyl)ethan-1-amine hydrogen chloride **S11** (1.28 g, 3.53 mmol, 1.0 equiv., HCl), following general procedure F to give compound *N*-((*R*)-1-(3-bromo-5-iodophenyl)ethyl)-2-methyl-5-(6-methyl-3,6-diazabicyclo[3.1.1]heptan-3-yl)benzamide **S15** (1.60 g, 82% yield) as a yellow oil.

To a dried schlenk flask equipped with a stir bar was charged with *N*-((*R*)-1-(3-bromo-5-iodophenyl)ethyl)-2-methyl-5-(6-methyl-3,6-diazabicyclo[3.1.1]heptan-3-yl)benzamide **S15** (1.63 g, 2.941 mmol, 1.0 equiv.), 4-ethynyl-1-methyl-1H-pyrazole (0.343g, 3.235 mmol, 1.1 equiv.) in THF at room temperature. Next, the schlenk tube was evacuated and filled with nitrogen (three cycles), followed by freshly distilled Et_3N (595 mg, 5.882 mmol, 2.0 equiv.), $Pd(PPh_3)_2Cl_2$ (61 mg, 0.0882 mmol, 3 mol%), CuI (22 mg, 0.117 mmol, 4 mol%) were added under the N_2 atmosphere. The resulting mixture was stirred at room

temperature for 2h. After the completion of the reaction by TLC, the reaction mixture was diluted with water and extracted with DCM. The combined organic layer was dried over Na_2SO_4, filtered, and concentrated on giving the crude material. The crude material was purified by column chromatography using DCM and methanol (10%) as the eluent gave pale yellow solid *N*-((*R*)-1-(3-bromo-5-((1-methyl-1*H*-pyrazol-4-yl)ethynyl)phenyl)ethyl)-2-methyl-5-(6-methyl-3,6-diazabicyclo[3.1.1]heptan-3-yl)benzamide **S16** in 88% (1.378 g). LCMS: $[M+H]^+$ 532.2.

Synthesis of (*R*)-*N*-(1-(3-bromo-5-((1-methyl-1*H*-pyrazol-4-yl)ethynyl)phenyl)ethyl)-2-methyl-5-(4-methylpiperazin-1-yl)benzamide (S18)

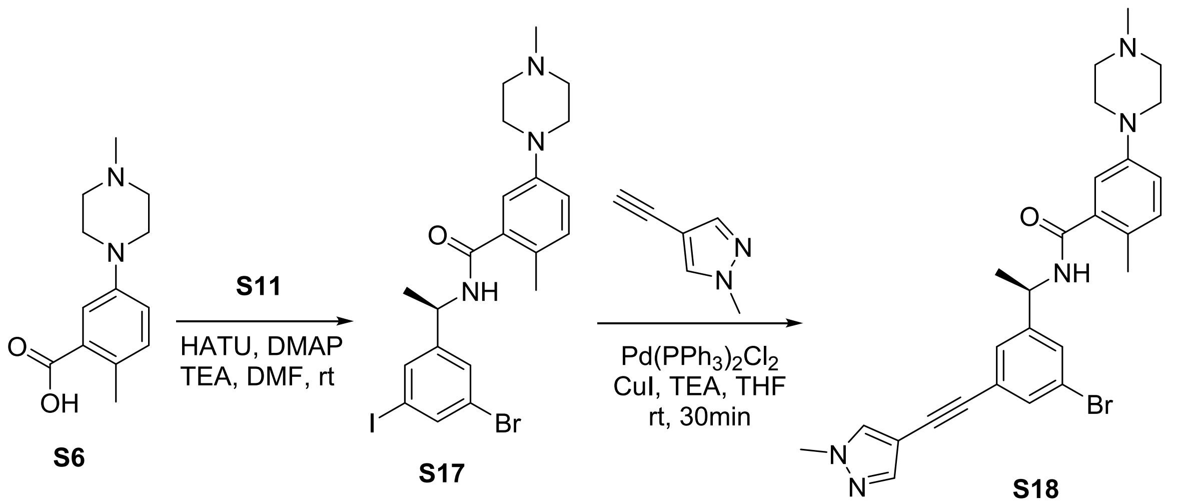

Key intermediate (*R*)-1-(3-bromo-5-iodophenyl)ethan-1-amine hydrogen chloride **S11** was coupled with **S6**, following general procedure F to give compound (*R*)-*N*-(1-(3-bromo-5-iodophenyl)ethyl)-2-methyl-5-(4-methylpiperazin-1-yl)benzamide **S17** (1.646 g, 86% yield) as a yellow oil. To a dried schlenk flask equipped with a stir bar was charged with (*R*)-*N*-(1-(3-bromo-5-iodophenyl)ethyl)-2-methyl-5-(4-methylpiperazin-1-yl)benzamide **S17** (1.595 g, 2.941 mmol, 1.0 equiv.), 4-ethynyl-1-methyl-1H-pyrazole (0.343g, 3.235 mmol, 1.1 equiv.) in THF at room temperature. Next, the Schlenk tube was evacuated

and filled with nitrogen (three cycles), followed by freshly distilled Et_3N (595 mg, 5.882 mmol, 2.0 equiv.), $Pd(PPh_3)_2Cl_2$ (61 mg, 0.0882 mmol, 3 mol%), CuI (22 mg, 0.117 mmol, 4 mol%) were added under the N_2 atmosphere. The resulting mixture was stirred at room temperature for 2h. After the completion of the reaction by TLC, the reaction mixture was diluted with water and extracted with DCM. The combined organic layer was dried over Na_2SO_4, filtered, and concentrated on giving the crude material. The crude material was purified by column chromatography using DCM and methanol (10%) as the eluent gave pale yellow solid (*R*)-*N*-(1-(3-bromo-5-((1-methyl-1*H*-pyrazol-4-yl)ethynyl)phenyl)ethyl)-2-methyl-5-(4-methylpiperazin-1-yl)benzamide **S18** (1362 mg, yield 89%). LCMS: $[M+H]^+$ 520.2.

Synthesis of 1-(3-bromo-5-(1-methyl-1*H*-pyrazol-4-yl)phenyl)ethan-1-one (S1b)

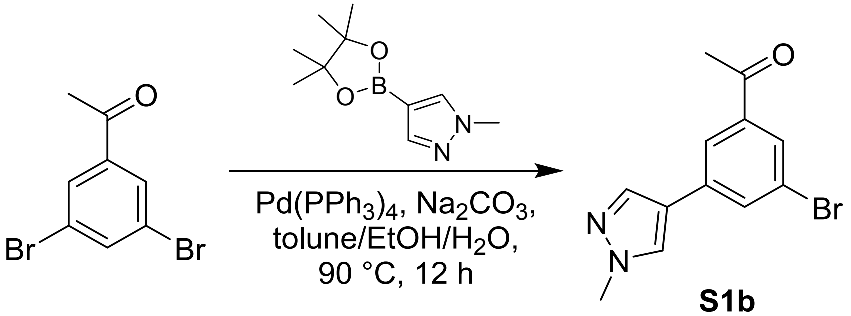

S1b

S1b was prepared by following same procedure for preparation **S1a,** to give compound 1-[3-bromo-5-(1-methylpyrazol-4-yl)phenyl]ethanone **S1b** (2.76 g, 59.1% yield) as a pale yellow solid. ^1H NMR (400 MHz, DMSO-d_6) δ 8.35 (s, 1H), 8.07 (s, 1H), 7.99-8.05 (m, 2H), 7.81-7.85 (m, 1H), 3.87 (s, 3H), 2.62 (s, 3H).

Synthesis of (*S*)-*N*-[(1*R*)-1-[3-bromo-5-(1-methylpyrazol-4-yl)phenyl]ethyl]-2-methyl-propane-2-sulfinamide (S3b)

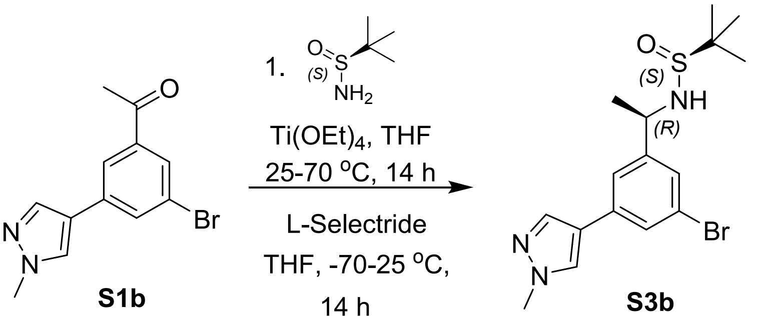

The intermediate (*S*)-*N*-[(1*R*)-1-[3-bromo-5-(1-methylpyrazol-4-yl)phenyl]ethyl]-2-methyl-propane-2-sulfinamide **S3b** (3.10 g, 89.4% yield) as a yellow oil, following general procedure D. ^{1}H NMR (400 MHz, $CDCl_3$) δ 7.70 (s, 1H), 7.59 (s, 1H), 7.47 (d, *J* = 1.6 Hz, 1H), 7.32 (s, 1H), 7.28 (t, *J* = 1.6 Hz, 1H), 4.47-4.57 (m, 1H), 3.91 (s, 3H), 1.50 (d, *J* = 6.8 Hz, 3H), 1.19 (s, 9H).

Synthesis of (*R*)-1-(3-bromo-5-(1-methyl-1*H*-pyrazol-4-yl)phenyl)ethan-1-amine hydrochloride (S19)

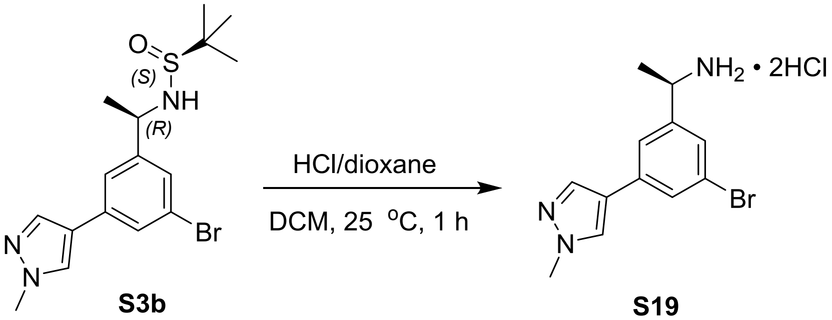

The compound (1*R*)-1-[3-bromo-5-(1-methylpyrazol-4-yl)phenyl]ethanamine **S19** (2.20 g, 76.3% yield, 98.7% purity, 2HCl) as a yellow solid, following general procedure G. ^{1}H NMR (400 MHz, DMSO-d_6) δ 8.83 (br s, 3H), 8.27 (s, 1H), 7.99 (s, 1H), 7.96 (s, 1H), 7.77 (s, 1H), 7.57 (s, 1H), 4.26-4.45 (m, 1H), 3.86 (s, 3H), 1.54 (d, *J* = 6.8 Hz, 3H). LCMS: $[M-NH_2]^+$ 263.0.

Synthesis of (*R*)-*N*-(1-(3-bromo-5-(1-methyl-1*H*-pyrazol-4-yl)phenyl)ethyl)-2-methyl-5-(4-methylpiperazin-1-yl)benzamide (S20)

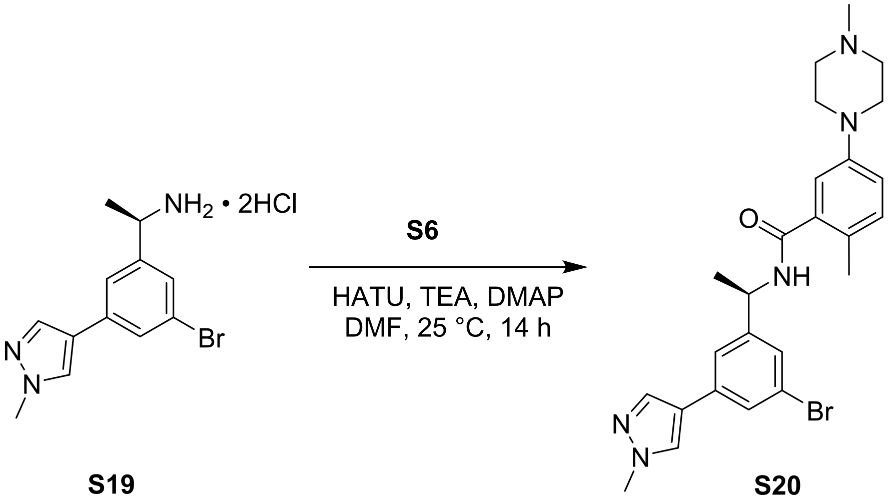

S19 **S20**

The amine **S19** was coupled with **S6**, following general procedure F to give compound *N*-[(1*R*)-1-[3-bromo-5-(1-methylpyrazol-4-yl)phenyl]ethyl]-2-methyl-5-(4-methylpiperazin-1-yl)benzamide S20 (200 mg, 71.1% yield) as a yellow oil. ^{1}H NMR (400 MHz, CHLOROFORM-*D*) δ 7.70 – 7.65 (m, 2H), 7.48 – 7.40 (m, 2H), 7.35 – 7.30 (m, 1H), 7.07 – 6.98 (m, 1H), 6.84 – 6.79 (m, 1H), 6.79 – 6.72 (m, 1H), 5.13 (q, *J* = 6.8 Hz, 1H), 3.87 (s, 3H), 3.18 – 3.12 (m, 4H), 2.86 – 2.82 (m, 4H), 2.75 (s, 3H), 2.24 (s, 3H), 1.54 (d, *J* = 7.0 Hz, 3H). LCMS: $[M+H]^+$ 498.0

Synthesis of methyl 2-methyl-5-(piperidin-4-yloxy)benzoate hydrochloride (S22)

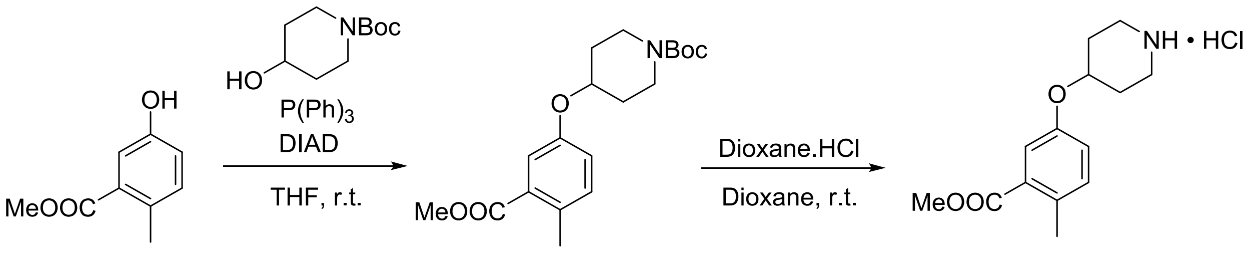

S21 **S22**

To a solution of methyl 5-hydroxy-2-methylbenzoate (0.30 g, 1.81 mmol, 1 equiv.) in THF (4 mL) was added tert-butyl 4-hydroxypiperidine-1-carboxylate (0.4 g, 1.99 mmol, 1.1 equiv.), triphenylphosphine (0.57 g, 2.17 mmol, 1.2 equiv.) and DIAD (0.46 mL, 2.35 mmol, 1.3 equiv.) at 0 °C. The reaction mixture was stirred at room temperature for 12 h. The reaction progress was monitored by TLC (EtOAc:Hexane = 1:9, UV). On reaction completion, the reaction mixture was diluted with water (10 mL) and extracted with EtOAc (3 x 10 mL). The combined organic layer was dried over sodium sulfate and evaporated to give a crude mixture. Purification by silica gel chromatography (ISCO®; 40 g SepaFlash® Silica Flash Column, Eluent of 5 - 60% EtOAc in Hexane gradient @ 60 mL/min) afforded tert-butyl 4-(3-(methoxycarbonyl)-4-methylphenoxy)piperidine-1-carboxylate **S21** (307 mg, yield 48.7 %). Boc-deprotection was done by following general procedure G to give methyl 5-(3,6-diazabicyclo[3.1.1]heptan-3-yl)-2-methylbenzoate hydrochloride **S22** as a white solid (183.5 mg, 92 %). ^{1}H NMR (400 MHz, METHANOL-D_4) δ 7.48 (d, *J* = 2.7 Hz, 1H), 7.23 (d, *J* = 8.5 Hz, 1H), 7.11 (dd, *J* = 8.4, 2.8 Hz, 1H), 4.76 – 4.67 (m, 1H), 3.87 (s, 3H), 3.45 – 3.34 (m, 2H), 3.28 – 3.18 (m, 2H), 2.48 (s, 3H), 2.24 – 2.11 (m, 2H), 2.08 – 1.96 (m, 2H).

Synthesis of 2-methyl-5-((1-methylpiperidin-4-yl)oxy)benzoic acid (S23)

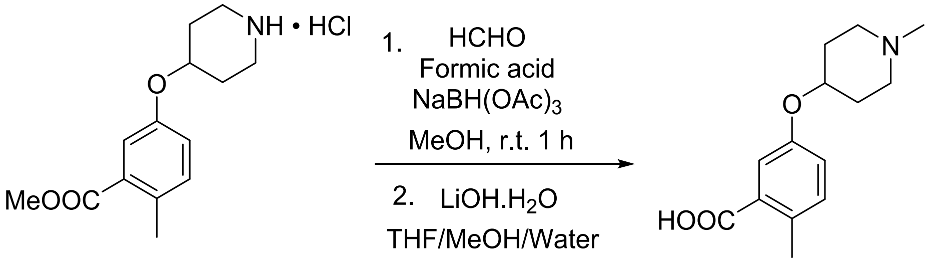

S22 **S23**

S22 (0.178 g, 0.712 mmol, 1 equiv.) was methylated to give methyl 2-methyl-5-((1-methylpiperidin-4-yl)oxy)benzoate (66 %) by following general procedure E. Subsequently, ester hydrolysis by following general procedure H to afforded 2-methyl-5-((1-methylpiperidin-4-yl)oxy)benzoic acid **S23** (150.7 mg, 88%). ^{1}H NMR (400 MHz, METHANOL-D_4) δ 7.08 – 6.98 (m, 2H), 6.81 – 6.74 (m, 1H), 4.39 – 4.34 (m, 1H), 2.72 – 2.68 (m, 2H), 2.46 – 2.30 (m, 5H), 2.28 (s, 3H), 2.05 – 1.95 (m, 2H), 1.84 – 1.74 (m, 2H). LCMS: [M+H]+ 250.2.

Synthesis of *N*-(1-(3-bromo-5-((1-methyl-1*H*-pyrazol-4-yl)ethynyl)phenyl) cyclopropyl)-2-methyl-5-(4-methylpiperazin-1-yl)benzamide (S27):

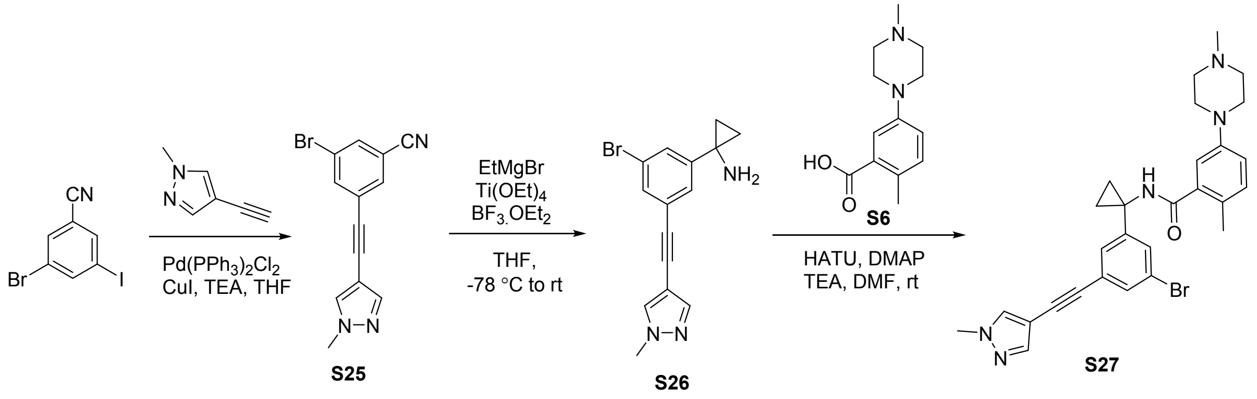

To a dried round bottom flask equipped with a stir bar was charged with 3-bromo-5-iodobenzonitrile (3.624 g, 11.764 mmol, 1.0 equiv.), 4-ethynyl-1-methyl-1H-pyrazole (1.372g, 12.94 mmol, 1.1 equiv.) in THF at room temperature. Next, round bottom flask was evacuated and filled with nitrogen (three cycles), followed by freshly distilled Et_3N (2.38g, 23.528 mmol, 2.0 equiv.), $Pd(PPh_3)_2Cl_2$ (244 mg, 0.353 mmol, 3 mol%), CuI (88 mg, 0.470 mmol, 4 mol%) were added under the N_2 atmosphere. The resulting mixture was stirred at room temperature for 2h. After the completion of the reaction by TLC, the reaction mixture was diluted with water and extracted with EA. The combined organic

layer was dried over Na_2SO_4, filtered, and concentrated on giving the crude material. The crude material was purified by column chromatography using Hexanes and EA as the eluent gave pale yellow solid 3-bromo-5-((1-methyl-1*H*-pyrazol-4-yl)ethynyl)benzonitrile **S25** in 83% (2.792g). ^{1}H NMR (400 MHz, CHLOROFORM-*D*) δ 7.84 – 7.80 (m, 1H), 7.71 – 7.63 (m, 3H), 7.62 – 7.56 (m, 1H), 3.93 (s, 3H).

To a solution of 3-bromo-5-((1-methyl-1*H*-pyrazol-4-yl)ethynyl)benzonitrile **S25** (2.741 g, 9.58 mmol, 1 equiv.) in THF (40 mL) was added tetraisopropoxytitanium (2.99 g, 3.20 mL, 1.1 equiv., 10.54 mmol) with stirring at -78 °C, followed by dropwise addition of ethylmagnesium bromide (2.81 g, 2.2 equiv., 7.03 mL, 3 M in Et_2O, 21.08 mmol). The reaction was stirred at -78 °C for 0.5 h and slowly brought to room temperature. To the reaction mixture was added $BF_3.OEt_2$ (5.667 g, 5.059 mL, 2 equiv., 48% Wt, 19.16 mmol) and stirred for 0.5 h. The reaction progress was monitored by TLC and on completion the reaction mixture was slowly added to satd. $NaHCO_3$ (25 mL) and extracted with EtOAc (3 x 25 mL). The combined organic layer was washed with water and dried over sodium sulfate. Purification by silica gel chromatography (SiO_2 column, 40 g, 5 - 80% EtOAc/Hex)) afforded 1-(3-bromo-5-((1-methyl-1*H*-pyrazol-4-yl)ethynyl)phenyl) cyclopropan-1-amine **S26** (1.06 g, 35 %) as pale-yellow liquid. This amine **S26** was coupled with acid **S6** following general procedure F to give *N*-(1-(3-bromo-5-((1-methyl-1*H*-pyrazol-4-yl)ethynyl)phenyl)cyclopropyl)-2-methyl-5-(4-methylpiperazin-1-yl)benzamide **S27** (0.77 g, 82% yield) as a yellow oil. LCMS: [M+H]+ 532.2.

References

(1) Borg, G.; Cogan, D. A.; Ellman, J. A. One-pot asymmetric synthesis of tert-butanesulfinyl-protected amines from ketones by the in situ reduction of tert-butanesulfinyl ketimines. *Tetrahedron Letters* **1999**, *40* (37), 6709-6712. DOI: https://doi.org/10.1016/S0040-4039(99)01351-9.

(2) Xiao, J.; Xiu, W.; Zhang, Q.; Curto, J. M.; Jia, M.; Wang, M.; Zhao, M.; Dirico, K. J.; White, O.; Zhou, D.; et al. C(sp2)–C(sp3) Suzuki–Miyaura Cross-Coupling Using gem-Bis(boronates). *The Journal of Organic Chemistry* **2025**, *90* (40), 14316-14321. DOI: 10.1021/acs.joc.5c01605.

NMR Spectra

Compound **9**

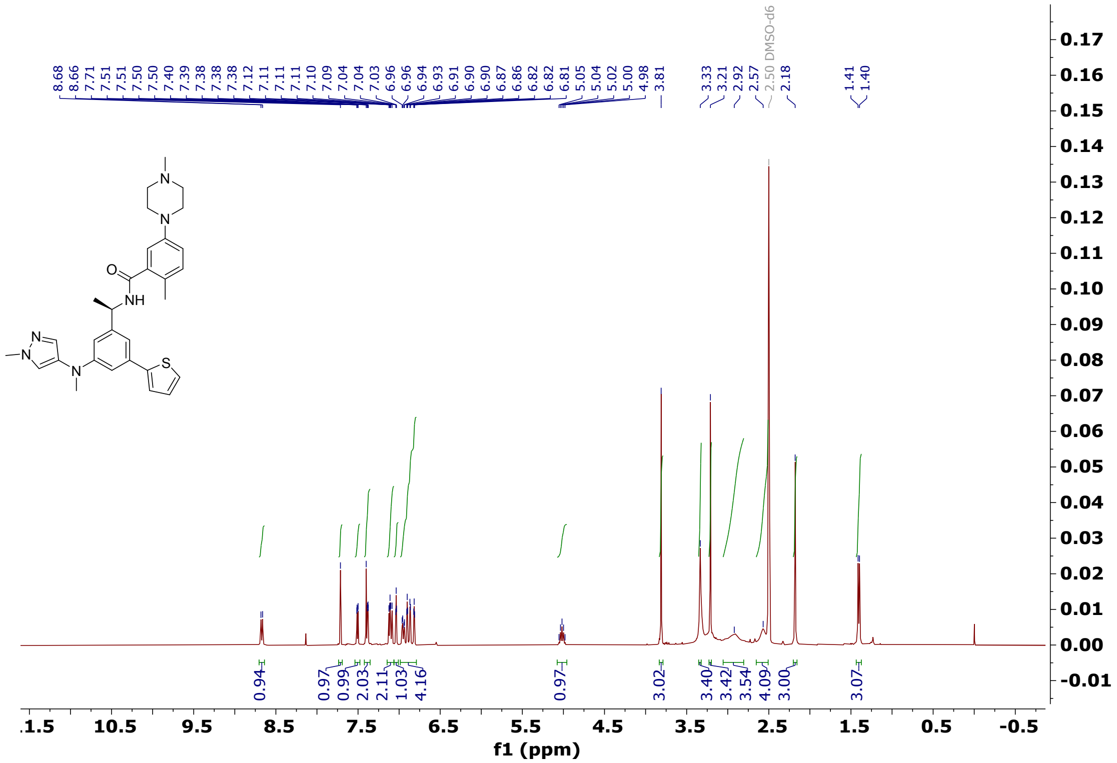

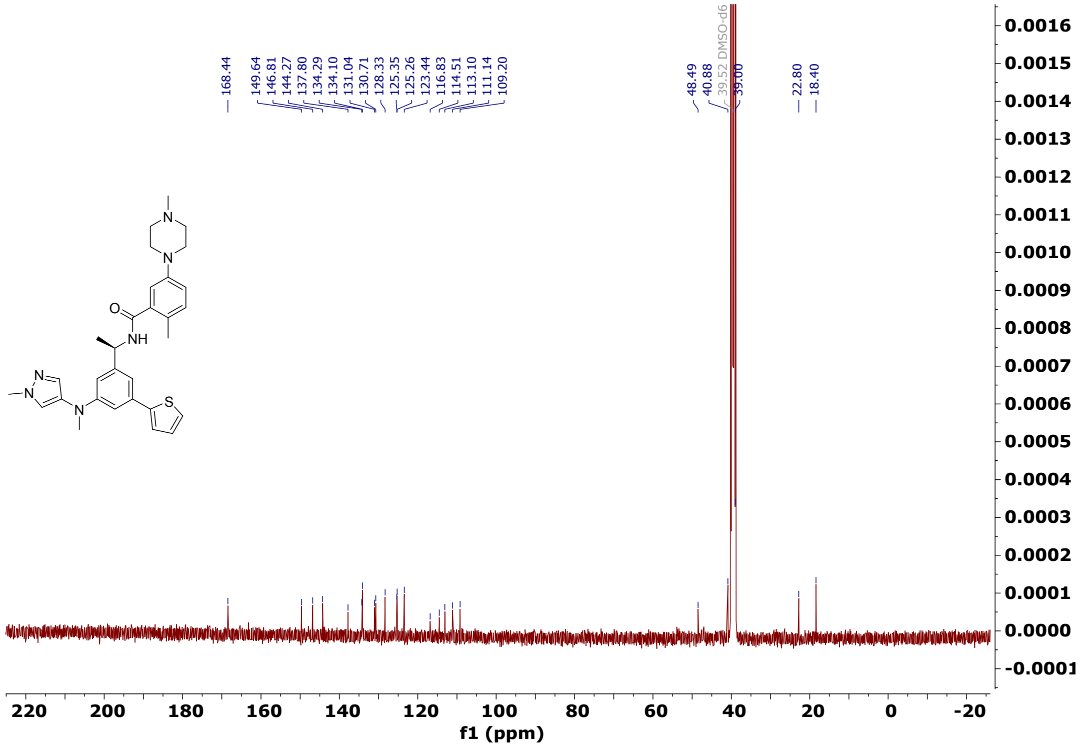

Compound **10**

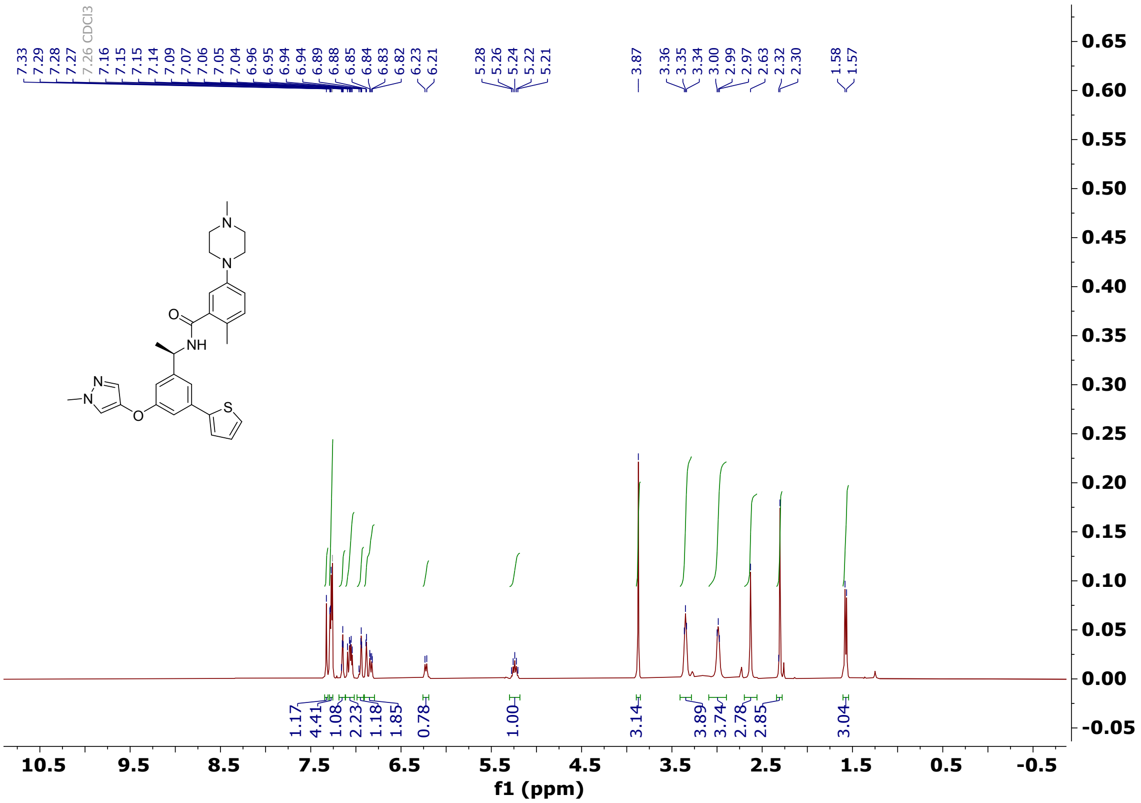

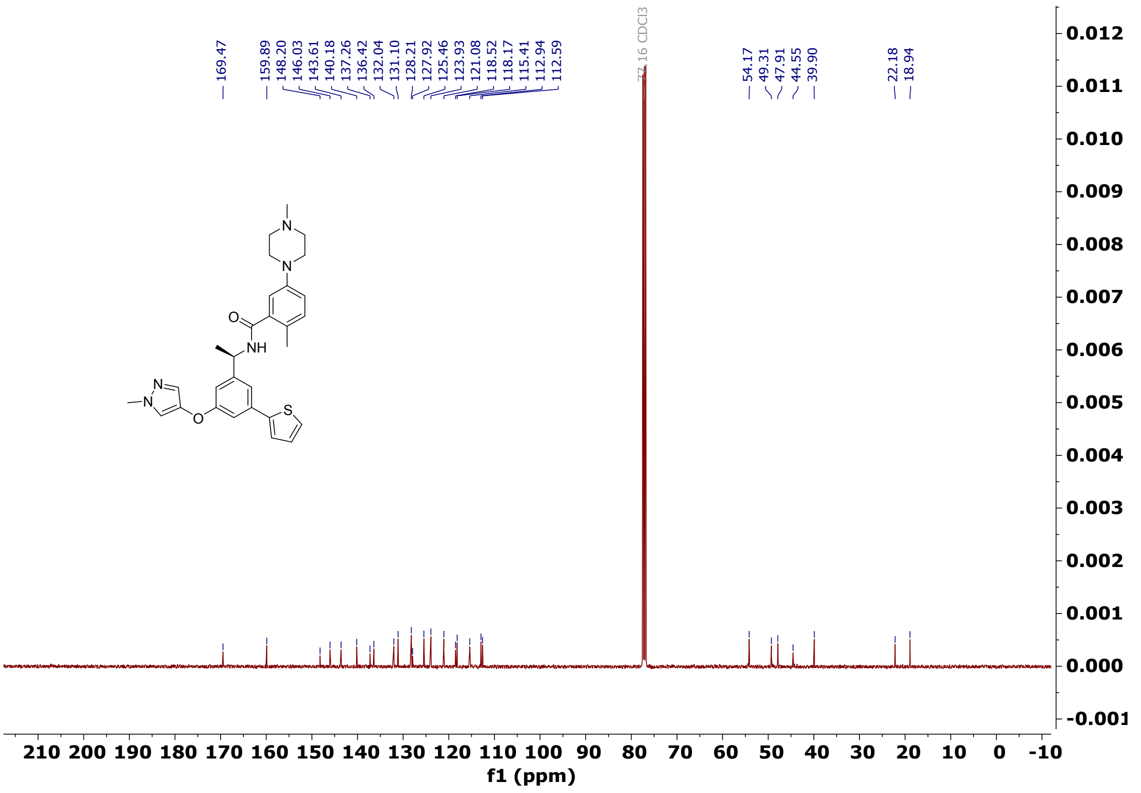

Compound **11**

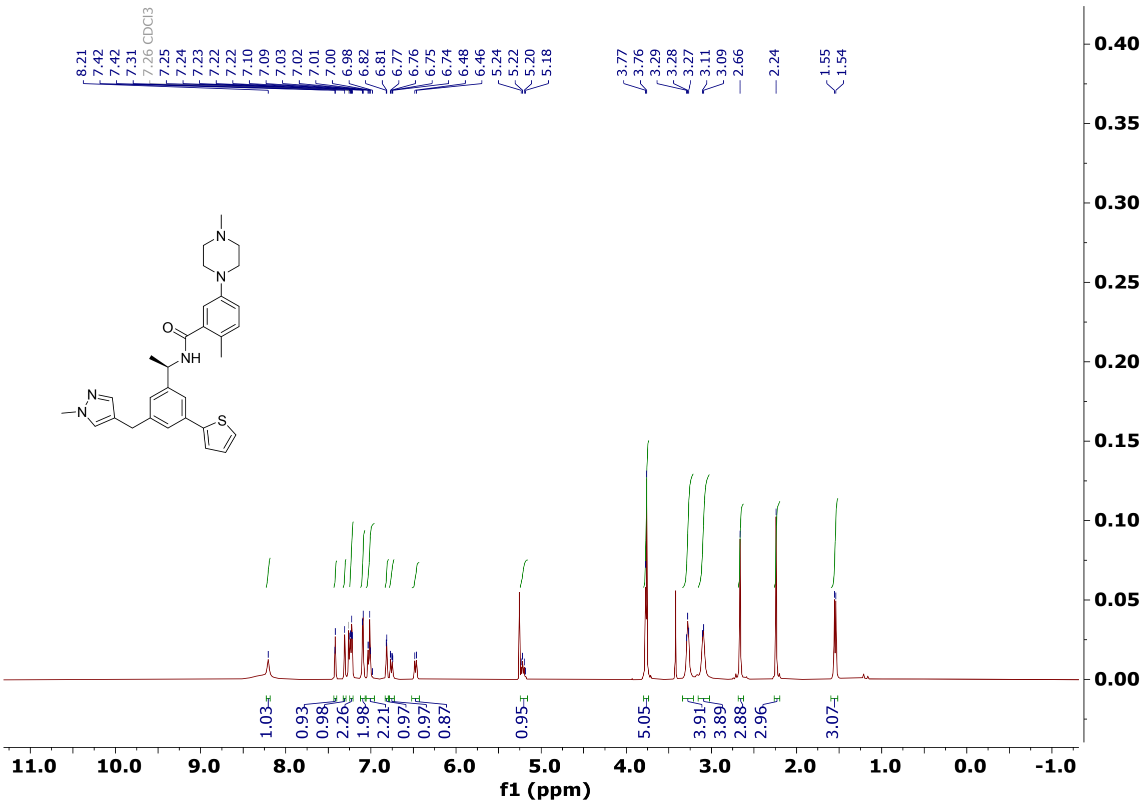
8.21
7.42
7.42
7.31
7.26 CDCl3
7.25
7.24
7.23
7.22
7.22
7.10
7.09
7.03
7.02
7.01
7.00
6.98
6.82
6.81
6.77
6.76
6.75
6.74
6.48
6.46
5.24
5.22
5.20
5.18
3.77
3.76
3.29
3.28
3.27
3.11
3.09
2.66
2.24
1.55
1.54
1.03
0.93
0.98
2.26
1.98
2.21
0.97
0.97
0.87
0.95
5.05
3.91
3.89
2.88
2.96
3.07
f1 (ppm)

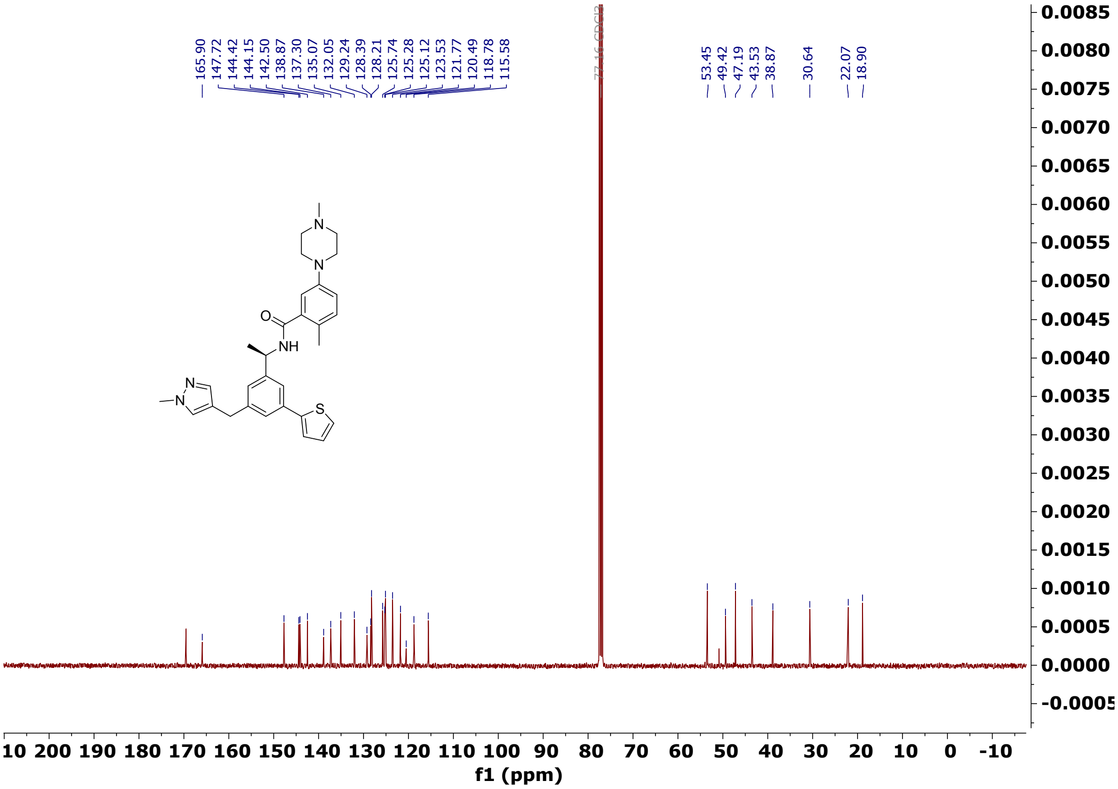
165.90
147.72
144.42
144.15
142.50
138.87
137.30
135.07
132.05
129.24
128.39
128.21
125.74
125.28
125.12
123.53
121.77
120.49
118.78
115.58
53.45
49.42
47.19
43.53
38.87
30.64
22.07
18.90
f1 (ppm)

Compound **12**

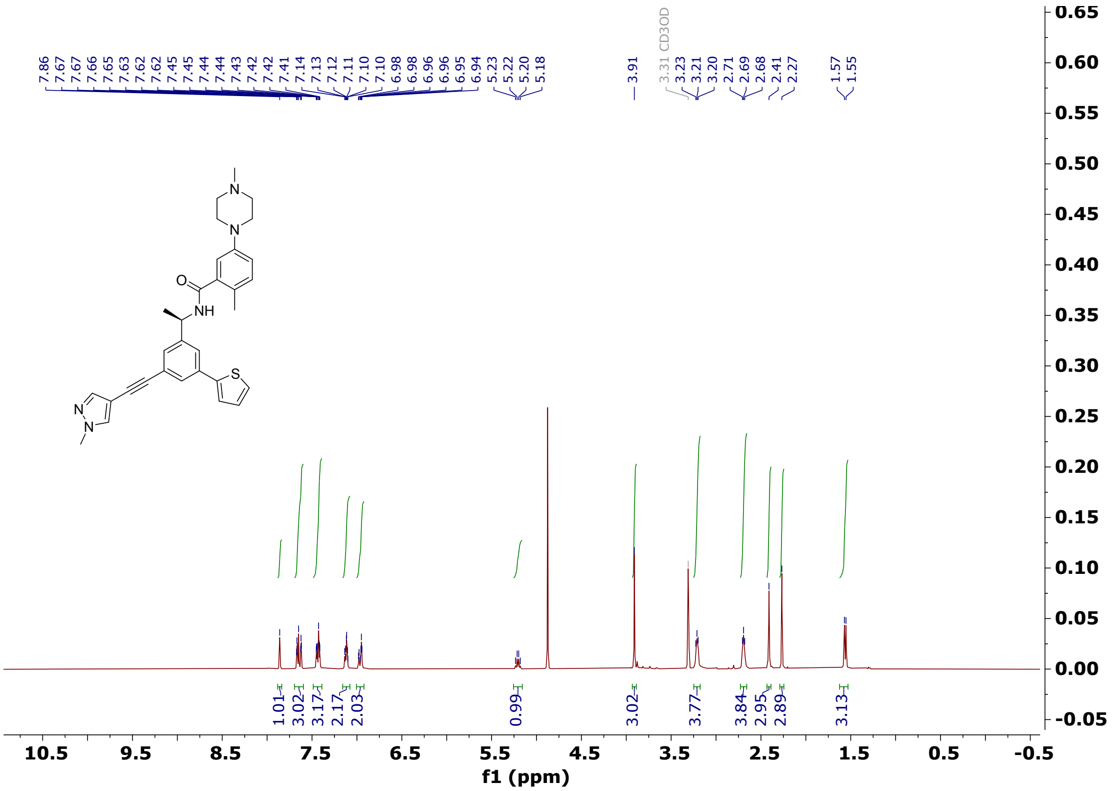
7.86 7.67 7.67 7.66 7.65 7.63 7.62 7.62 7.45 7.45 7.44 7.44 7.43 7.42 7.41 7.14 7.13 7.12 7.11 7.10 6.98 6.98 6.96 6.96 6.95 6.94 5.23 5.22 5.20 5.18
3.91
3.31 CD3OD
3.23 3.21 3.20 2.71 2.69 2.68 2.41 2.27
1.57 1.55
N
N
O
NH
N
N
S
1.01 3.02 3.17 2.17 2.03 0.99 3.02 3.77 3.84 2.95 2.89 3.13
f1 (ppm)

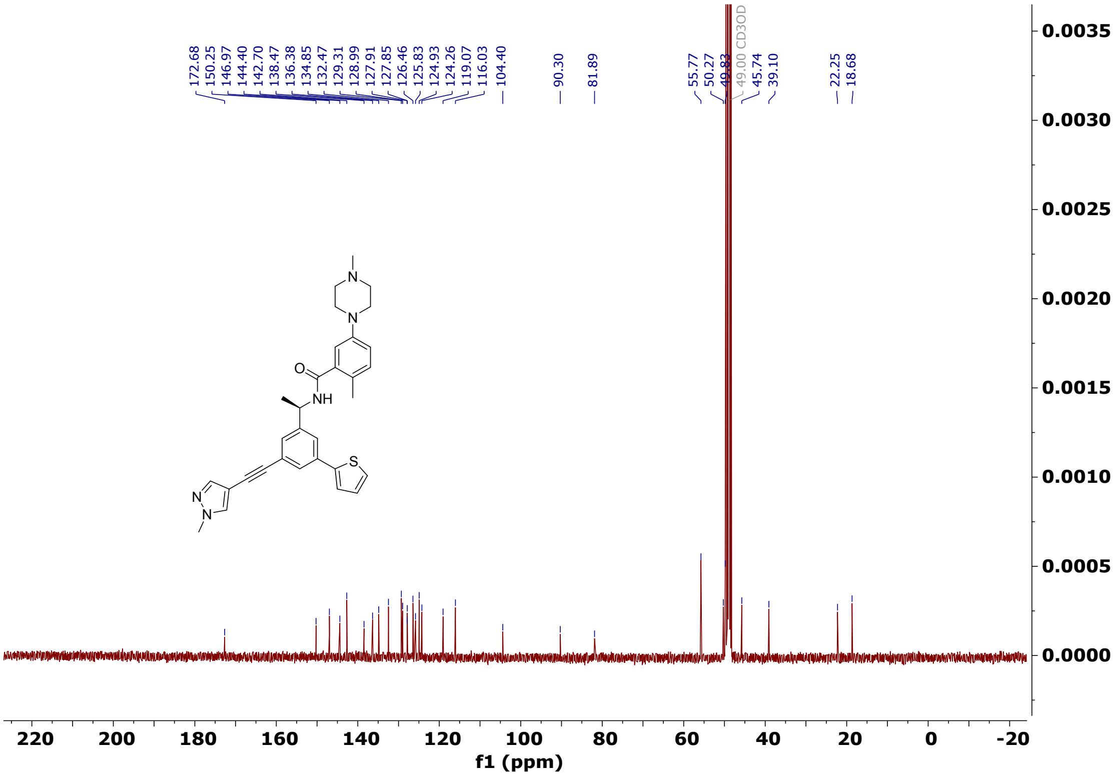
172.68 150.25 146.97 144.40 142.70 138.47 136.38 134.85 132.47 129.31 128.99 127.91 127.85 126.46 125.83 124.93 124.26 119.07 116.03 104.40
90.30
81.89
55.77 50.27 49.83
49.00 CD3OD
45.74 39.10
22.25 18.68
N
N
O
NH
N
N
S
f1 (ppm)

Compound **13**

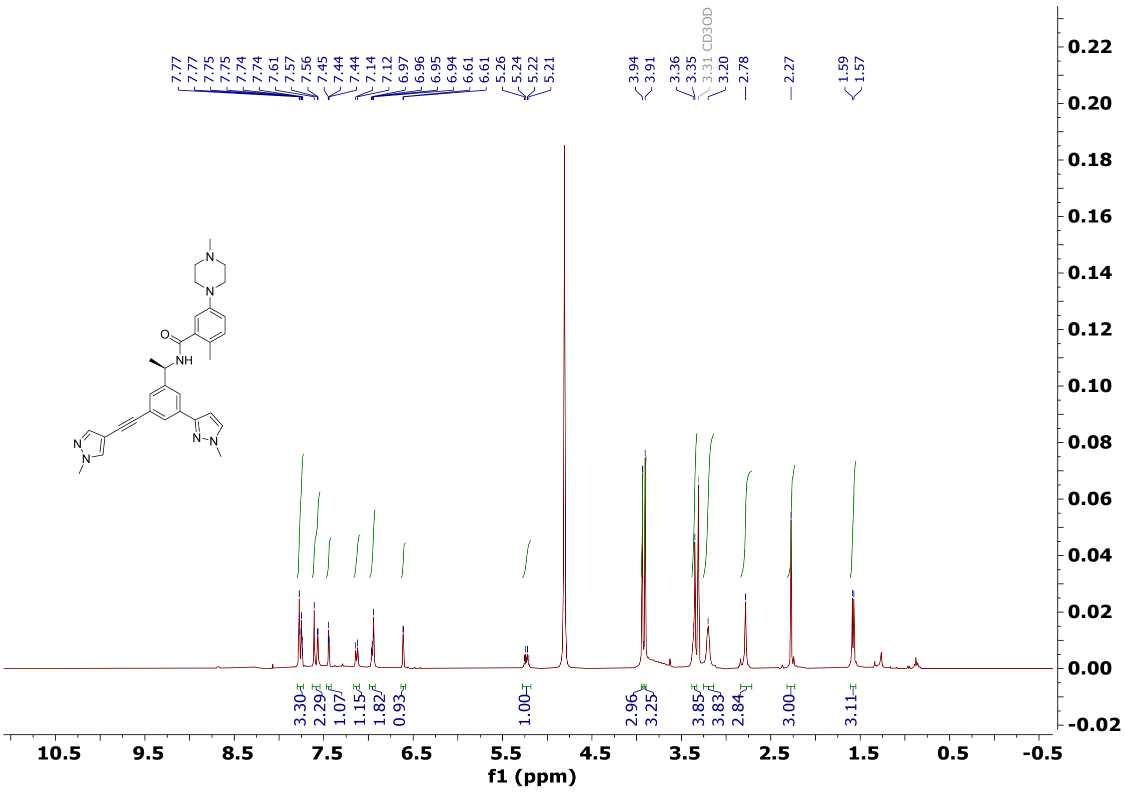

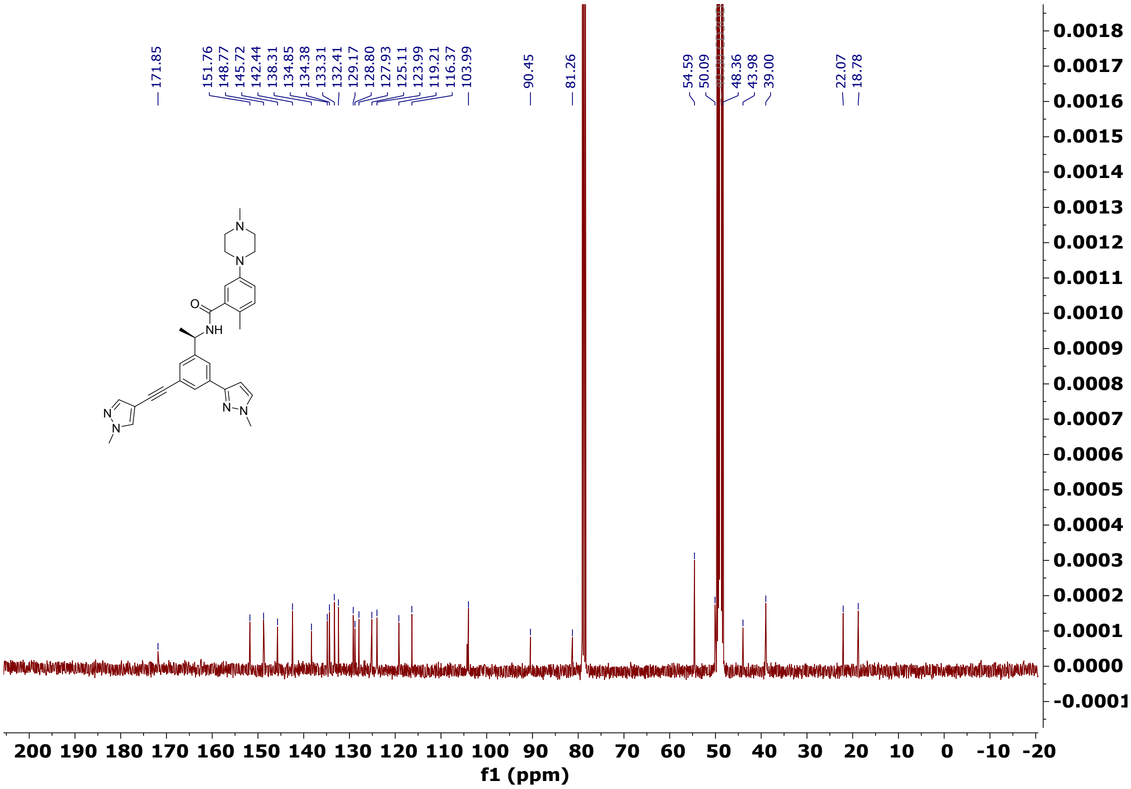

Compound **14**

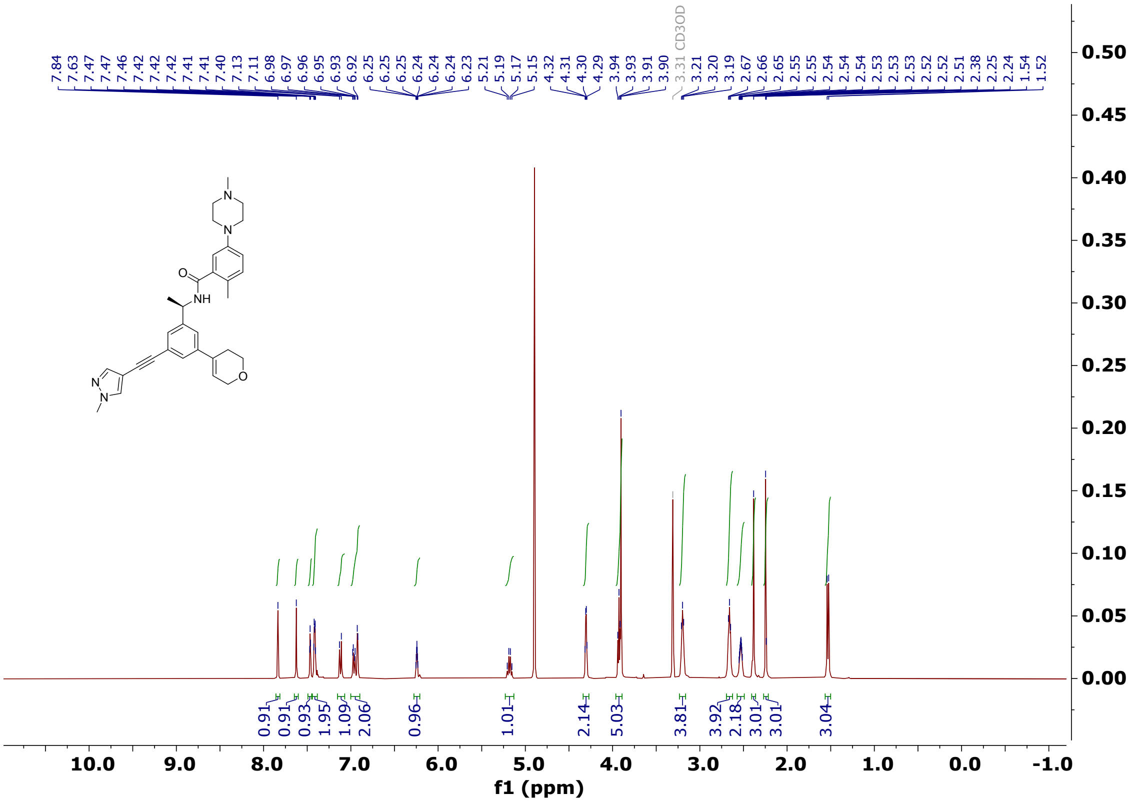

7.84 7.63 7.47 7.47 7.46 7.42 7.42 7.42 7.41 7.41 7.40 7.13 7.11 6.98 6.97 6.96 6.95 6.93 6.92 6.25 6.25 6.25 6.24 6.24 6.24 6.23 5.21 5.19 5.17 5.15 4.32 4.31 4.30 4.29 3.94 3.93 3.91 3.90 3.31 CD3OD 3.21 3.20 3.19 2.67 2.66 2.65 2.55 2.55 2.54 2.54 2.54 2.53 2.53 2.53 2.52 2.52 2.51 2.38 2.25 2.24 1.54 1.52
0.91 0.91 0.93 1.95 1.09 2.06 0.96 1.01 2.14 5.03 3.81 3.92 2.18 3.01 3.01 3.04
f1 (ppm)

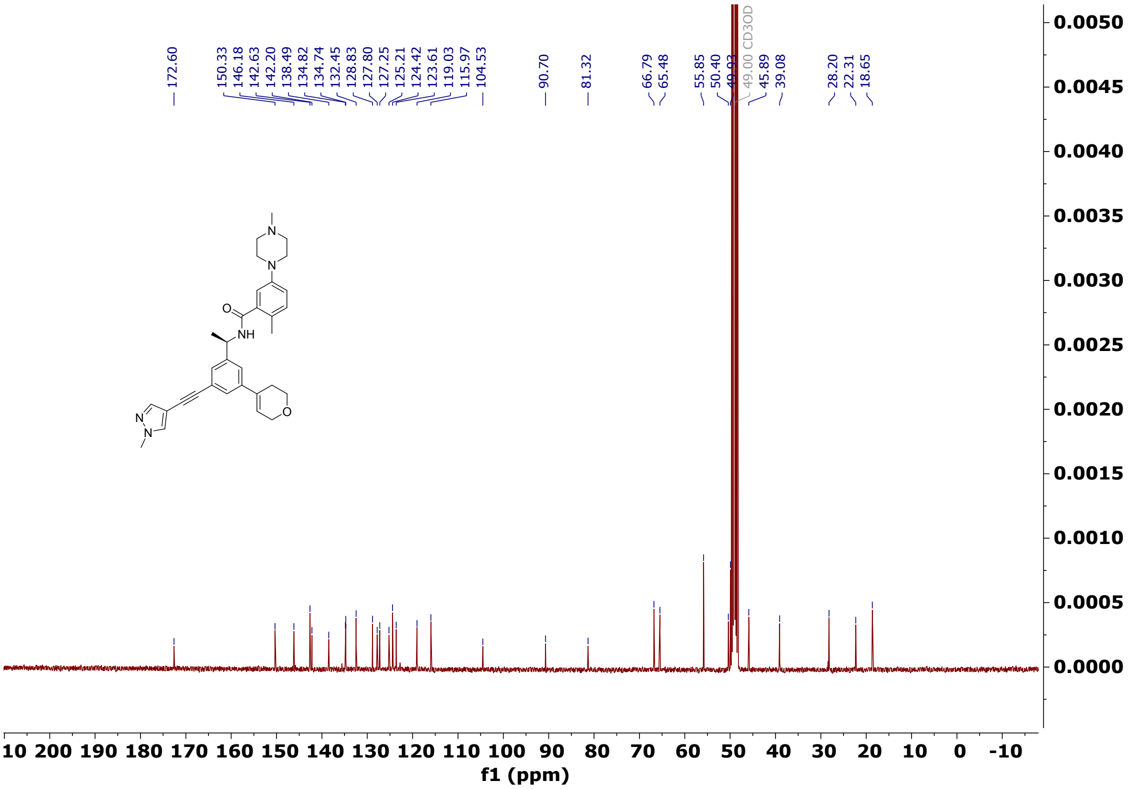

172.60 150.33 146.18 142.63 142.20 138.49 134.82 134.74 132.45 128.83 127.80 127.25 125.21 124.42 123.61 119.03 115.97 104.53 90.70 81.32 66.79 65.48 55.85 50.40 49.00 CD3OD 45.89 39.08 28.20 22.31 18.65
f1 (ppm)

Compound **15**

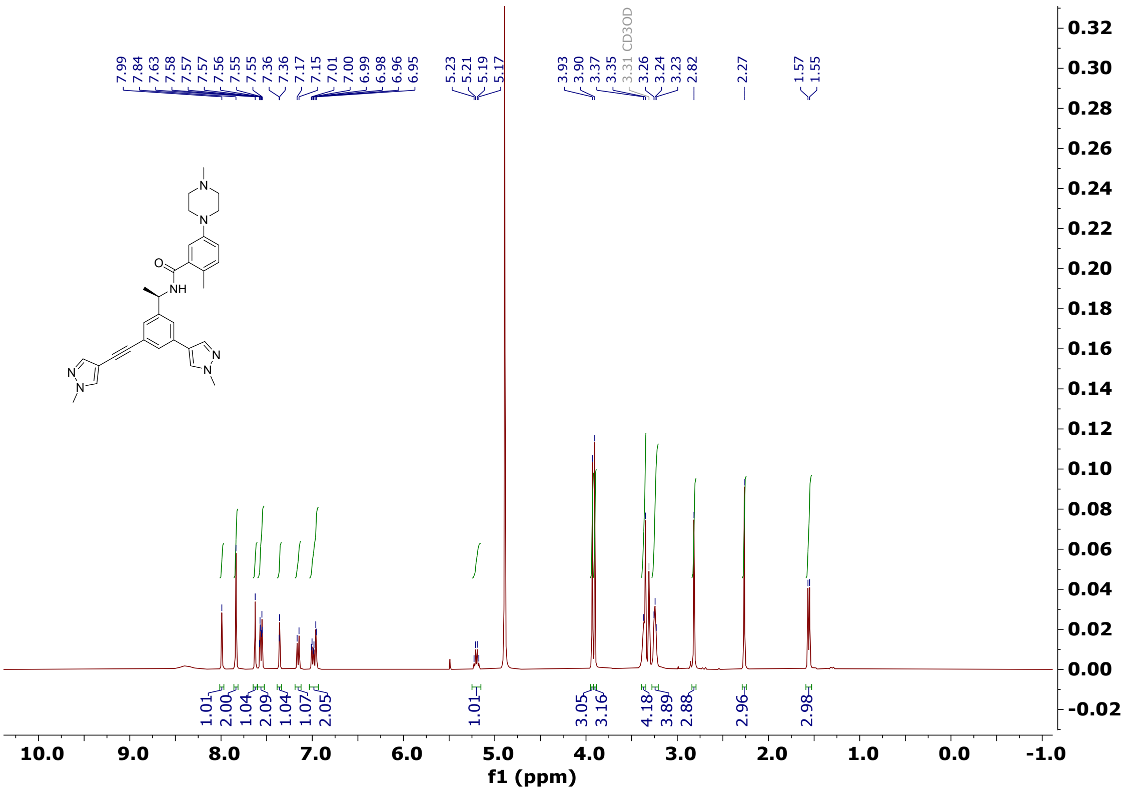

7.99
7.84
7.63
7.58
7.57
7.57
7.56
7.55
7.55
7.36
7.36
7.17
7.15
7.01
7.00
6.99
6.98
6.96
6.95
5.23
5.21
5.19
5.17
3.93
3.90
3.37
3.35
3.31 CD3OD
3.26
3.24
3.23
2.82
2.27
1.57
1.55
1.01
2.00
1.04
2.09
1.04
1.07
2.05
1.01
3.05
3.16
4.18
3.89
2.88
2.96
2.98
f1 (ppm)

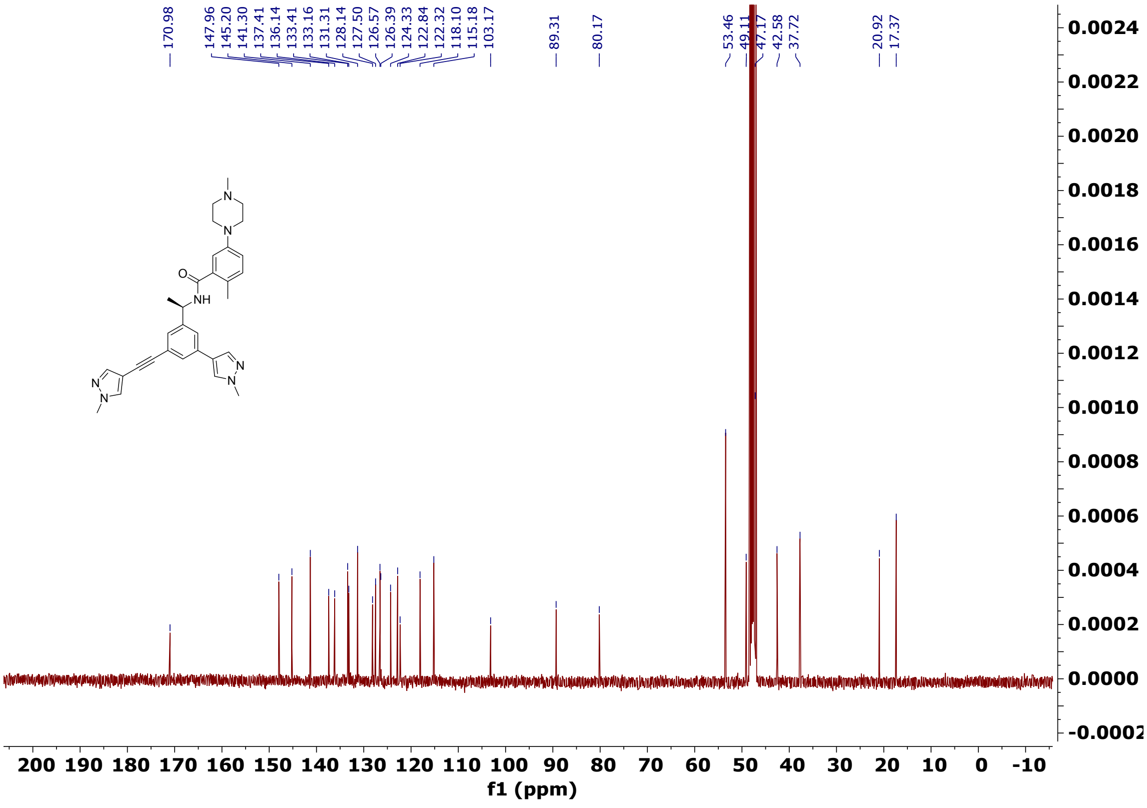

170.98
147.96
145.20
141.30
137.41
136.14
133.41
133.16
131.31
128.14
127.50
126.57
126.39
124.33
122.84
122.32
118.10
115.18
103.17
89.31
80.17
53.46
49.11
47.17
42.58
37.72
20.92
17.37
f1 (ppm)

Compound **16**

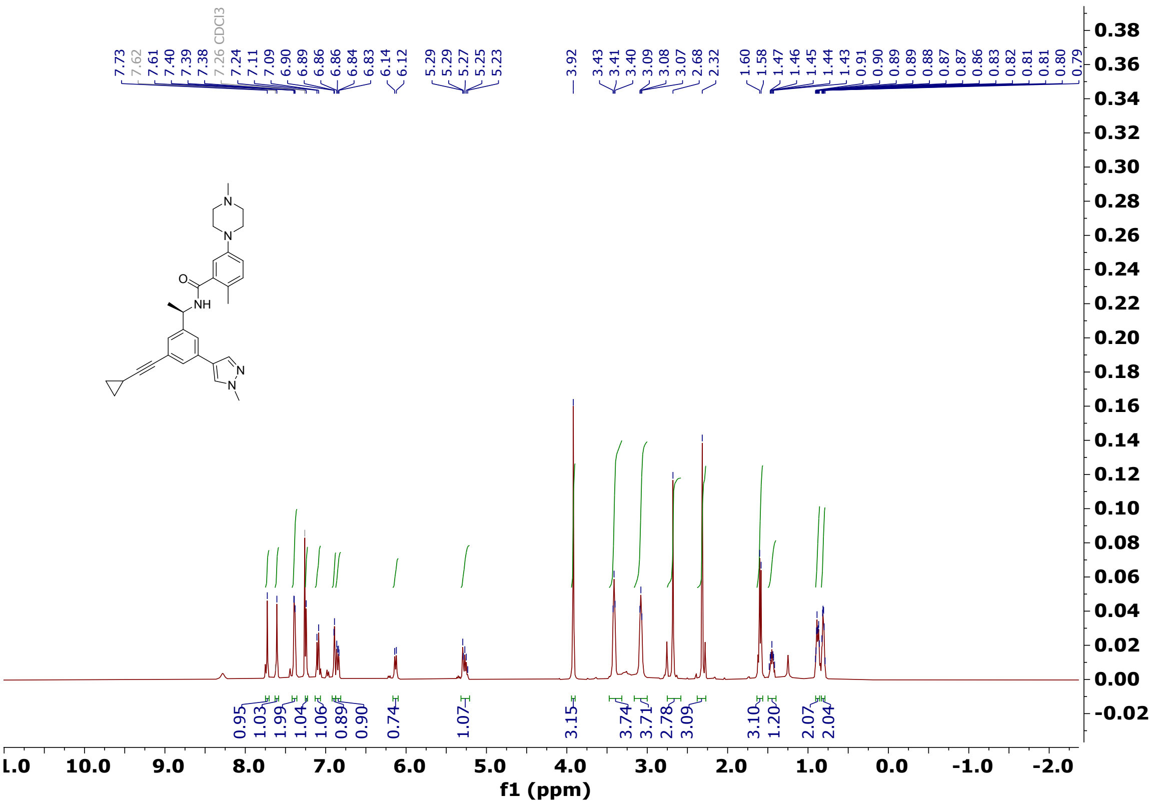

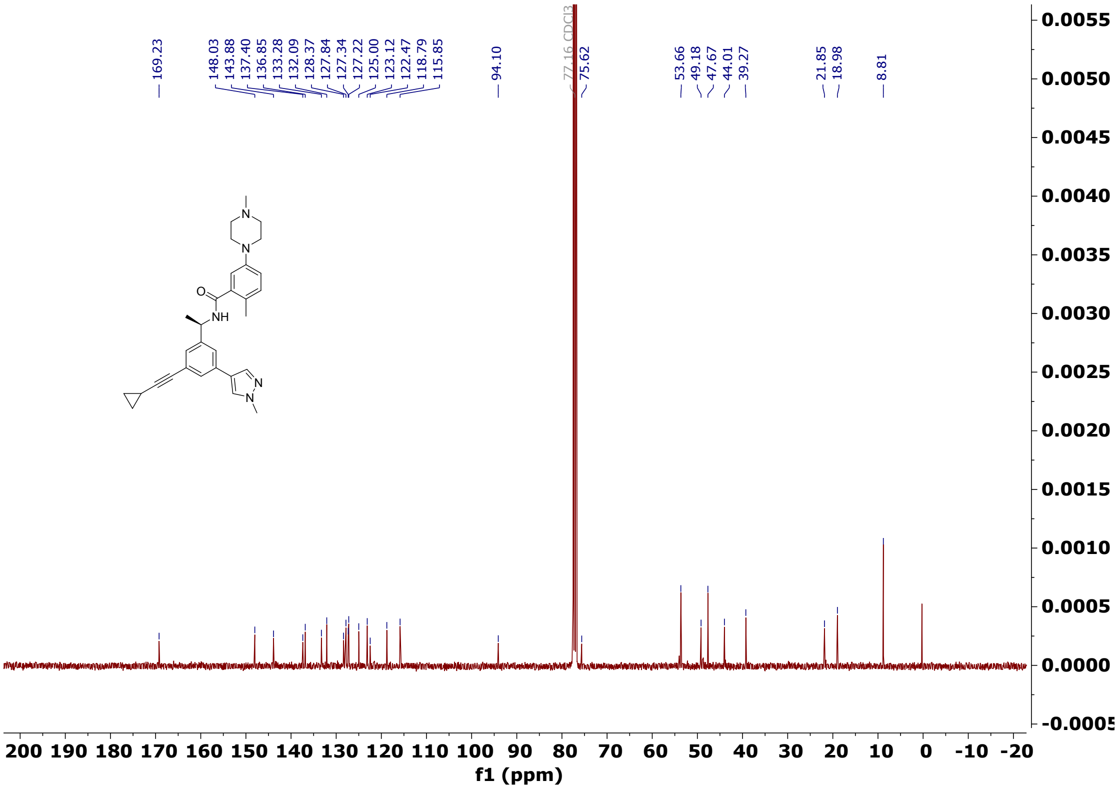

Compound **17**

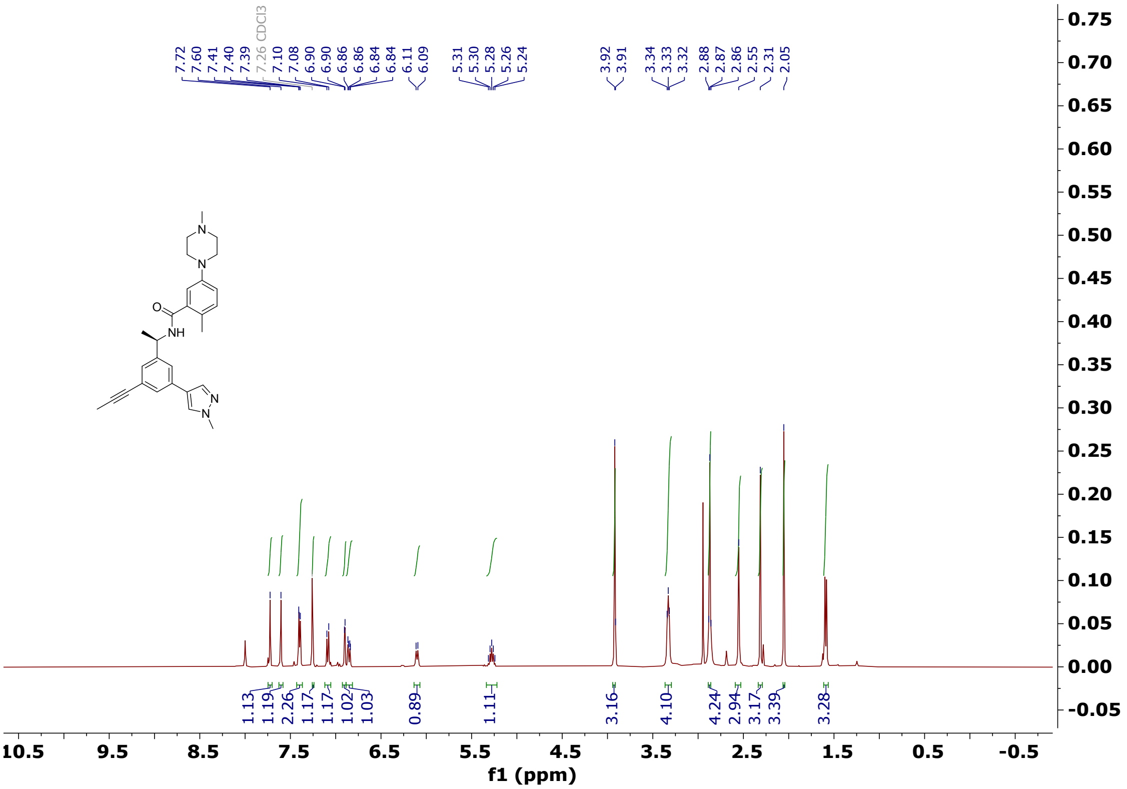
7.72 7.60 7.41 7.40 7.39 7.26 CDCl3 7.10 7.08 6.90 6.90 6.86 6.86 6.84 6.84 6.11 6.09 5.31 5.30 5.28 5.26 5.24 3.92 3.91 3.34 3.33 3.32 2.88 2.87 2.86 2.55 2.31 2.05
1.13 1.19 2.26 1.17 1.17 1.02 1.03 0.89 1.11 3.16 4.10 4.24 2.94 3.17 3.39 3.28
f1 (ppm)

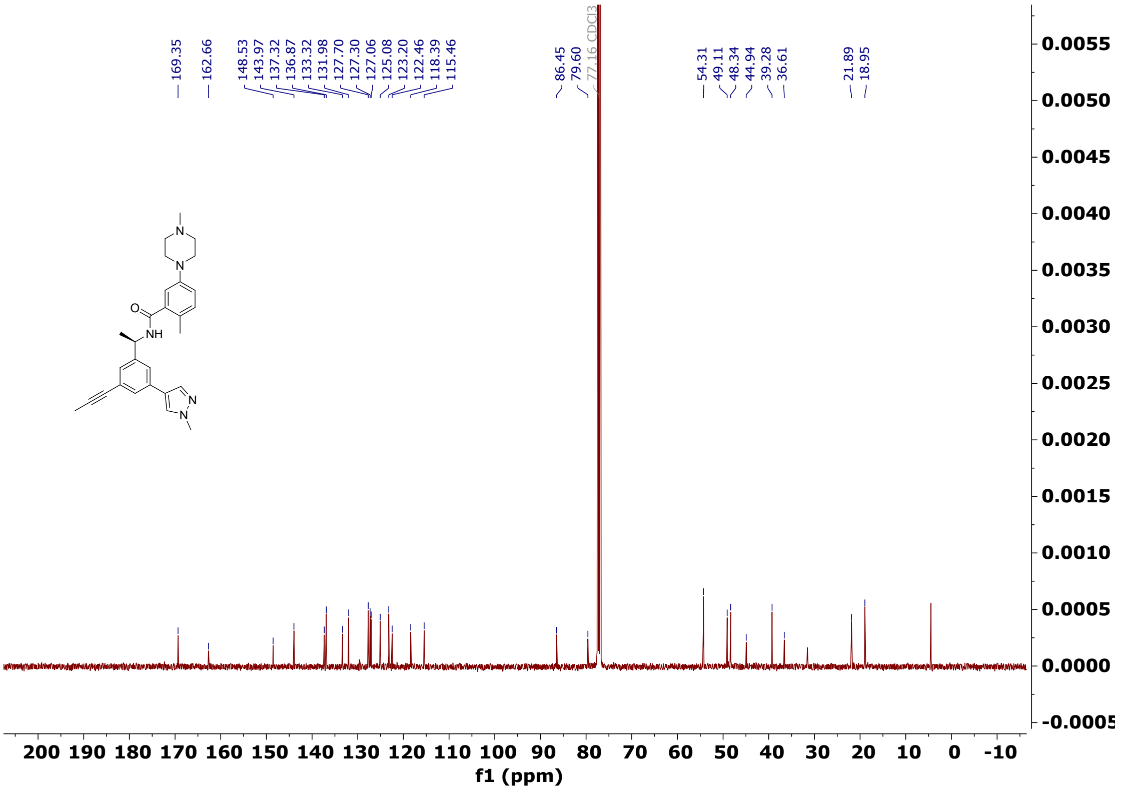
169.35 162.66 148.53 143.97 137.32 136.87 133.32 131.98 127.70 127.30 127.06 125.08 123.20 122.46 118.39 115.46 86.45 79.60 77.16 CDCl3 54.31 49.11 48.34 44.94 39.28 36.61 21.89 18.95
f1 (ppm)

Compound **18**

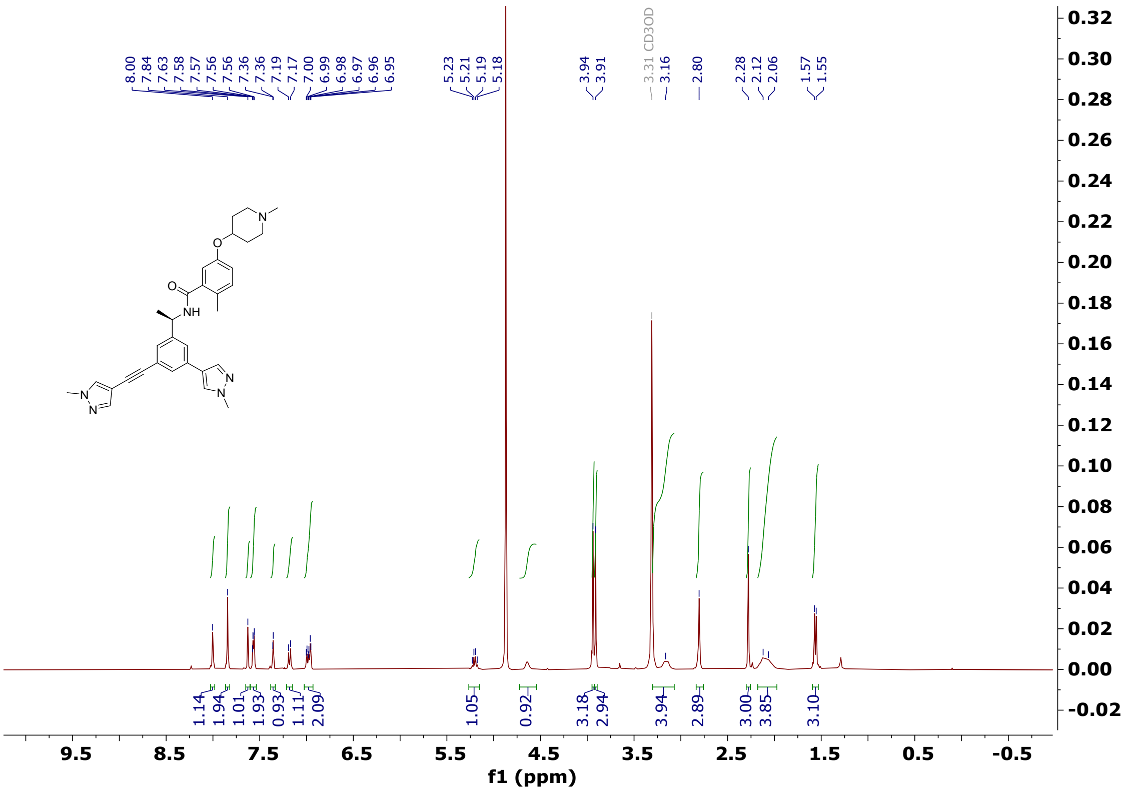

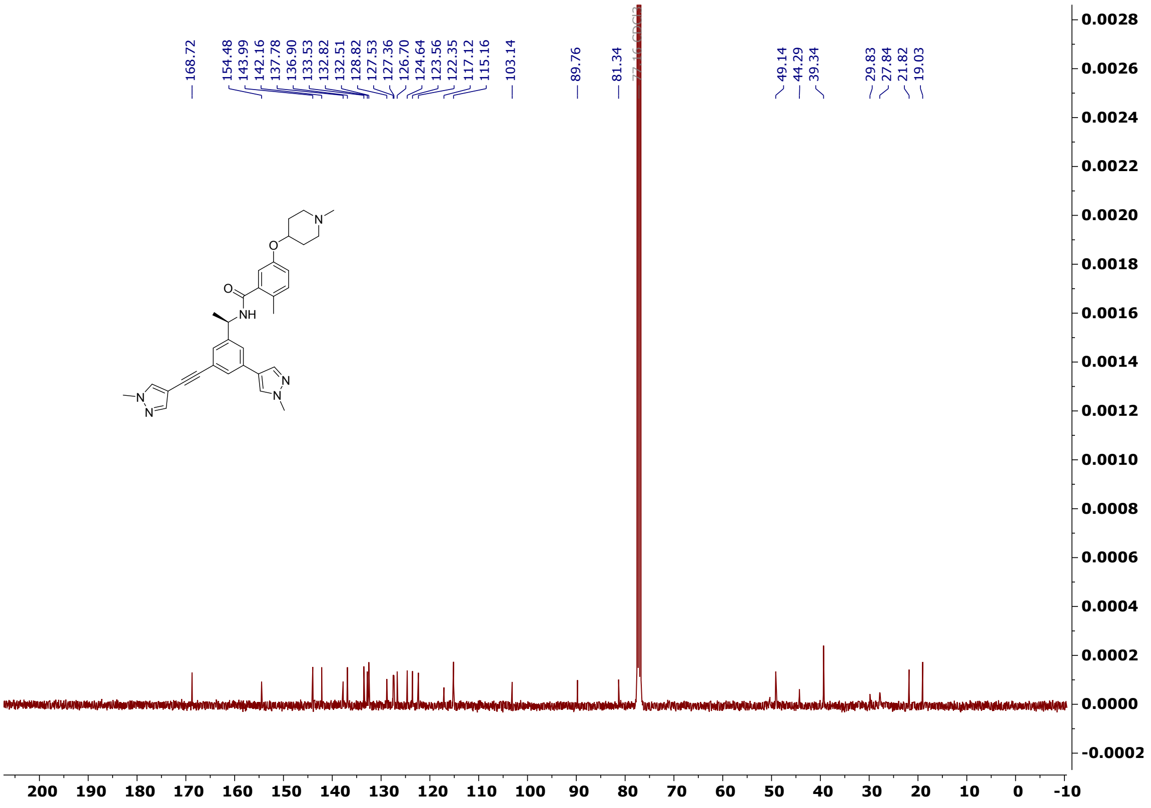

Compound **19**

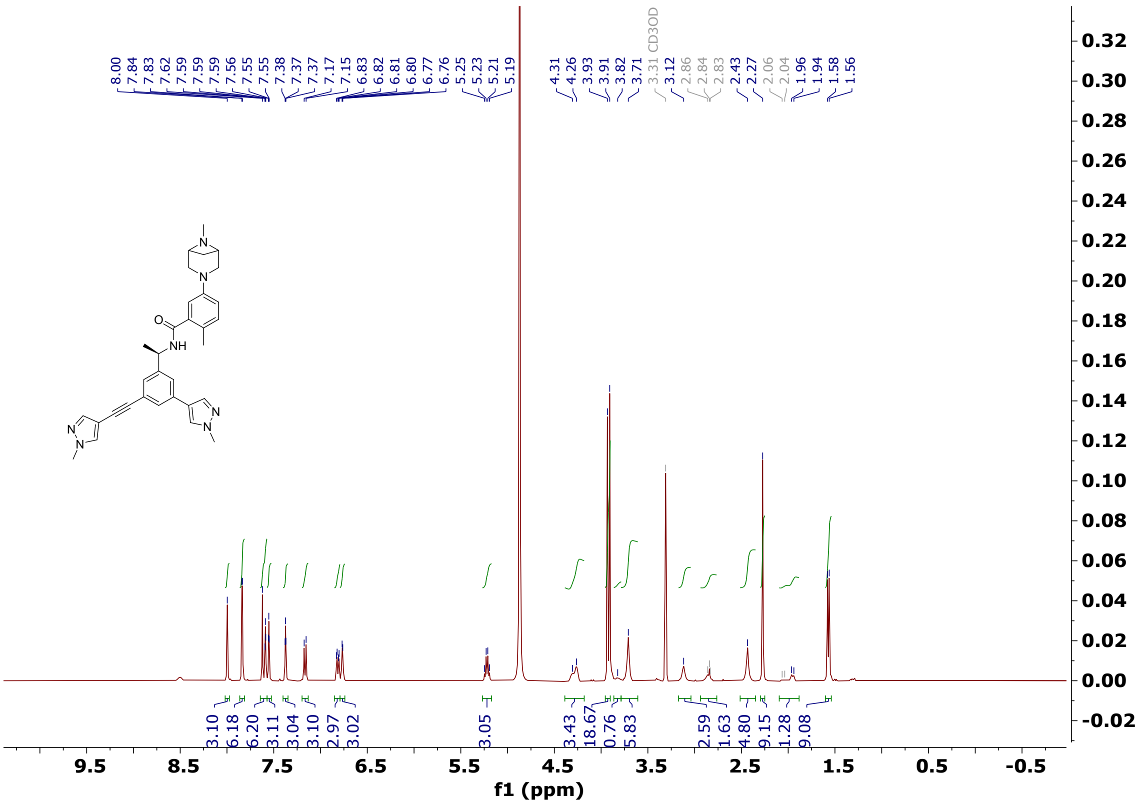
8.00 7.84 7.83 7.62 7.59 7.59 7.59 7.56 7.55 7.55 7.38 7.37 7.37 7.17 7.15 6.83 6.82 6.81 6.80 6.77 6.76 5.25 5.23 5.21 5.19 4.31 4.26 3.93 3.91 3.82 3.71 3.31 CD3OD 3.12 2.86 2.84 2.83 2.43 2.27 2.06 2.04 1.96 1.94 1.58 1.56
3.10 6.18 6.20 3.11 3.04 3.10 2.97 3.02 3.05 3.43 18.67 0.76 5.83 2.59 1.63 4.80 9.15 1.28 9.08
O
NH
N
f1 (ppm)

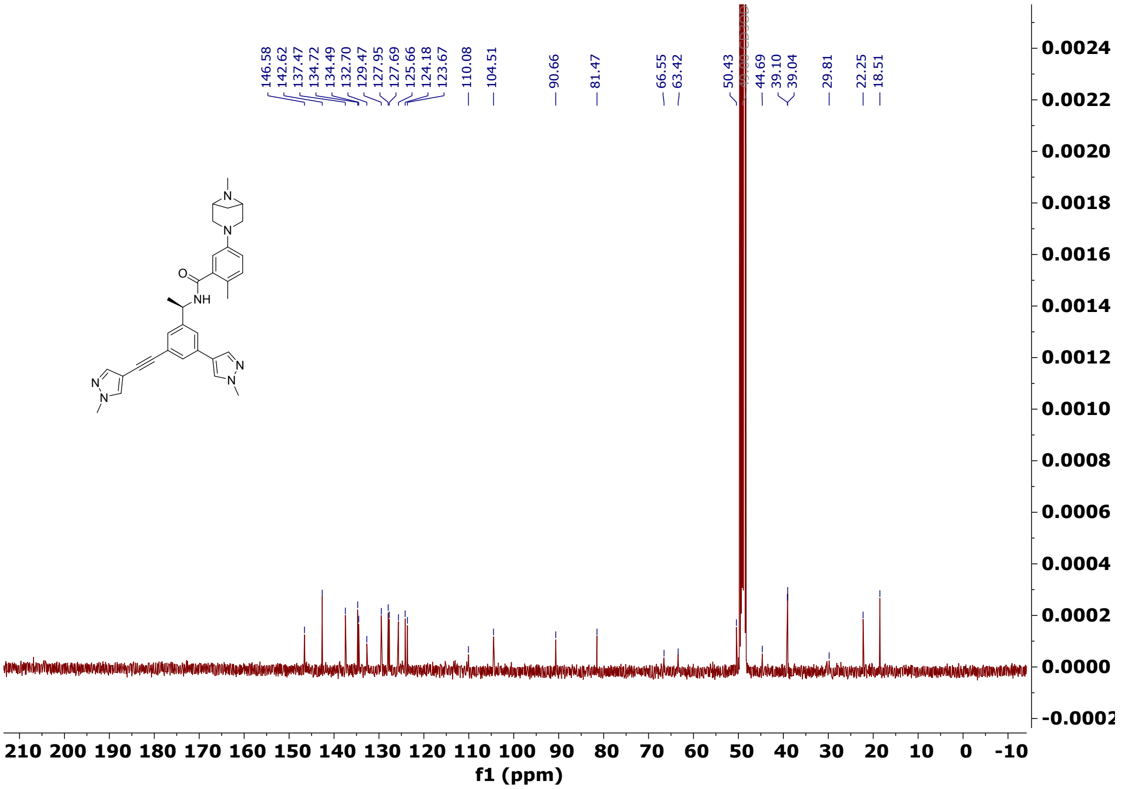
146.58 142.62 137.47 134.72 134.49 132.70 129.47 127.95 127.69 125.66 124.18 123.67 110.08 104.51 90.66 81.47 66.55 63.42 50.43 44.69 39.10 39.04 29.81 22.25 18.51
O
NH
N
f1 (ppm)

Compound **20**

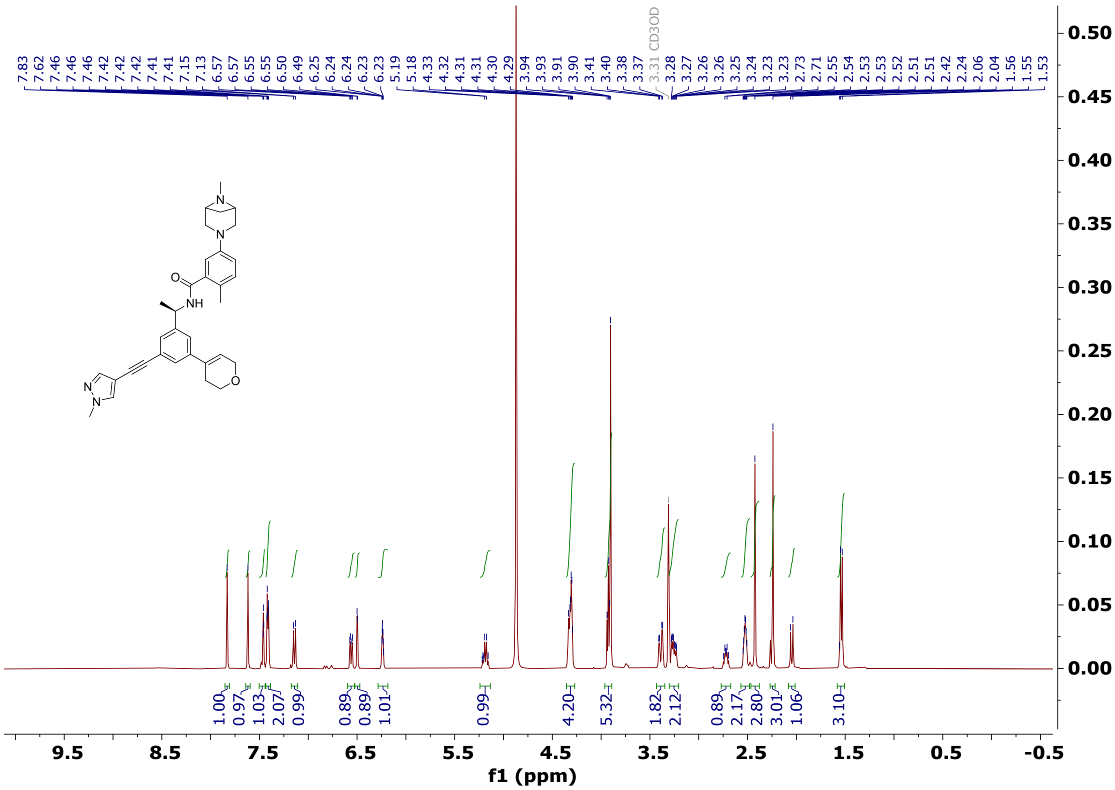
7.83 7.62 7.46 7.46 7.46 7.42 7.42 7.42 7.41 7.41 7.15 7.13 6.57 6.57 6.55 6.55 6.50 6.49 6.25 6.24 6.24 6.23 6.23 5.19 5.18 4.33 4.32 4.31 4.31 4.30 4.29 3.94 3.93 3.91 3.90 3.41 3.40 3.38 3.37 3.31 CD3OD 3.28 3.27 3.26 3.26 3.25 3.24 3.23 3.23 2.73 2.71 2.55 2.54 2.53 2.53 2.52 2.51 2.51 2.42 2.24 2.06 2.04 1.56 1.55 1.53
1.00 0.97 1.03 2.07 0.99 0.89 0.89 1.01 0.99 4.20 5.32 1.82 2.12 0.89 2.17 2.80 3.01 1.06 3.10
f1 (ppm)

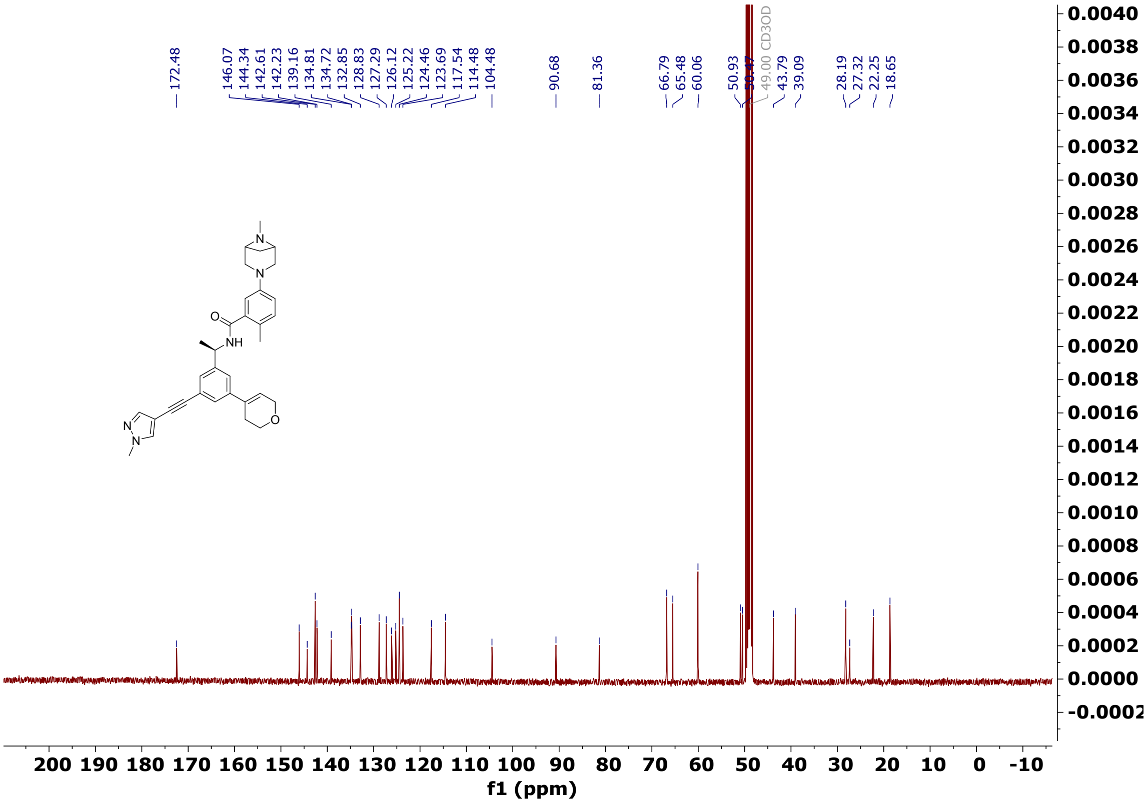
172.48 146.07 144.34 142.61 142.23 139.16 134.81 134.72 132.85 128.83 127.29 126.12 125.22 124.46 123.69 117.54 114.48 104.48 90.68 81.36 66.79 65.48 60.06 50.93 50.47 49.00 CD3OD 43.79 39.09 28.19 27.32 22.25 18.65
f1 (ppm)

Compound **21**

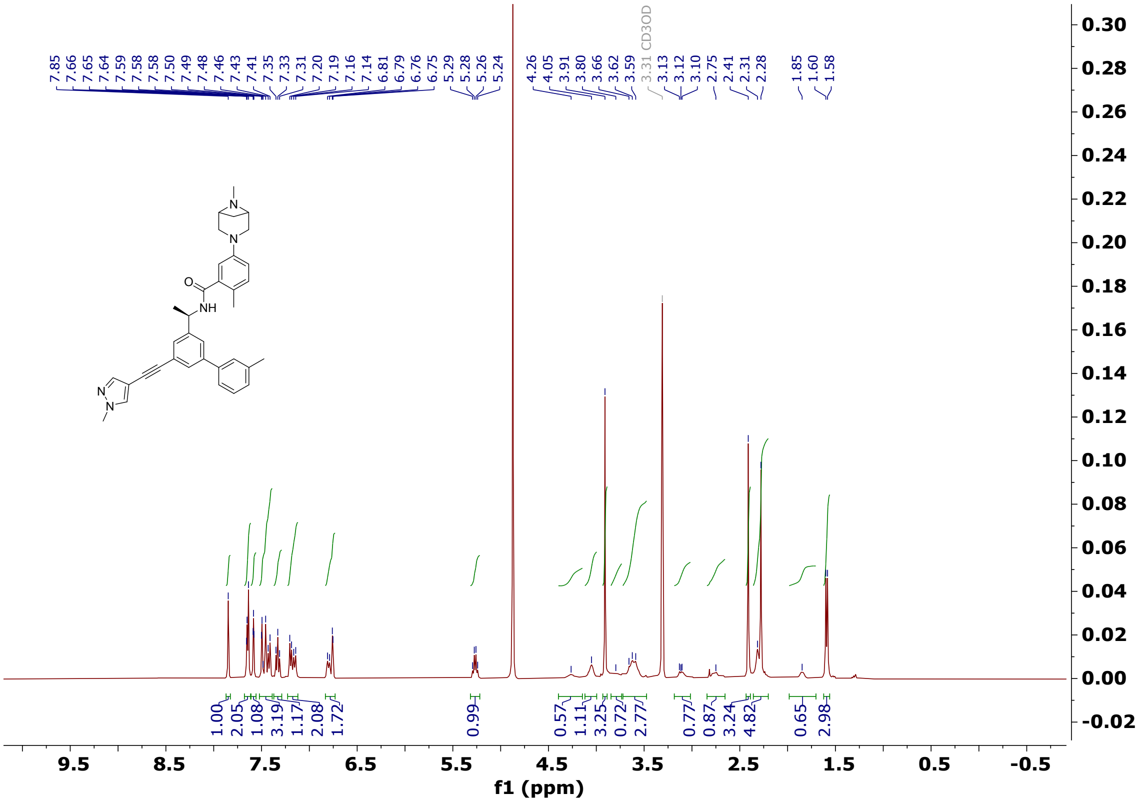

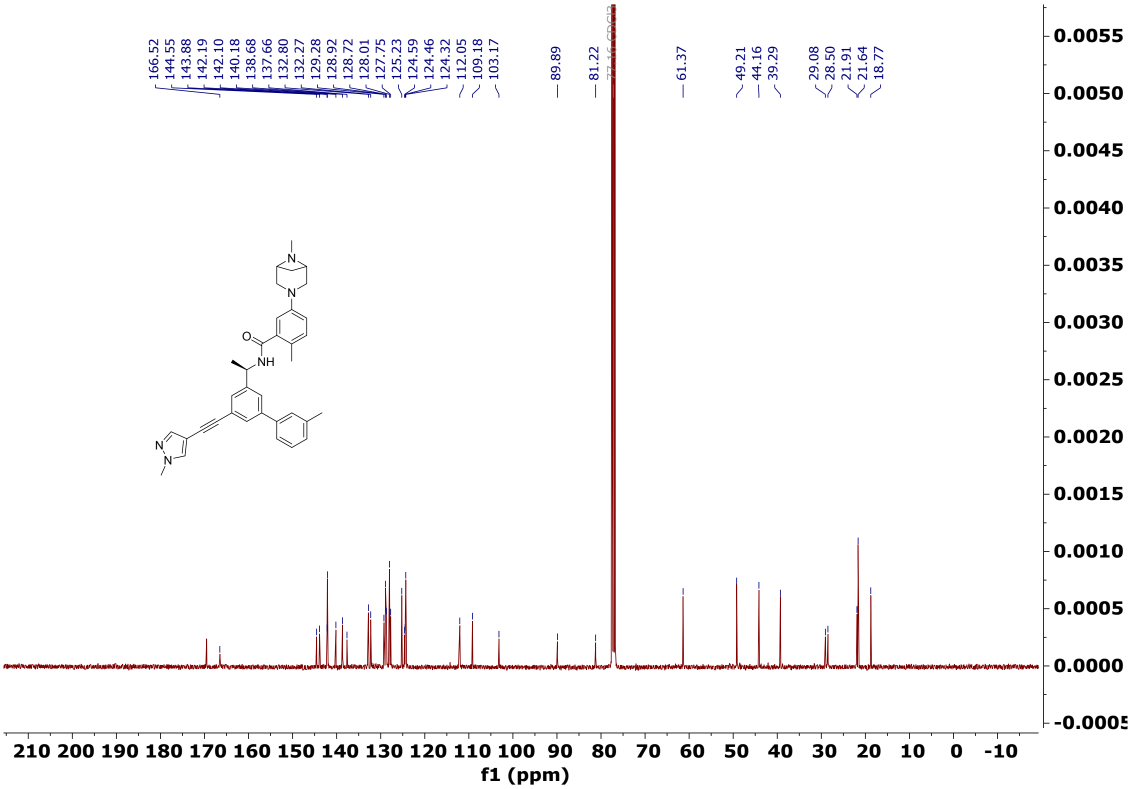

Compound **22**

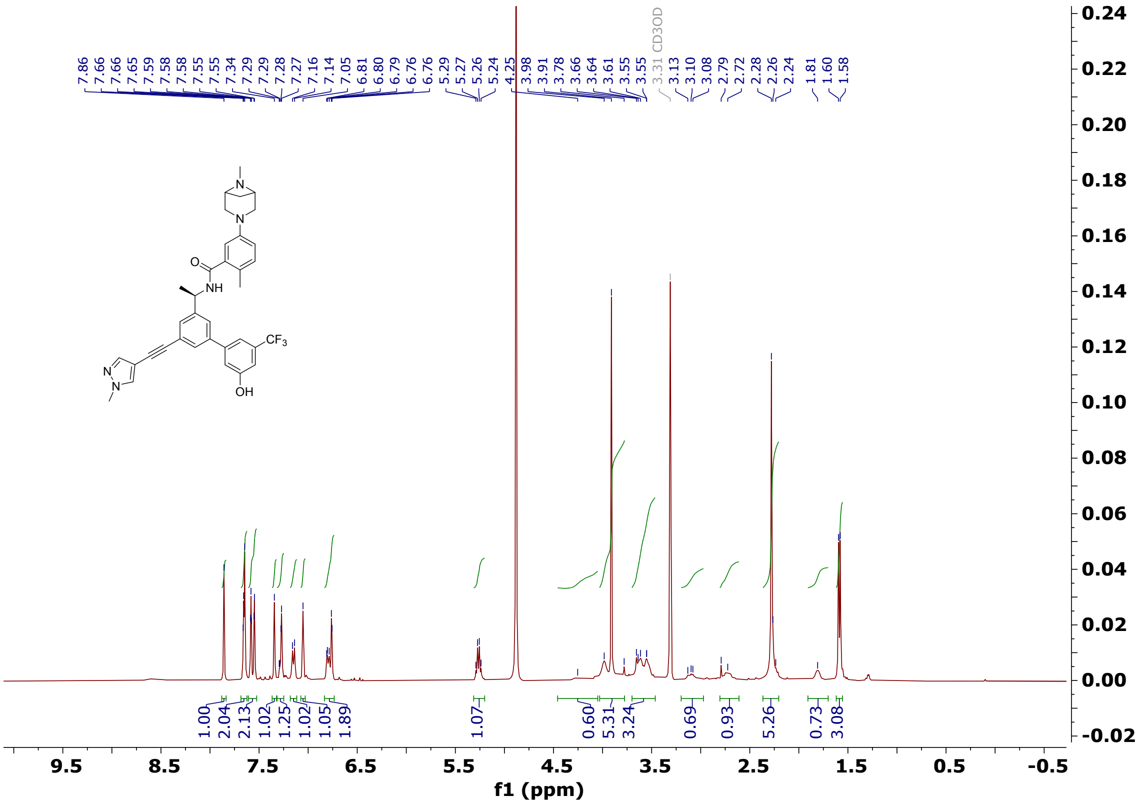

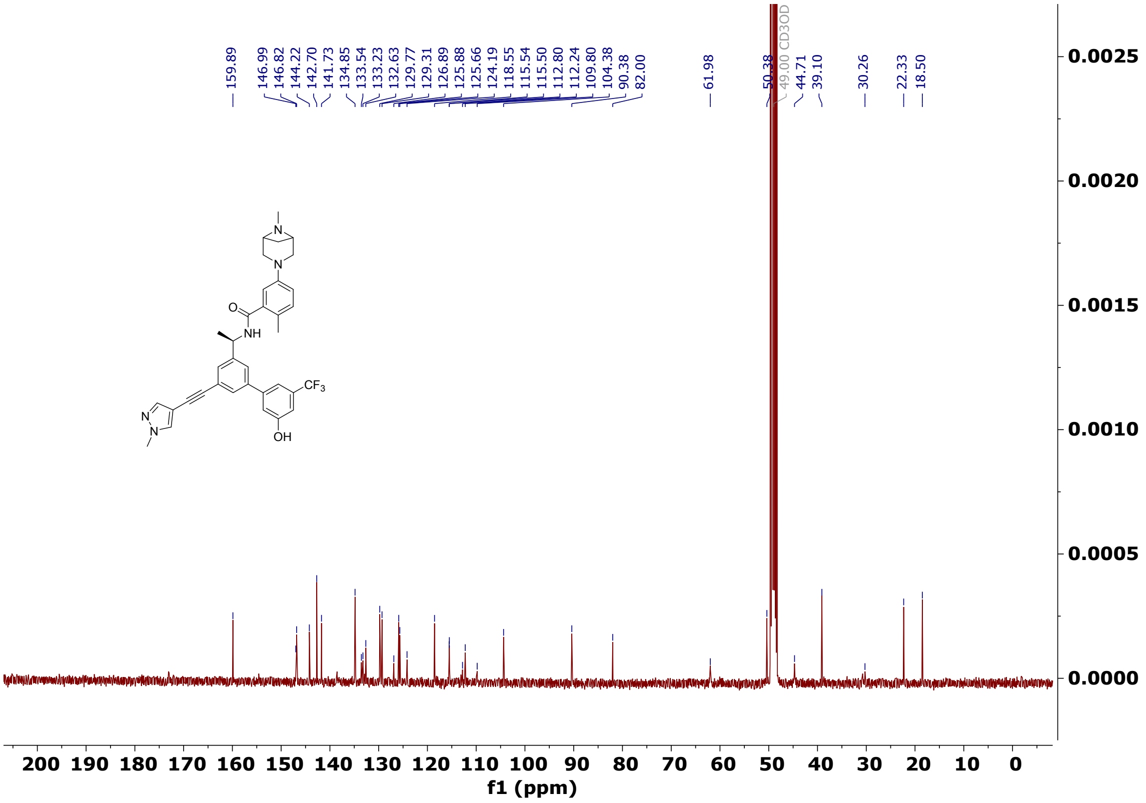
159.89
146.99
146.82
144.22
142.70
141.73
134.85
133.54
133.23
132.63
129.77
129.31
126.89
125.88
125.66
124.19
118.55
115.54
115.50
112.80
112.24
109.80
104.38
90.38
82.00
61.98
50.38
49.00 CD3OD
44.71
39.10
30.26
22.33
18.50
f1 (ppm)

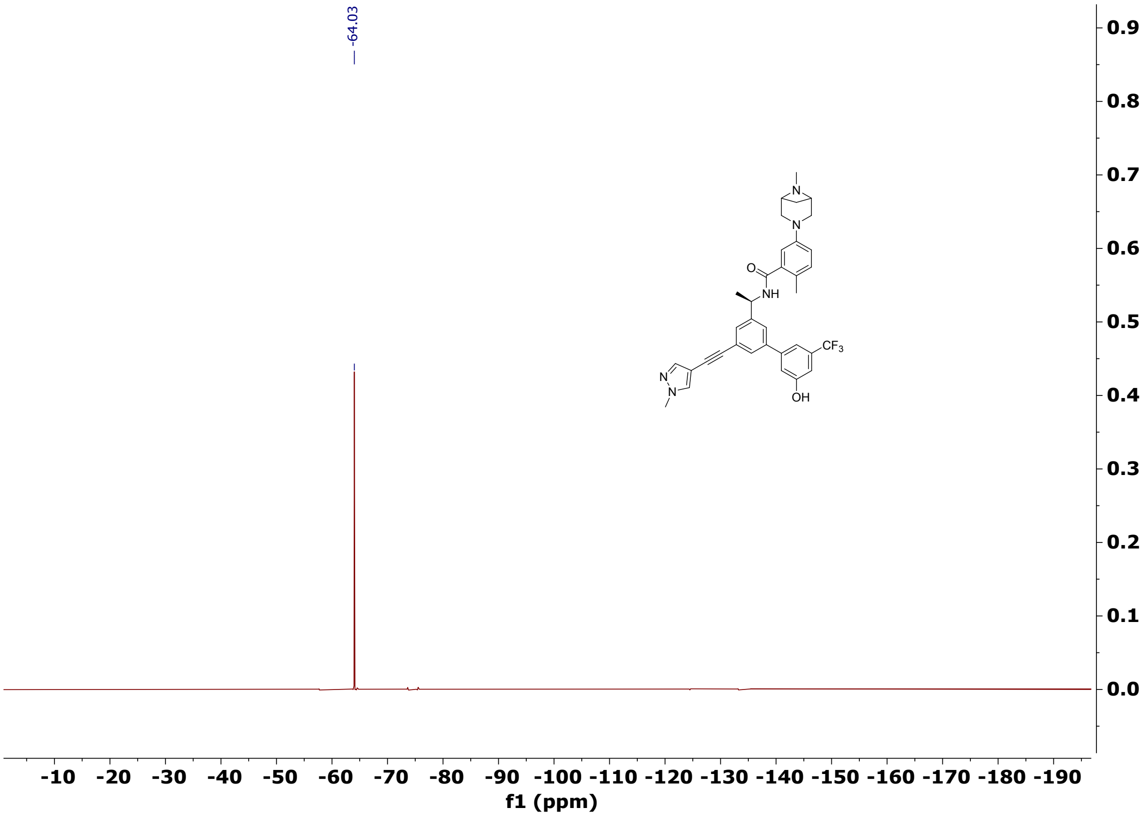
-64.03
f1 (ppm)

Compound **23**

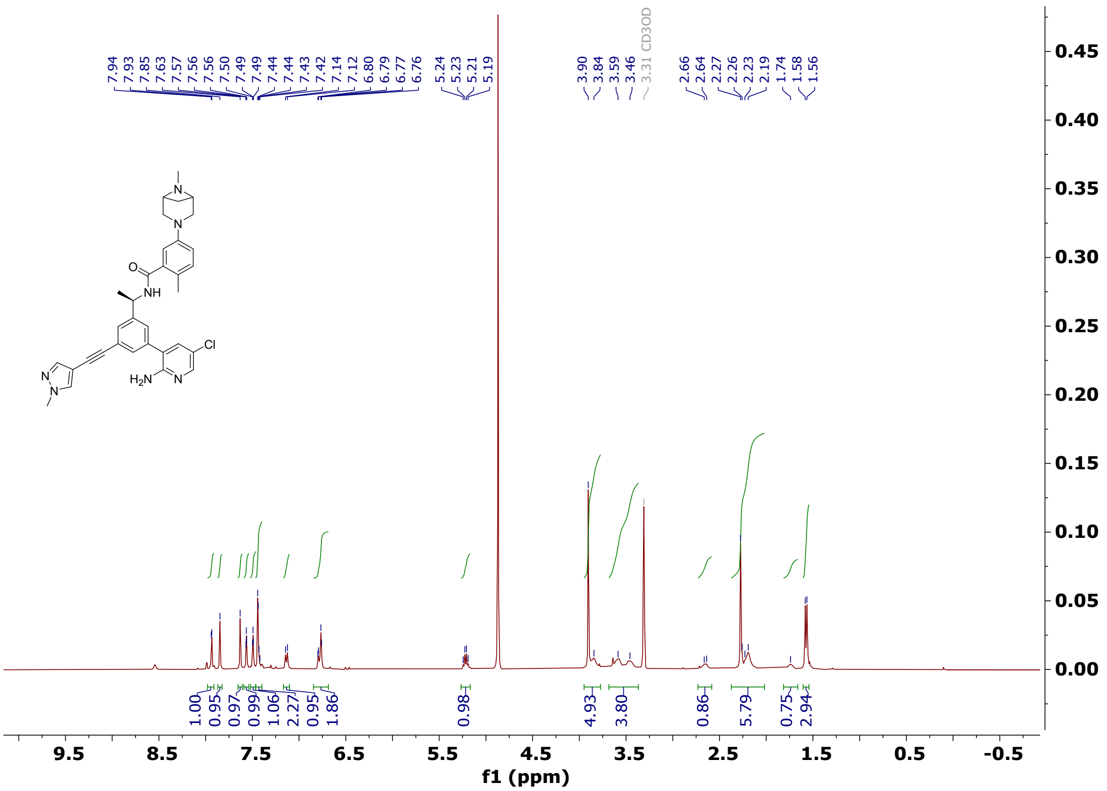

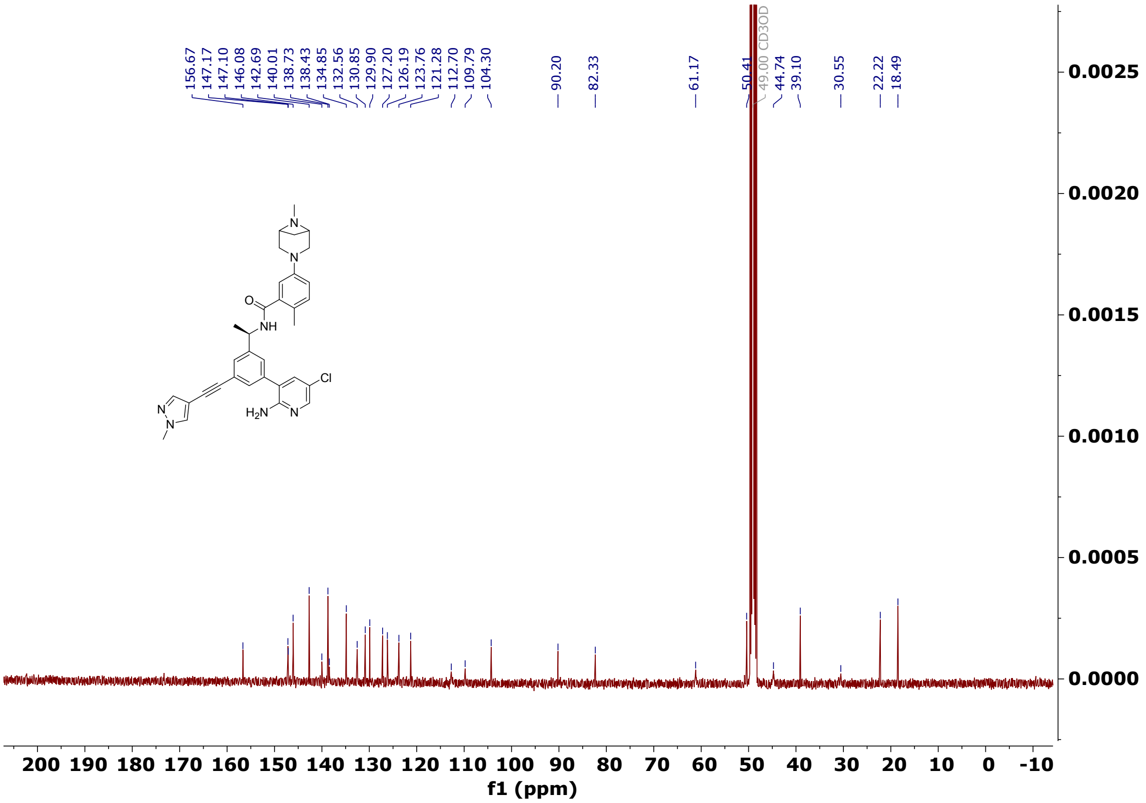

Compound **24**

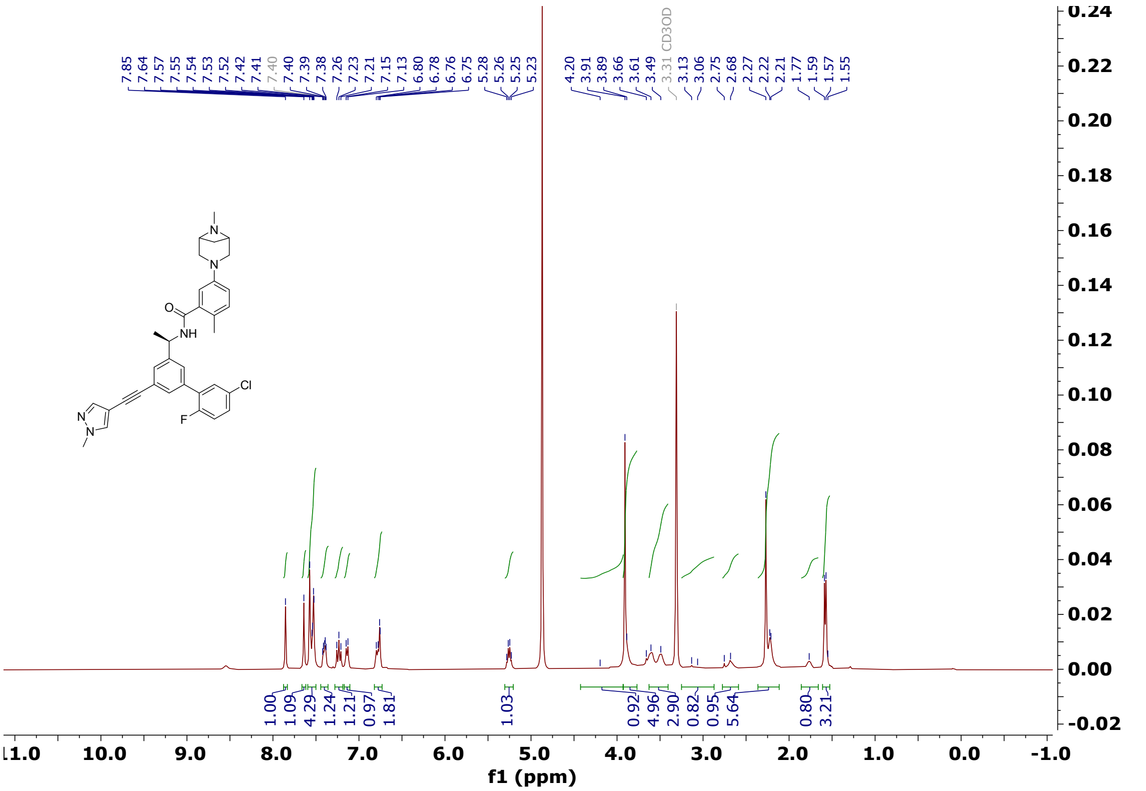
7.85 7.64 7.57 7.55 7.54 7.53 7.52 7.42 7.41 7.40 7.40 7.39 7.38 7.26 7.23 7.21 7.15 7.13 6.80 6.78 6.76 6.75 5.28 5.26 5.25 5.23 4.20 3.91 3.89 3.66 3.61 3.49 3.31 CD3OD 3.13 3.06 2.75 2.68 2.27 2.22 2.21 1.77 1.59 1.57 1.55
1.00 1.09 4.29 1.24 1.21 0.97 1.81 1.03 0.92 4.96 2.90 0.82 0.95 5.64 0.80 3.21
f1 (ppm)

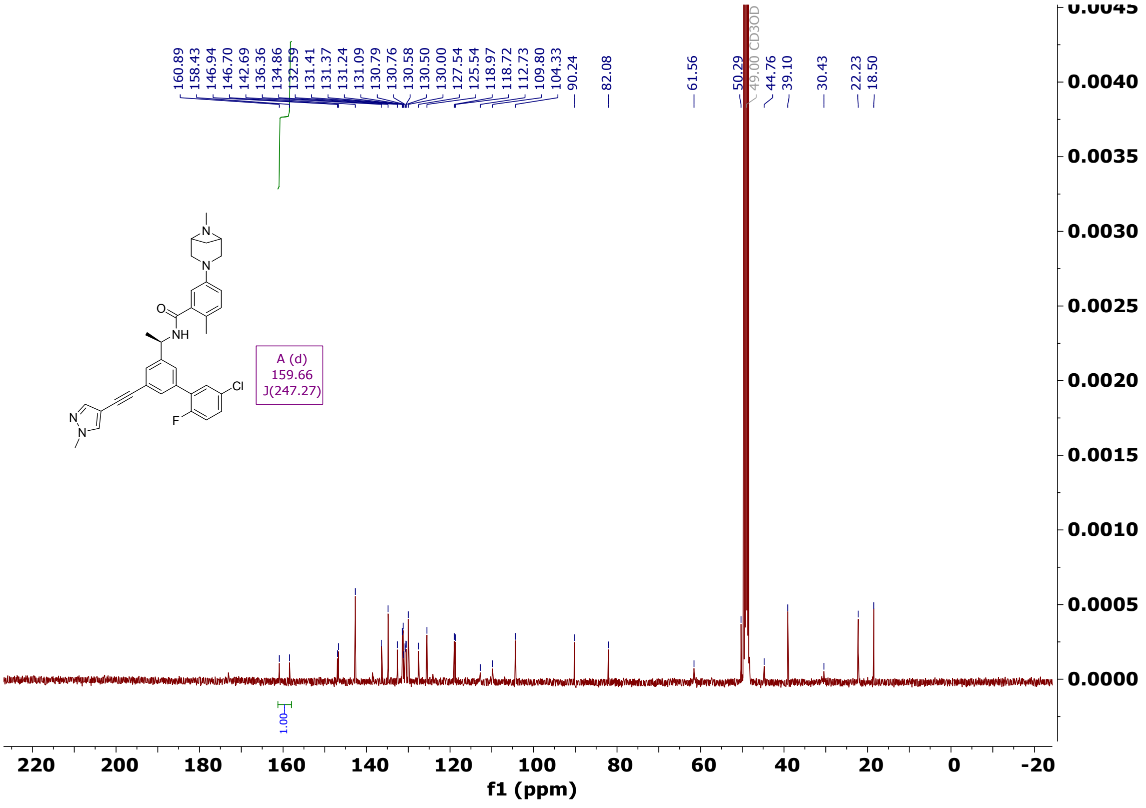
160.89 158.43 146.94 146.70 142.69 136.36 134.86 132.59 131.41 131.37 131.24 131.09 130.79 130.76 130.58 130.50 130.00 127.54 125.54 118.97 118.72 112.73 109.80 104.33 90.24 82.08 61.56 50.29 49.00 CD3OD 44.76 39.10 30.43 22.23 18.50
A (d)
159.66
J(247.27)
1.00
f1 (ppm)

Compound **25**

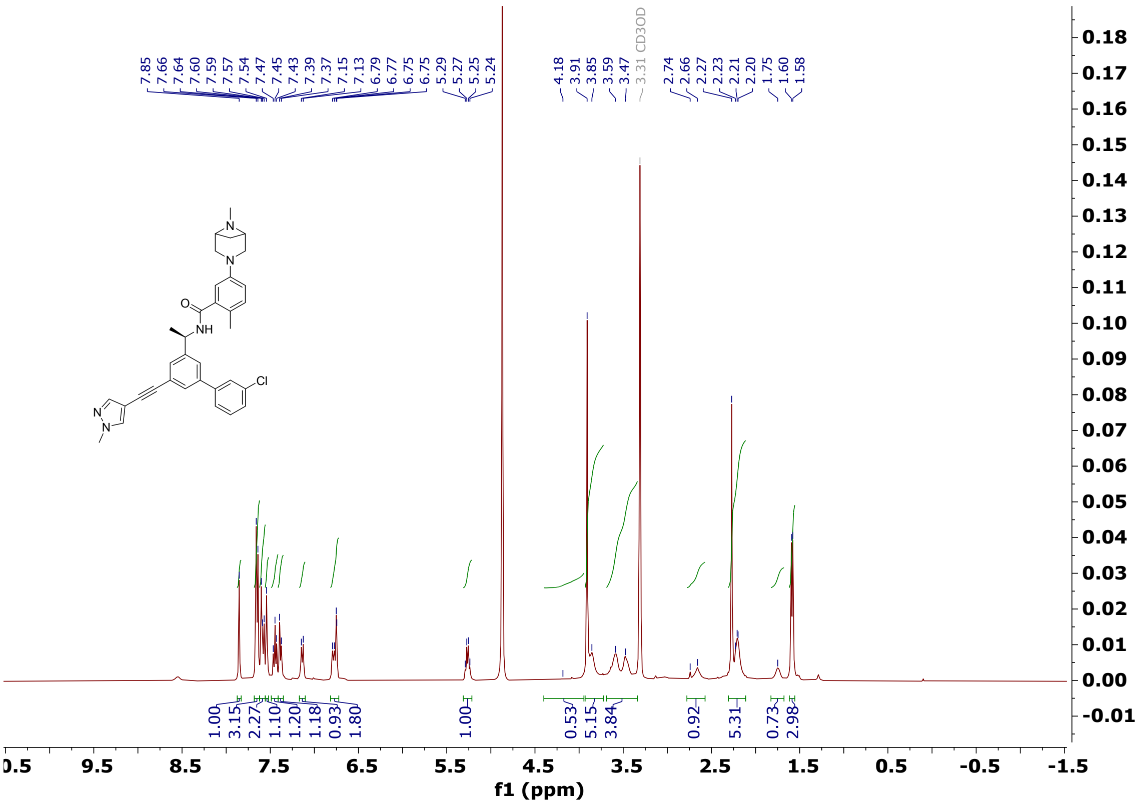
7.85
7.66
7.64
7.60
7.59
7.57
7.54
7.47
7.45
7.43
7.39
7.37
7.15
7.13
6.79
6.77
6.75
6.75
5.29
5.27
5.25
5.24
4.18
3.91
3.85
3.59
3.47
3.31 CD3OD
2.74
2.66
2.27
2.23
2.21
2.20
1.75
1.60
1.58
1.00
3.15
2.27
1.10
1.20
1.18
0.93
1.80
1.00
0.53
5.15
3.84
0.92
5.31
0.73
2.98
f1 (ppm)

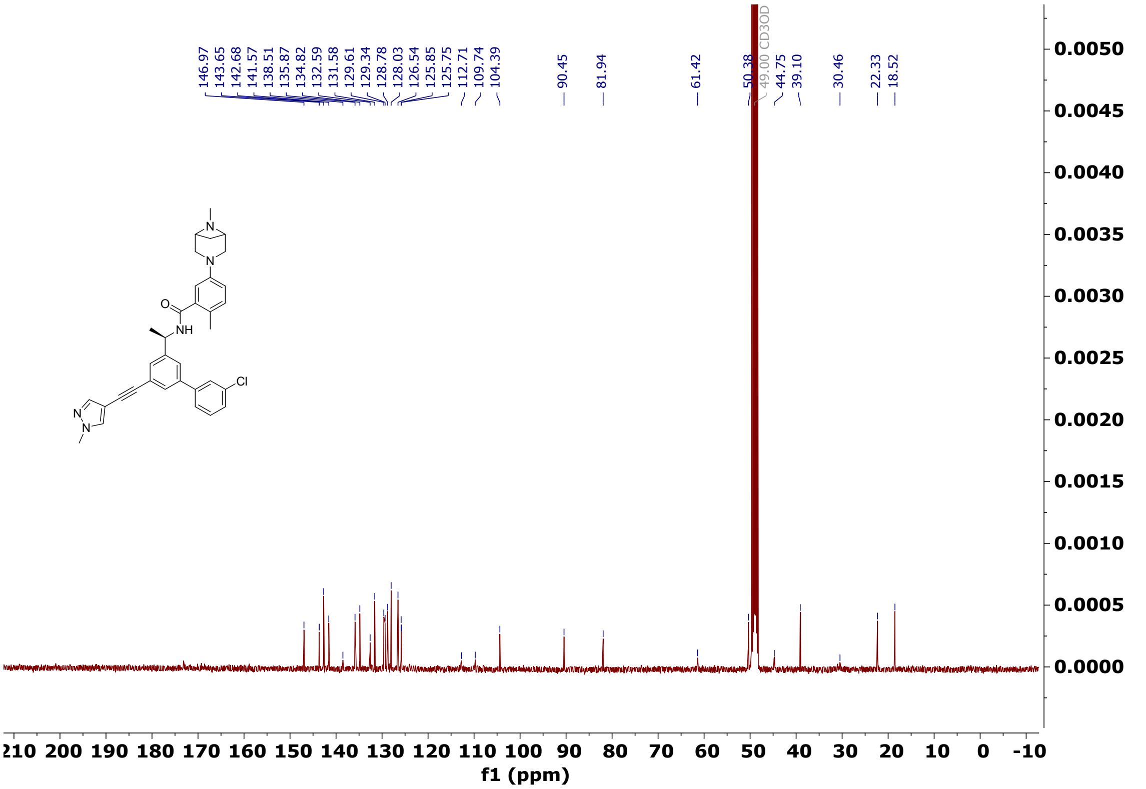
146.97
143.65
142.68
141.57
138.51
135.87
134.82
132.59
131.58
129.61
129.34
128.78
128.03
126.54
125.85
125.75
112.71
109.74
104.39
90.45
81.94
61.42
50.38
49.00 CD3OD
44.75
39.10
30.46
22.33
18.52
f1 (ppm)

Compound **26**

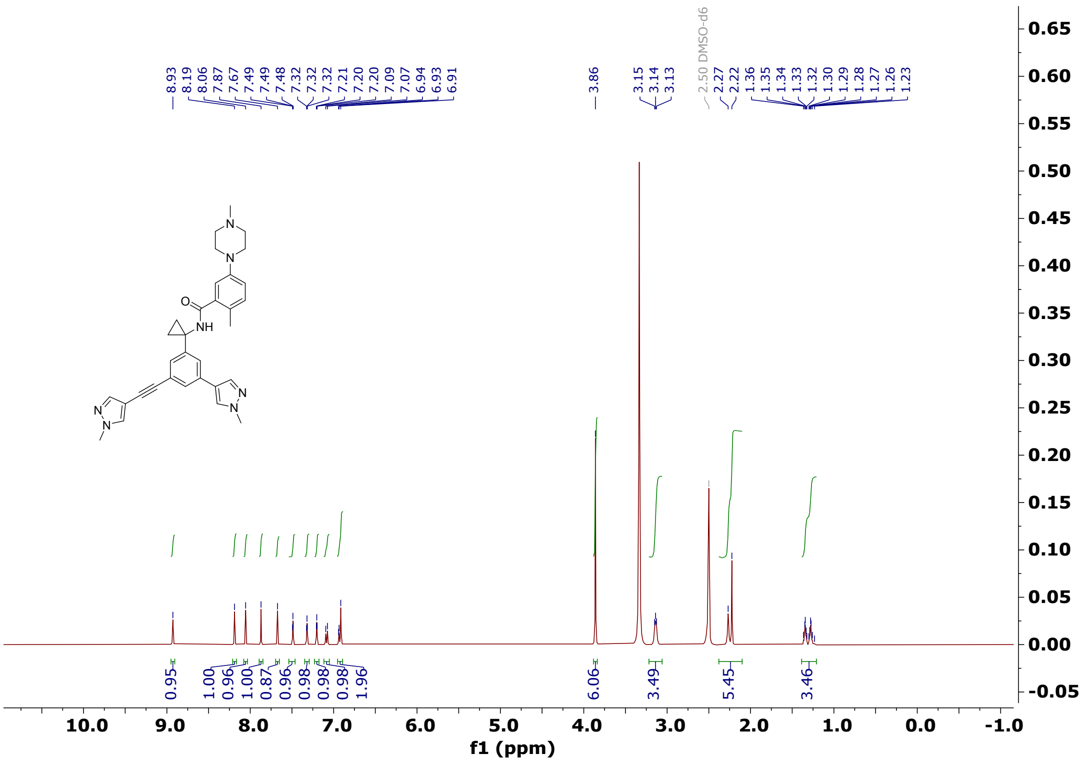
8.93 8.19 8.06 7.87 7.67 7.49 7.49 7.48 7.32 7.32 7.32 7.21 7.20 7.20 7.09 7.07 6.94 6.93 6.91
3.86
3.15 3.14 3.13
2.50 DMSO-d6
2.27 2.22
1.36 1.35 1.34 1.33 1.32 1.30 1.29 1.28 1.27 1.26 1.23
0.95 1.00 0.96 1.00 0.87 0.96 0.98 0.98 0.98 1.96
6.06 3.49 5.45 3.46
0.65 0.60 0.55 0.50 0.45 0.40 0.35 0.30 0.25 0.20 0.15 0.10 0.05 0.00 -0.05
10.0 9.0 8.0 7.0 6.0 5.0 4.0 3.0 2.0 1.0 0.0 -1.0
f1 (ppm)

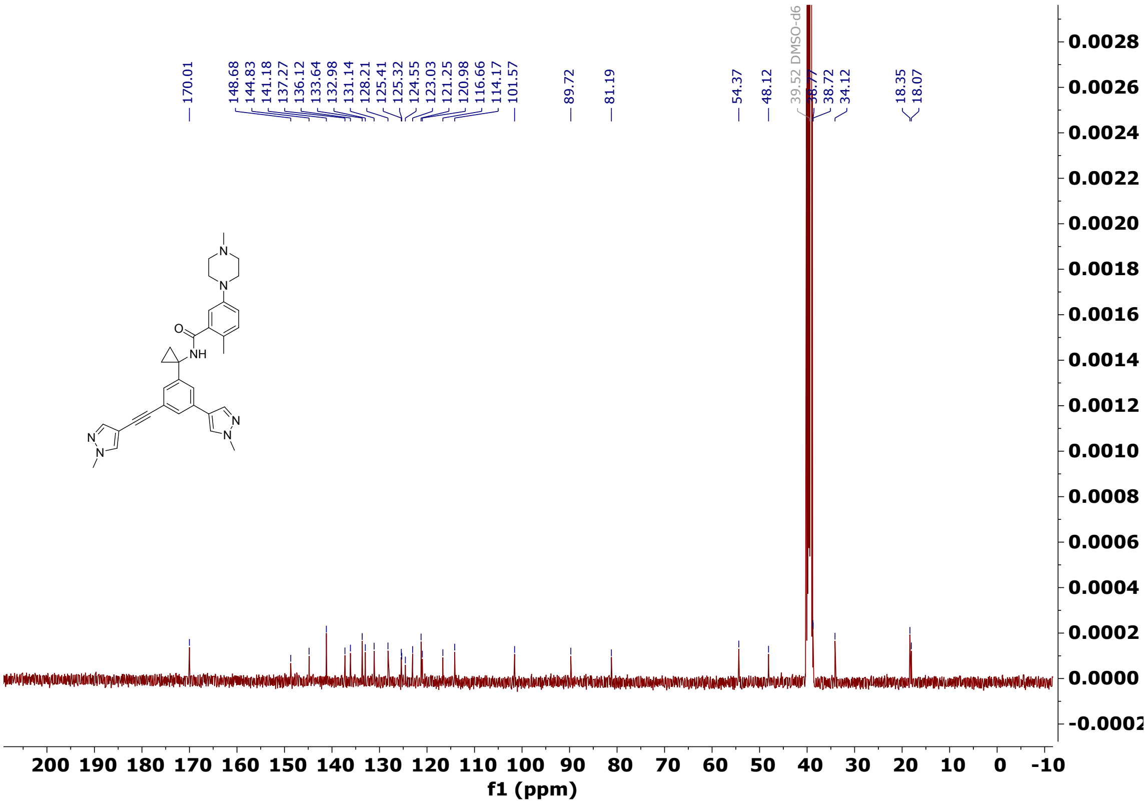
170.01
148.68 144.83 141.18 137.27 136.12 133.64 132.98 131.14 128.21 125.41 125.32 124.55 123.03 121.25 120.98 116.66 114.17 101.57
89.72 81.19
54.37 48.12
39.52 DMSO-d6
38.77 38.72 34.12
18.35 18.07
0.0028 0.0026 0.0024 0.0022 0.0020 0.0018 0.0016 0.0014 0.0012 0.0010 0.0008 0.0006 0.0004 0.0002 0.0000 -0.0002
200 190 180 170 160 150 140 130 120 110 100 90 80 70 60 50 40 30 20 10 0 -10
f1 (ppm)

HPLC Traces

Compound **9**

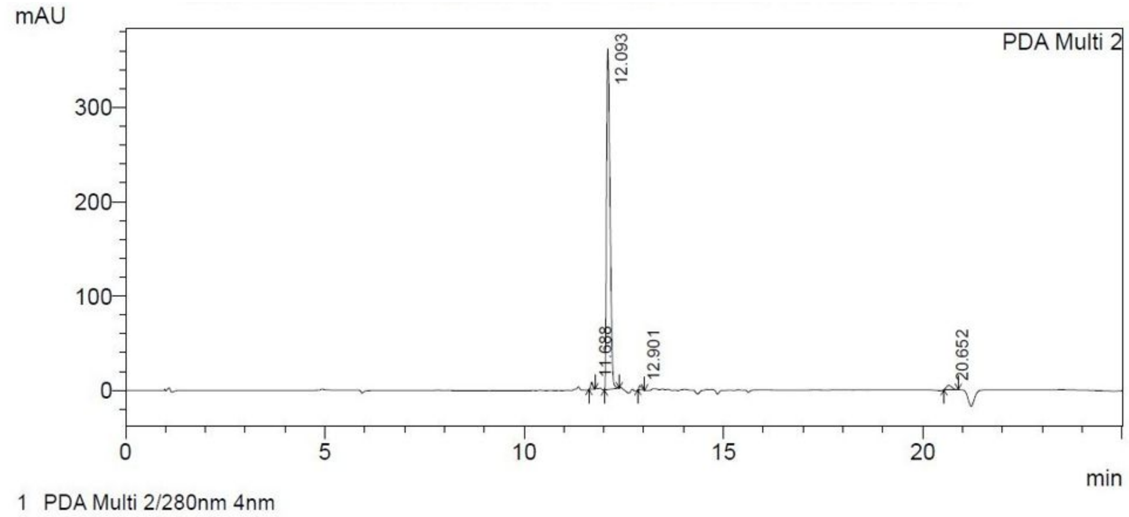

PeakTable

PDA Ch2 280nm 4nm

Peak#	Ret. Time	Area	Area %
1	11.688	28127	1.215
2	12.093	2221650	95.992
3	12.901	22900	0.989
4	20.652	41725	1.803
Total		2314402	100.000

Compound **10**

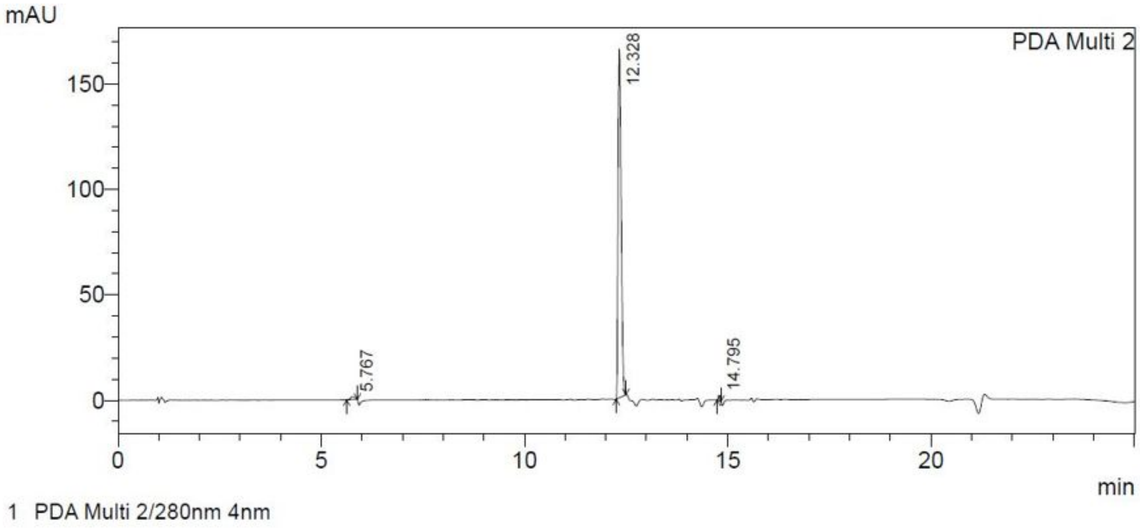

PeakTable

PDA Ch2 280nm 4nm

Peak#	Ret. Time	Area	Area %
1	5.767	10918	1.242
2	12.328	860439	97.859
3	14.795	7905	0.899
Total		879262	100.000

Compound **11**

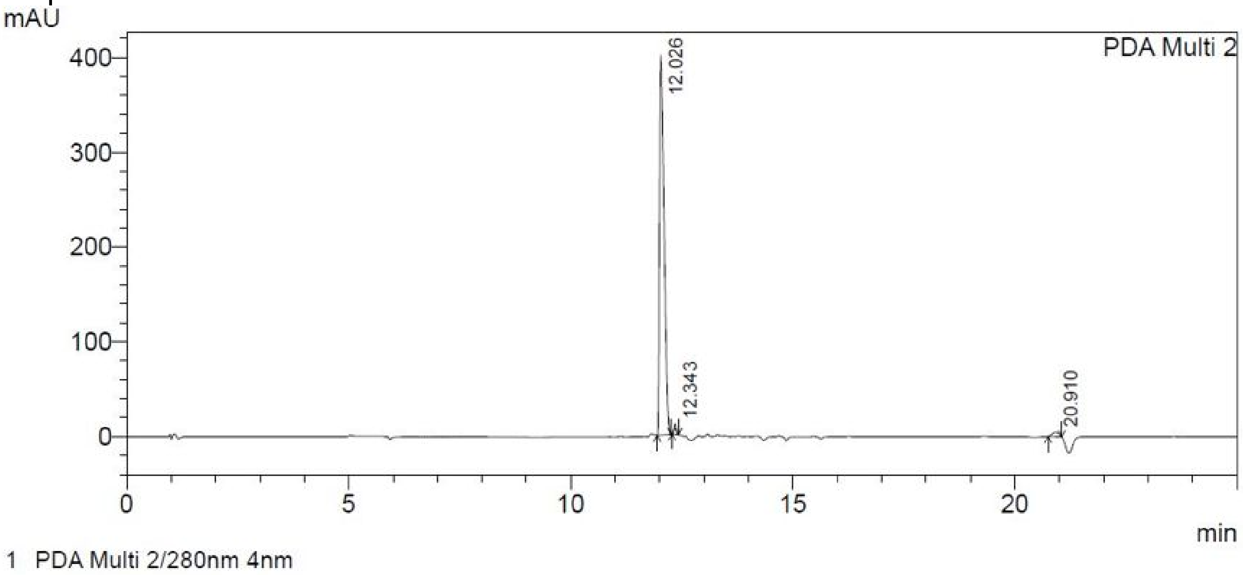

PeakTable

PDA Ch2 280nm 4nm

Peak#	Ret. Time	Area	Area %
1	12.026	2944447	97.095
2	12.343	42825	1.412
3	20.910	45277	1.493
Total		3032549	100.000

Compound **12**

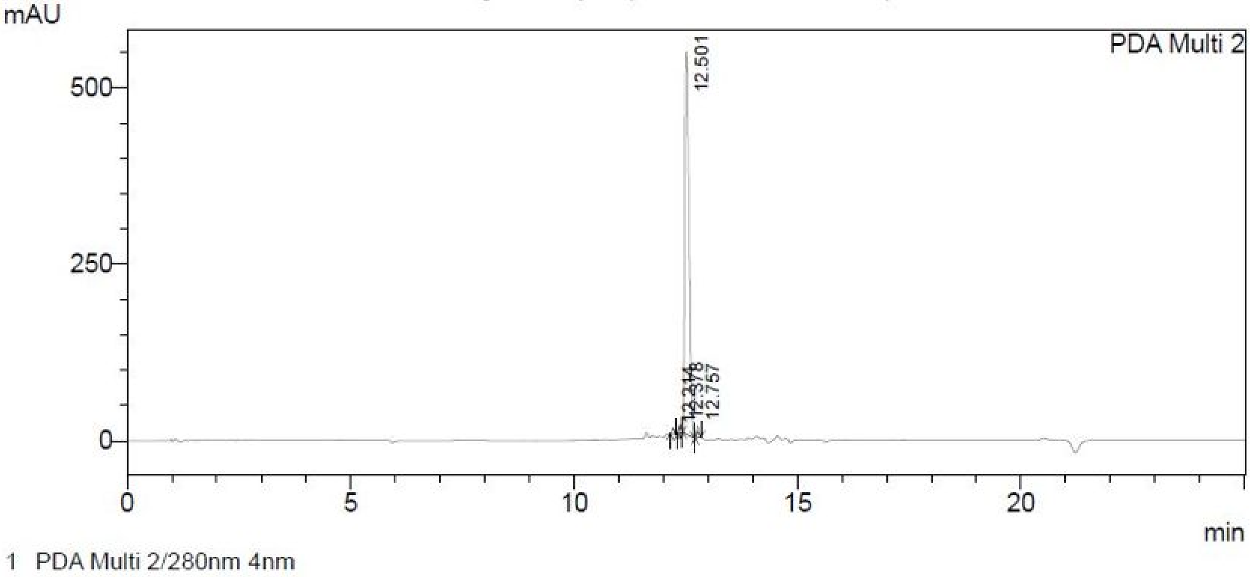

PeakTable

PDA Ch2 280nm 4nm

Peak#	Ret. Time	Area	Area %
1	12.214	24943	0.682
2	12.378	34423	0.941
3	12.501	3533733	96.572
4	12.757	66083	1.806
Total		3659182	100.000

Compound **13**

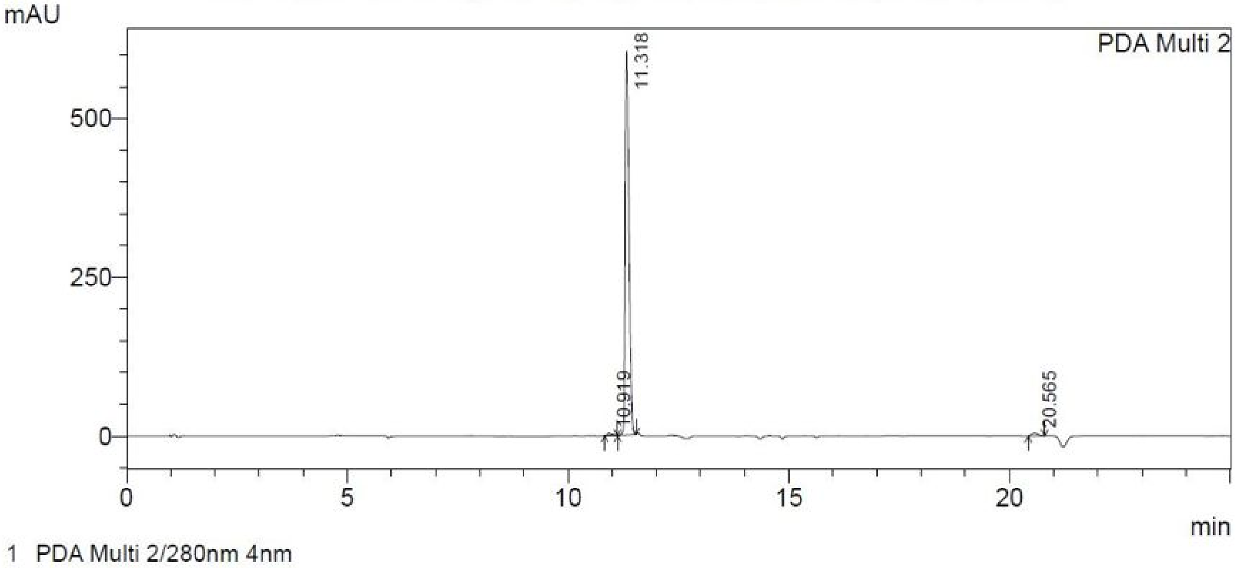

PeakTable

PDA Ch2 280nm 4nm

Peak#	Ret. Time	Area	Area %
1	10.919	31317	0.806
2	11.318	3812194	98.144
3	20.565	40778	1.050
Total		3884289	100.000

Compound **14**

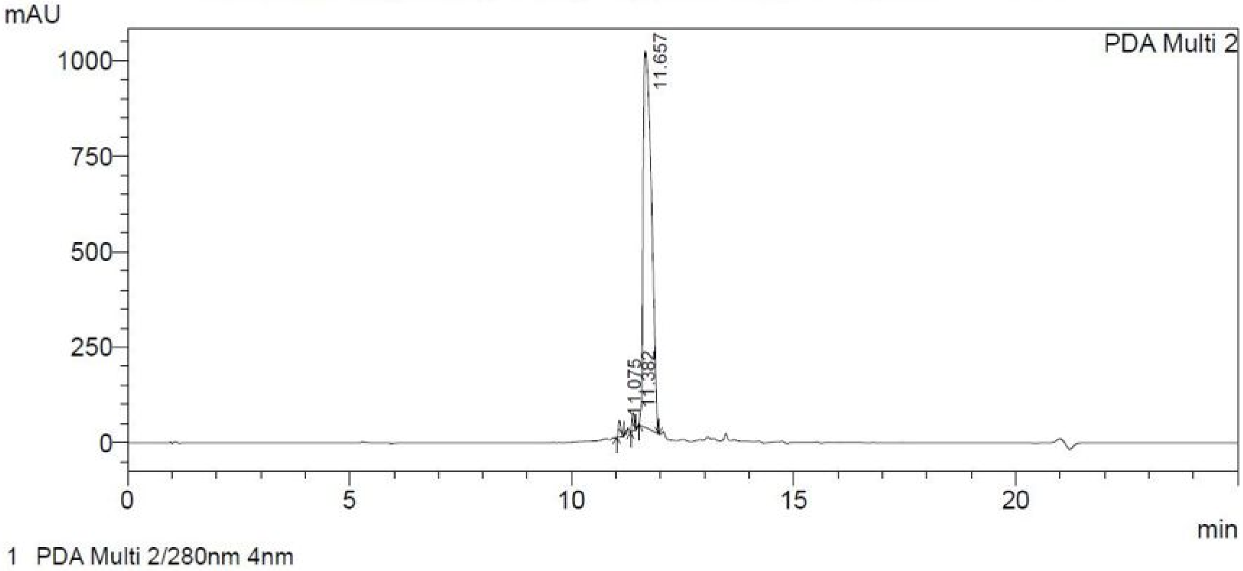

PeakTable

PDA Ch2 280nm 4nm

Peak#	Ret. Time	Area	Area %
1	11.075	174794	1.353
2	11.382	167783	1.299
3	11.657	12572704	97.348
Total		12915281	100.000

Compound **15**

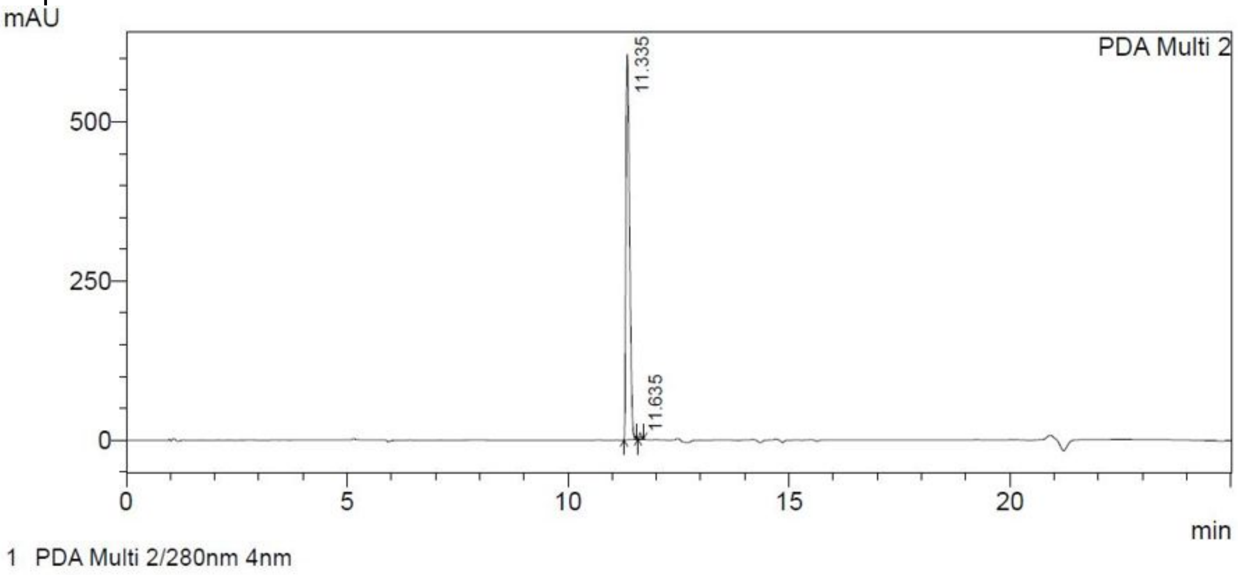

PeakTable

PDA Ch2 280nm 4nm

Peak#	Ret. Time	Area	Area %
1	11.335	3534897	99.695
2	11.635	10832	0.305
Total		3545729	100.000

Compound **19**

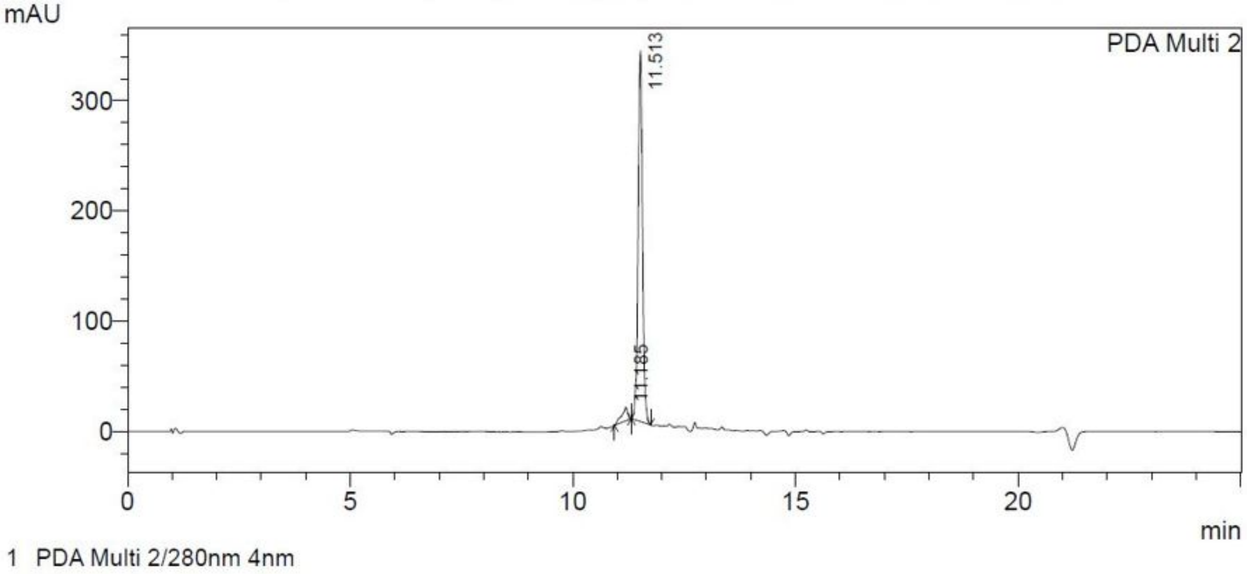

PeakTable

PDA Ch2 280nm 4nm

Peak#	Ret. Time	Area	Area %
1	11.185	116475	4.788
2	11.513	2316131	95.212
Total		2432605	100.000

Compound **22**

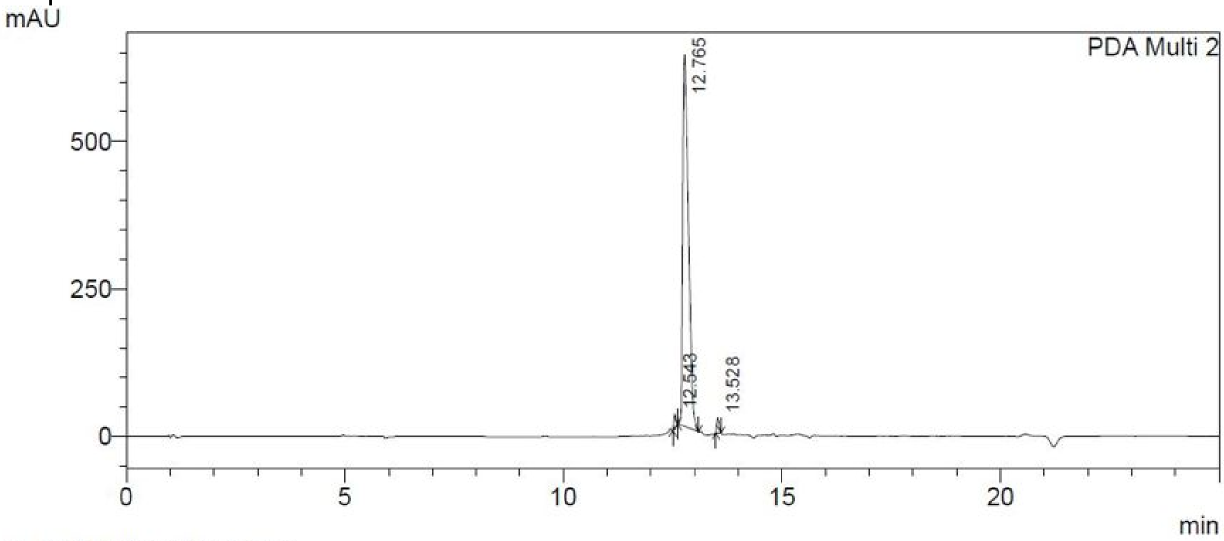

1 PDA Multi 2/280nm 4nm

PeakTable

PDA Ch2 280nm 4nm

Peak#	Ret. Time	Area	Area %
1	12.543	73683	1.257
2	12.765	5694024	97.151
3	13.528	93274	1.591
Total		5860981	100.000

Compound **24**

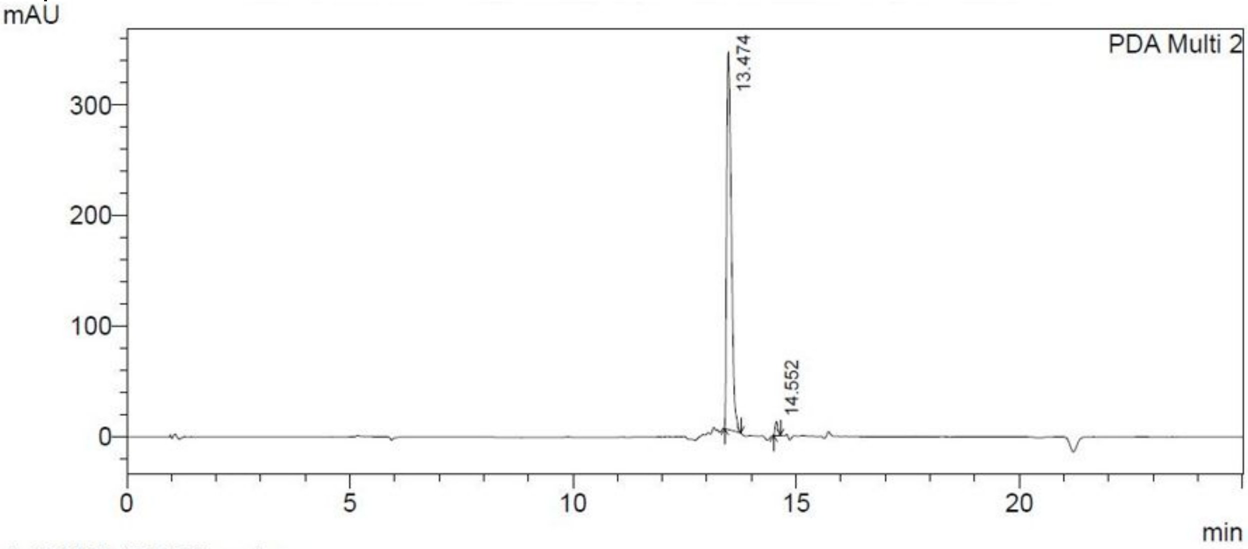

1 PDA Multi 2/280nm 4nm

PeakTable

PDA Ch2 280nm 4nm

Peak#	Ret. Time	Area	Area %
1	13.474	2435651	97.945
2	14.552	51094	2.055
Total		2486746	100.000

Compound **26**

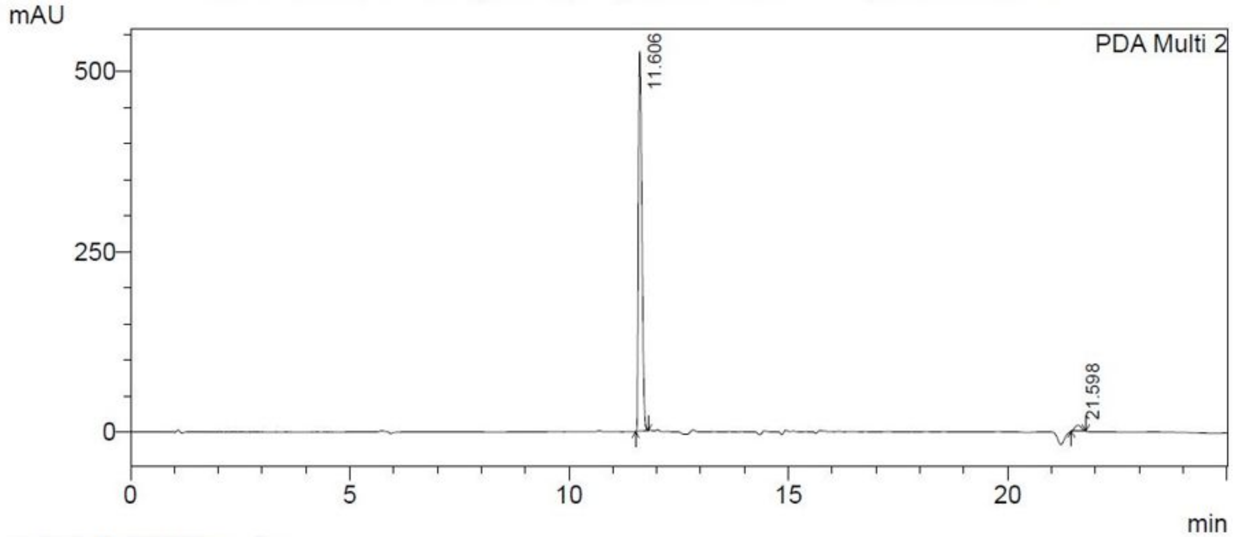

1 PDA Multi 2/280nm 4nm

PeakTable

PDA Ch2 280nm 4nm

Peak#	Ret. Time	Area	Area %
1	11.606	2861106	97.055
2	21.598	86804	2.945
Total		2947911	100.000
